# A systematic review of the clinical application of data-driven population segmentation analysis

**DOI:** 10.1186/s12874-018-0584-9

**Published:** 2018-11-03

**Authors:** Shi Yan, Yu Heng Kwan, Chuen Seng Tan, Julian Thumboo, Lian Leng Low

**Affiliations:** 1 Duke-NUS Medical School, 8 College Road, Singapore, 169857 Singapore; 2 Program in Health Services and Systems Research, Duke-NUS Medical School, 8 College Road, Singapore, 169857 Singapore; 3 Saw Swee Hock School of Public Health, National University of Singapore, 12 Science Drive 2, Singapore, 117549 Singapore; 4 Rheumatology and Immunology, Singapore General Hospital, 16 College Road, Block 6 Level 9, Singapore, 169854 Singapore; 5 Family Medicine and Continuing Care, Singapore General Hospital, Outram Road, Bowyer Block, Block A, Level 2, Singapore, 169608 Singapore

**Keywords:** Systematic review, Population segmentation, Data analytics, Population health, Public health, Health policy, Health services research

## Abstract

**Background:**

Data-driven population segmentation analysis utilizes data analytics to divide a heterogeneous population into parsimonious and relatively homogenous groups with similar healthcare characteristics. It is a promising patient-centric analysis that enables effective integrated healthcare interventions specific for each segment. Although widely applied, there is no systematic review on the clinical application of data-driven population segmentation analysis.

**Methods:**

We carried out a systematic literature search using PubMed, Embase and Web of Science following PRISMA criteria. We included English peer-reviewed articles that applied data-driven population segmentation analysis on empirical health data. We summarized the clinical settings in which segmentation analysis was applied, compared and contrasted strengths, limitations, and practical considerations of different segmentation methods, and assessed the segmentation outcome of all included studies. The studies were assessed by two independent reviewers.

**Results:**

We retrieved 14,514 articles and included 216 articles. Data-driven population segmentation analysis was widely used in different clinical contexts. 163 studies examined the general population while 53 focused on specific population with certain diseases or conditions, including psychological, oncological, respiratory, cardiovascular, and gastrointestinal conditions. Variables used for segmentation in the studies are heterogeneous. Most studies (*n* = 170) utilized secondary data in community settings (*n* = 185). The most common segmentation method was latent class/profile/transition/growth analysis (*n* = 96) followed by K-means cluster analysis (*n* = 60) and hierarchical analysis (*n* = 50), each having its advantages, disadvantages, and practical considerations. We also identified key criteria to evaluate a segmentation framework: internal validity, external validity, identifiability/interpretability, substantiality, stability, actionability/accessibility, and parsimony.

**Conclusions:**

Data-driven population segmentation has been widely applied and holds great potential in managing population health. The evaluations of segmentation outcome require the interplay of data analytics and subject matter expertise. The optimal framework for segmentation requires further research.

**Electronic supplementary material:**

The online version of this article (10.1186/s12874-018-0584-9) contains supplementary material, which is available to authorized users.

## Background

Globally, there has been a growing interest in population health and integrated health systems, which aim to organize health services across the care continuum around the needs of individuals, with the ultimate goal to improve the overall health of population by more targeted, effective, and coordinated healthcare services [1–3]. This patient-centered approach empowers healthcare systems to have a deeper understanding of population heath needs, prioritize health intervention programs, and facilitate effective and targeted healthcare resource planning [4]. Given the global trend of rapidly aging populations and mounting chronic disease burden, the management of population health becomes challenging in view of increasing healthcare services utilization and escalating health-related expenditure, making healthcare resources increasingly strained [5–7]. Therefore, it is imperative to develop more effective healthcare models with health initiatives that are tailored to the specific healthcare needs of a population [8–10].

While it is practically prohibitive, at population health policy level, to address each individual’s widely different care needs in a heterogeneous population, they can be segmented into distinct subgroups, each of which has relatively homogeneous health characteristics and physical, psychological, and social needs [8]. This concept of population segmentation allows population health policies to develop and organize around these population segments, with different care programs tailored to each segment [11]. In a healthcare system, population segmentation analysis can facilitate more effective healthcare resource planning and evidenced-based policy making [12]. A recent study that followed 200 patients in a program that used segmentation to deliver highly tailored health interventions for one year showed a 32% reduction in the utilization of emergency care with high level of patient satisfaction [13].

Broadly, two major approaches for population segmentation have emerged over the years. Expert-driven approaches segment a population by a-priori, experts-defined criteria based on literature review and consensus, while data-driven approaches utilize *post-hoc* statistical analysis such as clustering analysis and latent class analysis on empirical data. For example, the Clinical Risk Group system by 3 M classifies patient population into one of over 200 mutually exclusive risk groups based on an expert-defined hierarchical system of classification where greater weightage is given to patients’ highest morbidity diseases [14]. Senior Segmentation Algorithm developed by Kaiser Permanente for elderly persons is another example of expert-driven population segmentation whereby population is divided into “robust seniors without chronic conditions”, “seniors with one or more chronic conditions”, “seniors with advanced illness and end-organ failure”, “seniors with advanced frailty or at the end of life” groups [12]. As expected, there is no widely generalizable expert-defined criteria on determining the optimal number of segments, selecting the variables to be used for segmentation, and defining the segments for different populations of interest.

More recently, the wide adoption of electronic health records (EHRs) in healthcare systems, coupled with the advancement in big data analytics, makes rich healthcare data more accessible and provides opportunities to utilize empirical data for population segmentation analysis [8]. Data-driven population segmentation is increasingly gaining interest as it generates detailed and quantitative insights from large volumes of population healthcare data that support evidence-based policy decisions on population health [6]. For example, Van der Laan et al. applied latent class analysis on self-reported biological, psychological, functional and social variables to segment a general elderly population and demonstrated differential healthcare service utilization patterns across segments [7]. A recent paper by Vuik et al. also demonstrated the utility of data-driven clustering analysis to segment a general patient population using healthcare utilization data from administrative databases [6].

Given that the data-driven population segmentation is gaining more popularity and its potential value is increasingly appreciated, more studies are expected to emerge in the field of population health in the near future. However, there is little consensus, if any, on the optimal segmentation approach or framework. This paper aims to systematically retrieve and review the existing literature on the clinical application of data-driven population segmentation analysis and summarize the populations of interest subject to segmentation analysis, the variables used for segmentation and their data sources, the various segmentation objectives and methods, and the evaluation of derived segmentation outcome.

## Methods

### Study design

We performed a systematic review in accordance to the Preferred Reporting Items for Systematic review and Meta-Analysis (PRISMA) checklist. Our study did not involve human subjects and is exempted from Institutional Review Board approval.

### Search strategies

**Fig. 1:**
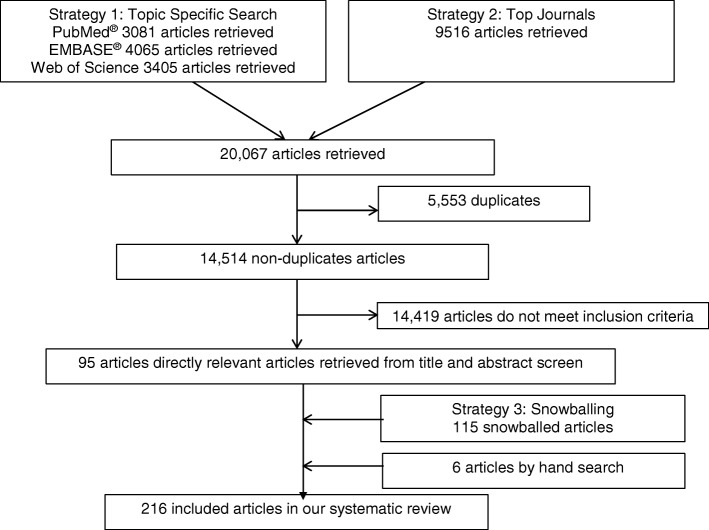
Flow chart of retrieval of articles

We employed three arm search strategies to obtain a comprehensive capture of potentially relevant articles for this systematic review: literature databases, top journals, and snowballing (Fig. 1). First, the PubMed, Embase, and Web of Science databases were searched from 1965 up to 15th November 2017. In our search strategy, we included the key terms typolog* OR stratif* OR segment* OR categor* OR “cluster analysis” OR cluster* OR pattern* OR profil* OR phenotyp* OR class* OR partition*. We applied these search terms in the PubMed® Topic Specific Query “Population Health” category (Additional file 1). The same search strategy was adapted for Embase and Web of Science. As a second search strategy, we searched amongst top 50 journals in public health and top 3 journals in population health (top journals) according to impact factors in 2016 by SCImago Scientific Journal Rankings and InCites Journal Citation Reports (Additional file 2). Thirdly, we manually reviewed the bibliographies of all eligible full-text studies from the first two search strategies to identify additional or missing studies (snowballing). Further hand searches were also conducted.

### Eligibility criteria

The inclusion criteria were full-text original studies published in English that used data-driven population segmentation analysis on empirical data. An important inclusion criterion to meet is that the segmentation methods had to be applied to real-life datasets and not theoretical, hypothetical, or simulated ones. Meta-analysis, case series, case reports and reviews were excluded. Articles that were not in English, not on human subjects, and articles that are solely expert-driven were also excluded.

### Selection of studies

After duplicate articles were removed using the EndNote, version X8, two independent researchers (S.Y. and Y.H.K.) reviewed the abstracts of retrieved studies for inclusion and discussed when discrepancies arose. Next, the full-text studies were independently read and assessed for eligibility by the two researchers. In the case of disagreement in the selection process, any discord was resolved by discussion with an independent researcher (L.L.L.).

### Data extraction

Once the article was deemed to be eligible, data from eligible articles were extracted independently by two researchers (S.Y. and Y.H.K.) using a standardized data extraction format. The following data were extracted from the included studies: title, year of publication, populations studied (including population size and country), segmentation objectives, variables used for segmentation (including data sources and settings of study), segmentation methods used, number of segments derived and variables used for external validation of segmentation outcome.

### Data presentation

We summarized the characteristics of target populations in the included articles, including population inclusion criteria, sample size, and geographical region. This gave an overview of populations on which data-driven segmentation analysis has been applied. We also presented the data sources (primary data or secondary) and settings (community-based or from healthcare institutions) to facilitate future research data acquisition for population segmentation. We identified themes of segmentation objectives to present an overview of the clinical application and utility of data-driven segmentation. We summarized the major advantages and disadvantages of commonly used statistical methods for population segmentation (used by more than 10 studies included in this systematic review) to aid future researchers in the selection of these methods. Finally, we presented a set of criteria useful to evaluate the quality of segmentation results of the included articles.

### Data availability statement

All data generated or analysed during this study are included in this published article and its supplementary information files.

## Results

As shown in Fig. 1, 14,514 articles were retrieved from Search Strategy 1 and 2 after removing 5553 duplicates. After an abstract screen, 14,419 articles that do not fulfill inclusion criteria were removed, yielding 95 articles. Snowballing was carried out for the 95 articles identified, which yielded 115 additional articles. The hand search added 6 more articles. The final number of articles included for full text review was 216. The percentage agreement between S.Y. and Y.H.K. was 90%. The information on populations of interest subject to segmentation analysis, includes population size, country/region, data sources and study settings are summarized in (Additional file 3). The objective of segmentation, variables used for segmentation, statistical methods and software packages, number of derived segments and their names are presented in (Additional file 4).

### Population studied

As shown in Table 1, the studies can be broadly classified into those that segment the general population and those that target specific populations with certain diseases or conditions (e.g. individuals who have respiratory conditions such as asthma patients). Majority of the studies (*n* = 163) included general population as the target population for segmentation. For example, Conry et al. segmented a cross-sectional, nationally representative sample of individuals aged 18 years and over by their health behaviors [15]. Other studies (*n* = 53) restricted the populations to the individuals with specific diseases or conditions. These studies can be further categorized into those that included individuals that have psychological problems (*n* = 12), cancer (*n* = 9), respiratory conditions (*n* = 8), heart diseases (n = 5), gastrointestinal conditions (*n* = 3), HIV infections (n = 3), and other diseases and conditions (*n* = 13). As an example, Pietrzak et al. only included adults with the diagnosis of post-traumatic stress disorder from the US National Epidemiologic Survey on Alcohol and Related Conditions [16].

**Table 1:** Characteristics of target population subjected to data-driven segmentation (*n* = 216)

Population selection	No. of studies	Examples
Without specific diseases/conditions	163	A nationally representative sample of adults aged 18 years and over in Ireland [15]
With specific diseases/conditions	53	
Patients with psychological conditions	12	US adults who were diagnosed with lifetime post-traumatic stress disorder in wave 2 of the National Epidemiologic Survey on Alcohol and Related Conditions [16]
Patients with cancer	9	Consecutive referrals with a diagnosis of non-curable cancer to the Palliative Medicine Program at the Cleveland Clinic Foundation [67]
Patients with respiratory conditions	8	Children 6–17 years of age who underwent standardized characterization in Severe Asthma Research Program [68]
Patients with heart diseases	5	Elderly patients admitted with ischemic coronary heart disease and recruited in a clinical trial [69]
Patients with HIV positive status	3	A random stratified sample of HIV/AIDS patients recruited in French hospital departments delivering HIV care [70]
Patients with gastrointestinal conditions	3	Patients with intractable irritable bowel syndrome enrolled in a randomised controlled trial [71]
Others	13	
Sample Size		
< =500	49	
501–1000	41	
1001-10,000	87	
10,001–100,000	24	
> =100,001	10	
N.A.	5	
Country/Region		
Multiple countries	11	
North America	122	
US	109	
Canada	13	
Europe	60	
UK	24	
Other European countries	36	
Asia	13	
Oceanian	8	
Africa	2	

Abbreviations: *HIV* Human Immunodeficiency Virus, *AIDS* Acquired Immune Deficiency syndrome, *US* The United States of America, *UK* The United Kingdom, *N.A*. Not Available

The smallest number of sample is 42 by Simons-Morton et al. who divided a small group of teenagers into two clusters by their risks of being involved in risky driving [17]. The largest study has 492,306 individuals who participated in NIH–AARP Diet and Health Study [18]. In terms of the location of the studies, majority were conducted in North America and Europe with US (*n* = 109) and UK (*n* = 27) having the most number of the studies.

### Segmentation variables, data sources and settings

Additional file 4 summarized variables used for segmentation analysis in the included studies. They are heterogeneous depending on the segmentation objectives. To illustrate, Keel et al. segmented a population of patients with eating disorders using their symptom variables (e.g. self-induced vomiting) in order to empirically define eating disorder phenotypes [19]. Some used behavioral variables (e.g. tobacco use) to identify meaningful patterns of health related behaviors [20–23]. Environment features (e.g. public park density) were used by some authors to examine patterns of environment features and explore whether the environmental patterns explained health related behaviors and indicators (e.g. physical activity and body mass index) in the neighborhood [24–27]. Vuik et al. retrieved health service utilization data (e.g. number of non-elective inpatient admissions) from administrative databases to segment a general patient population into homogenous groups with distinct healthcare utilization patterns [6]. Many studies used self-reported dietary intake variables (e.g. fiber intake) to derive dietary patterns [28–34]

As for the sources of data, as shown in Table 2, majority (*n* = 170) utilized secondary data originally collected for other research purposes or by someone other than the user (e.g. censuses, administrative databases, other studies with open datasets). One study by Fukuoka et al. retrieved 12-month follow-up data of a randomized clinical trial to identify patient subgroups based on cardiac symptoms after cardiac events [35]. As an another example, Héroux et al. utilized a subset of data on health behaviors from a prospective, observational study to observe the clustering of unhealthy diet, fitness, smoking, and excessive alcohol consumption in adults [36]. 48 studies collected primary data for the purpose of segmentation. To illustrate, researchers in a study conducted clinical interviews and administered questionnaires on post-traumatic stress disorder (PTSD) symptoms in order to identify subtypes of PTSD by segmentation analysis [37]. Another study collected data by telephone interviews of a sample of adults [38].

**Table 2:** Features of data used for data-driven population segmentation

Data source	No. of studies	Examples
Primary	46	Conducting clinical interviews and administering questionnaires [37]
Secondary	168	12-month follow-up data from a randomized clinical trial [35]
Both	2	
Settings		
Healthcare institutions	31	Hospitals [41]
Community	181	Primary schools [40]
Both	4	

Most study settings are in the community (*n* = 185). Gjelsvik et al. studied a sample of women who were recruited in a national survey by landline phone numbers [39]. Another study was based on secondary data from a school-based health intervention programs [40]. Some studies were conducted in healthcare institutions. For example, Penrod et al. examined hip fracture patients recruited from five hospitals in the US [41].

### Objectives of segmentation

The recurring themes of population segmentation objectives in the included studies are: 1) Resource Allocation, 2) Health /Prognostic Index, 3) Health Grouping / Profiling, and 4) Delivery of healthcare interventions (Table 3). These themes are overlapping and not mutually exclusive. Studies that looked into Resource Allocation (*n* = 12) focused on population’s overall medical utilization patterns, trends, and expenditures. Those that aimed at Health/Prognostic Index (*n* = 17) generated health states that represented a person’s risk profile (e.g. for inpatient admission days and mortality). Consistent with overarching theme of population segmentation, all included articles (*n* = 216) focused on Health Grouping / Profiling of their targeted population. Finally, many studies also aimed at Delivery of healthcare interventions (*n* = 50) that are specific and tailored for each population segment. An example for each them is provided in Table 4. Many studies addressed more than one of the above themes as seen in (Additional file 4).

**Table 3:** Objectives of segmentation

Objective (themes)	No. of studies	Examples
Resource Allocation	12	Patients were grouped into segments with distinct care utilization, based on six utilization variables: non-elective inpatient admissions, elective inpatient admissions, outpatient visits, GP practice visits, GP home visits, and prescriptions, creating eight distinct care user types [6].
Health /Prognostic Index	17	Patients were divided into groups that will have similar risk of atrial fibrillation after coronary artery bypass graft, facilitating informed decision making regarding aggressive prophylaxis of atrial fibrillation [72].
Health Grouping / Profiling	216	Individuals were divided into groups based on their dietary patterns: ‘traditional fish eaters’, ‘healthy eaters’, ‘average, less fish, less healthy’, ‘Western’, ‘traditional bread eaters’, and ‘alcohol users’ [73].
Delivery of Healthcare Interventions	50	Participants in the Wellington Respiratory Survey were divided based on five distinct clinical phenotypes of airflow obstruction which may form the basis of a modified taxonomy for the disorders of airways obstruction and treatment specifically targeted at defined phenotypic groups, rather than asthma or COPD in general, which represents the current management approach [74].

Abbreviations: *COPD* Chronic Obstructive Pulmonary Diseases

**Table 4:** Commonly used data-driven population segmentation methods

Methods#	No. of studies	Advantages	Disadvantages	Notes
Unsupervised Classifications				
Latent class/profile/transition/growth analysis	96	1. Can handle missing data [75] 2. Availability of goodness-of-fit measures to assess model fit and determine the appropriate number of segments (e.g. Akaike Information Criterion, Bayesian Information Criterion, standardized entropy) [57–59] 3. No need to standardize variables [76]	Can be computationally intensive, especially with datasets that contain thousands of observations [76]	1. Segmenting variables need to be categorical, continuous, and categorical at multiple time points for latent class analysis, latent profile analysis, and latent transition analysis respectively [77] 2. Users need to pre-specify the desired number of segments
K-means cluster analysis	60	1. Can deal with very large datasets [45, 78] 2. Able to handle both continuous and categorical properties [79, 80]	1. Might not guarantee reproducible solutions (may get a different solution for each set of specified seed points) [81] 2. Sensitive to outliers [82, 83] 3. Limited statistical assistance in determining the optimal number of clusters [76]	Users need to pre-specify the desired number of segments.
Hierarchical analysis	50	1. Stopping rules are readily available (e.g. Duda’s pseudo T square statistic, and Calinski’s pseudo F statistic) to determine ideal cluster solutions [70, 84–86] 2. Dendogram provided offer a simple and comprehensive visual presentation of segmentation solutions [87] 3. Can handle variables of different kinds, (e.g., continuous, binary, nominal)	1. Difficult to handle large datasets (sample size is preferably under 300–400, not exceeding 1000) [88] 2. Sensitive to outliers [82, 83]	
Supervised Classification				
Decision Tree Methods (CHAID/CART)	10	1. Can handle outliers and missing data [89] 2. Computationally fast [90]	Models are based on splits that depend on previous splits; an error made in a higher split will propagate down [90]	Users need to pre-specify dependent (or target) variables

Abbreviations: *CHAID* Chi-square Automatic Interaction Detector, *CART* Classification and Regression Tree

# Some studies applied multiple methods in tandem or in combination

### Segmentation methods

As seen in Table 4, the commonly used statistical techniques are broadly divided into: 1) unsupervised classification whereby all independent (predictor or explanatory) variables are simultaneously considered, and there is no *a-prior* dependent (target) variables; and 2) supervised classification for which users need to pre-specify dependent, or target variables [42] To illustrate, Hearty et al. derived dietary patterns in an adult population using dietary intake as input segmenting variables by K-means cluster analysis which simultaneously considered all input variables to generate 6 dietary patterns (hence interdependent segmentation) [43]. On the other hand, Leclerc et al. performed a dependent segmentation - classification and regression tree analysis (CART) - which recursively splits a group of elderly into two subgroups between which a pre-specified dependent variable (incidence of recurrent faller) is distinct and independent variables (history of falls in the past 3 months, Berg balance score, type of housing, alcohol consumption in the past 6 months etc.) are homogeneous [44]. Unsupervised classification is further categorized into: 1) algorithmic methods which assigns cluster membership to an individual using distance-based approaches (e.g. K-means and hierarchical analysis) and 2) parametric methods (e.g, latent class analysis and its extensions such as latent profile analysis) which assigns an individual to a cluster with, for example, the maximum posterior probability of membership.

The most widely used technique is latent class/profile/transition/growth analysis (*n* = 96) followed by K-means cluster analysis (*n* = 60) and hierarchical analysis (*n* = 50). The segmentation methods were not mutually exclusive; some used combined approaches (e.g. hierarchical cluster analysis first to determine the optimal number of clusters followed by K means clustering analysis). Some, but not all included studies explicitly explained the choice of a specific segmentation methods over the others. For instance, Croezen et al. explained K-means cluster analysis is the more suitable method when there are a large number of subjects as in their study [45]. The major advantages and disadvantages of commonly used statistical methods (used by more than 10 studies included in this systematic review) are listed in Table 3.

### Segmentation outcome

The segmentation outcome of each study was assessed and summarized in (Additional file 5). The following criteria adapted from consumer market segmentation were used: *internal validity* (the fit between the cluster structure and data was assessed by data themselves. For example, one can split a sample into two random subsamples to both of which a cluster analysis is applied independently and the agreement between the two cluster solutions is then assessed [46])*, external validity* (the performance of segmentation was measured by matching a cluster structure to exogenous information). For example, segmentation analysis of dietary intake was validated by demonstrating that individuals in different dietary pattern clusters had significant different body mass index and serum total cholesterol level [47]), *identifiability/interpretability* (segments should be recognized and interpreted easily), *substantiality* (each segment should have sufficient size), *stability* (each segment should be relatively stable over time), *actionability*/a*ccessibility* (each segment should be easily addressed and targeted with distinctive heath intervention strategies) [48, 49]. As summarized in Table 5, most studies fulfilled *internal validity, identifiability/interpretability, substantiality,* and *actionability*/a*ccessibility.* 138 studies have assessed the external validity of the segmentation outcome by variables other than segmenting variables. For instance, Freeman et al. segmented patients with sleep disordered breathing in early childhood by symptoms (e.g. snoring) and validated the segmentation outcome by demonstrating that the risks of tonsillectomies and wheezing frequency differed significantly across the derived segments [50]. Another study divided a population of adolescents using their physical activity and sedentary behavioral variables by segmentation analysis, which was validated by its discriminative ability for the likelihood of meeting national activity recommendations later in adulthood in each segment [51]. However, very few studies (*n* = 10) assessed *stability.* We also included *parsimony* as an additional criterion as the number of segments should be reasonably small to facilitate policy planning and facilitate practical adoption of a segmentation framework. Most studies derived less than 10 segments. For example, Griffin et al. used data on health related behaviors (e.g. exercise, smoking, alcohol consumption, diet and cancer screening behaviors) to cluster a cohort into 6 groups: “smokers”, “non-screeners”, “higher risk ex-smokers” (did not exercise at recommended levels and consumed with alcohol consumption above the sample mean and fruit and vegetable intake below the mean) “lower risk ex-smokers” (engaged in recommended levels of exercise, undertook cancer screening, and consumed above average amounts of fruit and vegetables), “sedentary non-smokers” (did not exercise, had average fruit and vegetable intake, engaged in cancer screening, and consumed the lowest amount of alcohol), and “active non-smokers” (engaged in recommended levels of exercise, undertook cancer screening, consumed more fruit and vegetables than other groups and relatively less alcohol.) [52]. Newby et al. segmented a sample of population into 5 clusters based on data on dietary intake: “healthy”, “white-bread”, “alcohol”, “sweets”, and “meat-and-potatoes” patterns [53].

**Table 5:** Segmentation outcome evaluations

Number of segments (parsimony)	No. of studies	Examples
<=3	76	A population of PTSD patients was segmented based on symptoms: “High-Symptom”, “Dysphoric”, and “Threat” [91].
4–5	98	A group of children was divided into clusters of different patterns of sun protective behaviors: “Multiple protective behaviors”, “Clothing and shade”, “Pants only”, and “Low/inconsistent protective behaviors” [40].
6–9	55	An adult population was segmented by dietary patterns: “Traditional Irish”, “Continental”, “Unhealthy foods”, “Light-meal foods & low-fat milk”, “Healthy foods”, and “Wholemeal bread & dessert” [43].
> = 10	4	A female population was divided into 43 groups based on mammography status, access to care, health behaviors (e.g. smoking), health status etc. ^44^
Internal validation		
Yes	216	The optimal number of clusters was assessed using the Bayesian Information Criterion [92]
No	0	
External validation		
Yes	138	Using risks of tonsillectomies and wheezing frequency to validate segmentation analysis based on symptoms of sleep disordered breathing [50]
No	78	
Identifiability/Interpretability		
Yes	216	Segmentation analysis of dietary patterns derived clusters that are easily identified as “Alcohol cluster”, “Meat cluster”, “Healthy cluster”, and “Refined sugars cluster” [47]
No	0	
Substantiality		
Yes	216	The smallest segment of a clustering analysis of asthma symptoms is composed of 15.8% of the population [93]
No	0	
Stability		
Yes	10	A segmentation analysis of a asthma patient population with 10-year follow up showed the segments remain relatively stable 10 years apart (probability of cluster membership in the same asthma cluster at both times varied between 54 to 88%) [94]
No	206	
Actionability/Accessibility		
Yes	216	A population is divided into segments with distinct sun protection behavioral patterns, for each of which future sun protection interventions tailored to specific subgroups can be designed and delivered to achieve meaningful behavioral changes [40]
No	0	

## Discussion

Data-driven segmentation analysis is widely used in healthcare research. The 216 original research papers included in this systematic review covered various disciplines including respiratory medicine, psychiatry, gastroenterology, dietetics, oncology, cardiology, and public health. It was applied in various populations and clinical settings with difference population characteristics across the globe. The variables used for segmentation analysis vary substantially, depending on the availability of data, the objective of segmentation, and how researchers intend to measure individuals in the study sample. Clustering solutions depend on input variables. Therefore, researchers need to be particularly cognizant of the variables to be used for segmentation purposes, which requires clinical experience, contextual knowledge, conceptual support, adjustment and iterations [54]. It was also observed that data used for segmentation can come from various sources, with some from primary data collection by questionnaires or interviews and others using secondary data from large randomized clinical trials, cohort studies, and administrative databases. This further adds to the potential of segmentation analysis as more EHRs become increasingly visible.

There are a large number of statistical techniques and software packages available for data-driven population segmentation analysis. Each technique is different and has specific properties, which may lead different interpretations of the underlying structure of the data [55]. Different segmentation solutions may be derived even given the same set of input variables [56]. Explaining in technical terms on how each method works is beyond the scope of this review. Researchers should consider each methods’ assumptions and requirement, unique advantages and disadvantages when conducting segmentation analysis. For example, latent class analysis has goodness-of-fit measures available to help users determine model fit and the statistically optimal number of segments (e.g. Akaike Information Criterion, Bayesian Information criterion, standardized entropy, and bootstrapped likelihood ratio test) but can only accept categorical data [57–59]. The choice between the techniques (or combinations of different methods, if desired) depends on the data properties, sample size, research questions, aims of research and the expertise of researchers [60].

After segments are derived by segmentation analysis, the next step is for researchers to assess the quality of the segmentation outcome [61]. Like any other data analysis, segmentation analysis is an iterative process with many potential variations, including input data for segmentation, statistical techniques employed, different number of clusters and profiles [62]. The criteria for optimal segmentation outcome of healthcare data are not well-established [56]. In the field of consumer market segmentation, the following criteria were proposed to assess the segmentation effectiveness: *internal validity, external validity, identifiability/interpretability*, *substantiality*, *stability*, and *actionability*/a*ccessibility* [48, 49]. In this systematic review, only 10 studies evaluated *stability.* In general, individuals in a population need to have stable segment membership over time to allow for long-term healthcare interventions and policy making. It is thus important to have longitudinal studies to assess the *stability* of a segmentation framework. Another challenge is to interpret and name the derived segment. They involve subjective examination to identify characteristics within each cluster and distinguish substantial differences between clusters. For example, a “healthy” segment in one study on dietary patterns was named because this segment has high consumption in vegetables, fruits, fish, whole grains, and low-fat dairy while in another study, a segment characterized by high dietary intake of brown bread, low-fat spreads, low-fat milk, and fruit was given the same label “healthy” [63–65]. Therefore, the characteristics of each cluster and the differences between clusters should be carefully evaluated with theoretical expertise or clinical experience [66]. Thus, to assess the segmentation outcome requires a combination of statistical reasoning, clinical judgment, policy implications, and many other quantitative and qualitative criteria. While the above criteria from market segmentation seem to be relevant to population segmentation in the context of healthcare, it is imperative to develop a conceptual framework with comprehensive criteria for evaluation of segmentation outcome specific to healthcare studies.

This study is the first systematic review of data-driven population segmentation analysis. We summarized the commonly used segmentation methods, the evaluations of segmentation outcomes, and various clinical settings to which the segmentation analysis was applied, including both adult and pediatric population, general population and those with specific diseases or conditions. It is also the first to compare and contrast the strengths, limitations, and practical considerations for commonly used segmentation methods to guide future research that used data-driven population segmentation analysis. This study also provides directions on how to assess the segmentation results. Nonetheless, our study is limited by excluding non-English literature.

## Conclusions

Data-driven population segmentation holds great potential in managing population health and has been widely applied in various clinical contexts. Many segmentation analysis methods are available to derive population clusters. The evaluations of segmentation outcome require statistical criteria and clinical experience. The optimal framework for assessment of segmentation results require further research.

## Additional files

Additional file 1: Search terms in the PubMed® Topic Specific Query “Population Health” category. This file includes the search terms used in the PubMed® Topic Specific Query “Population Health” category (DOCX 118 kb)

Additional file 2: Top Journals. This file includes top 50 journals in public health and top 3 journals in population health according to impact factors in 2016 by SCImago Scientific Journal Rankings and InCites Journal Citation Reports (DOCX 111 kb)

Additional file 3: **Table S1.** The population of interest in studies included in this systematic review. This file includes the features of target population in studies included in this systematic review as well their population size, country/region, data sources, and study settings. (DOCX 186 kb)

Additional file 4: **Table S2.** Segmentation details in studies included for this systematic review. This file includes the details of the segmentation results in studies included in this systematic review, including the objectives of segmentation, segmentation variables, statistical methods used, software, number and names of segments. (DOCX 229 kb)

Additional file 5: **Table S3.** Evaluation of segmentation outcome in studies included for this systematic review. This files includes the detailed assessment of segmentation outcome in studies included for this systematic review, including their internal validation, external validation and validation variables used, identifiability/interpretability, substantiality, stability, and actionability/accessibility (DOCX 160 kb)

## Abbreviations

AIDS: Acquired immune deficiency syndrome
CART: Classification and regression tree
CHAID: Chi-square automatic interaction detector
COPD: Chronic obstructive pulmonary diseases
EHRs: Electronic health records
HIV: Human immunodeficiency virus
N.A.: Not available
PRISMA: Preferred reporting items for systematic reviews and meta-analyses
PTSD: Post-traumatic stress disorder
UK: The United Kingdom
US: The United States of America
