# Supplementary material for: A systematic review of the clinical application of data-driven population segmentation analysis
Source: BMC Med Res Methodol. 2018 Nov 3;18:121. doi: 10.1186/s12874-018-0584-9 (PMC6215625; doi:10.1186/s12874-018-0584-9)
Supplement: Supplementary file 1 — Search terms in the PubMed® Topic Specific Query “Population Health” category. This file includes the search terms used in the PubMed® Topic Specific Query “Population Health” category (DOCX 118 kb) [file 12874_2018_584_MOESM1_ESM.docx]

Search terms in the PubMed® Topic Specific Query “Population Health” category

(typolog*[Title/Abstract] OR stratif*[Title/Abstract] OR segment*[Title/Abstract] OR categor*[Title/Abstract] OR “cluster analysis”[MeSH] OR cluster*[Title/Abstract] OR pattern*[Title/Abstract] OR profil*[Title/Abstract] OR phenotyp*[Title/Abstract] OR class*[Title/Abstract] OR partition*[Title/Abstract]) AND ((population health[tiab] AND ("public health"[MeSH Major Topic] OR "public health administration"[MeSH Terms] OR "public health/education"[Mesh Terms] OR "public health practice"[MeSH Terms] OR "public policy"[MeSH Terms])) OR (population[tiab] AND "health care quality, access, and evaluation"[MeSH Major Topic] AND "demography"[MeSH Major Topic] AND "health services"[MeSH Major Topic]) OR ("health status indicators"[MeSH Major Topic] AND "population characteristics"[MeSH Major Topic] AND "social environment"[MeSH Major Topic]) OR ("health services accessibility"[MeSH Terms] AND "demography"[MeSH Terms] AND disparities[tiab]) OR ((population health[tiab] OR "Built environment"[tiab]) AND ("Urban health"[mesh] OR "Urban health"[tiab] OR "rural health"[mh] OR "rural health"[tiab] OR "urban population"[mh] OR "rural population"[mh] OR "population density"[mh] OR "immigrant health"[tiab] OR "Emigration and Immigration"[Mesh] OR ((livable[tiab] OR liveable[tiab]) AND (neighborhoods[tiab] OR neighborhood[tiab])))) OR (population health[tiab] AND ("health care quality, access, and evaluation"[MeSH Terms] OR "preventive health services"[MeSH Terms] OR "health planning"[MeSH Terms] OR "health policy"[MeSH Terms] OR "demography"[MeSH Terms] OR "health expenditures"[MeSH Terms] OR "income"[MeSH Terms] OR "population dynamics"[MeSH Terms] OR "social determinants of health"[MeSH Terms] OR "socioeconomic factors"[MeSH Terms] OR "population characteristics"[MeSH Terms] OR "health promotion"[MeSH Terms] OR "public health/methods"[Mesh Terms])) OR (population health[tiab] AND ("community health services"[MeSH Major Topic] OR "health services"[MeSH Major Topic] OR "health services research"[MeSH Major Topic] OR "delivery of health care"[MeSH Major Topic] OR "health planning"[MeSH Major Topic] OR "health policy"[MeSH Major Topic] OR "preventive health services"[MeSH Major Topic] OR "health care evaluation mechanisms"[MeSH Major Topic] OR "public health administration"[MeSH Major Topic] OR "public policy"[MeSH Major Topic] OR "quality of life"[MeSH Major Topic] OR "health surveys"[MeSH Major Topic] OR "public health administration"[MeSH Major Topic] OR "demography"[MeSH Major Topic] OR "socioeconomic factors"[MeSH Major Topic] OR "health behavior"[MeSH Major Topic] OR "attitude to health"[MeSH Major Topic] OR "social environment"[MeSH Major Topic] OR "social welfare"[MeSH Major Topic] OR "population characteristics"[MeSH Major Topic] OR "vulnerable populations"[MeSH Major Topic] OR "residence characteristics"[MeSH Terms] OR "poverty"[MeSH Major Topic] OR "healthcare disparities"[MeSH Major Topic])) OR (("health status indicators"[MeSH Terms] OR "health status"[mesh]) AND "mass screening/methods"[MAJR] AND "morbidity"[MeSH Major Topic]) OR ("health status indicators"[MeSH Major Topic] AND "public health practice"[MeSH Major Topic] AND "outcome assessment (health care)"[MeSH Terms]) OR (("built environment"[tiab] OR "housing"[mh]) AND ("public health"[MeSH Major Topic] OR "population"[MeSH Terms] OR "population health"[tiab] OR "population groups"[MeSH Terms] OR "population groups"[tiab]) AND (socioeconomic[tiab] OR inequality[tiab] OR inequalities[tiab] OR disparity[tiab] OR disparities[tiab] OR equity[tiab] OR inequity[tiab] OR inequities[tiab] OR policy[tiab] OR policies[tiab] OR determinants[tiab])) OR ("Built environment"[tiab] AND ("public health"[MeSH Major Topic] OR "population health"[tiab] OR "population groups"[MeSH Terms] OR "population groups"[tiab]) AND ("food supply"[mesh] OR "food insecurity"[tiab]) OR "food desert"[tiab] OR "food deserts"[tiab] OR "food environments"[tiab] OR "food environment"[tiab] OR "food swamps"[tiab]) OR (("Built environment"[tiab] OR "population health"[tiab]) AND ("physical activity"[tiab] OR "exercise"[mesh] OR "sedentary lifestyle"[mesh]) AND ("motor activity"[mesh] OR Walkability[tiab] OR walkable[tiab] OR "active transportation"[tiab] OR "bike share"[tiab] OR "active commuting"[tiab] OR "daily steps"[tiab] OR ((livable[tiab] OR liveable[tiab]) AND (neighborhoods[tiab] OR neighborhood[tiab])))))
