# Supplementary material for: A systematic review of the clinical application of data-driven population segmentation analysis
Source: BMC Med Res Methodol. 2018 Nov 3;18:121. doi: 10.1186/s12874-018-0584-9 (PMC6215625; doi:10.1186/s12874-018-0584-9)
Supplement: Supplementary file 2 — Top Journals. This file includes top 50 journals in public health and top 3 journals in population health according to impact factors in 2016 by SCImago Scientific Journal Rankings and InCites Journal Citation Reports (DOCX 111 kb) [file 12874_2018_584_MOESM2_ESM.docx]

Top Journals

American journal of preventive medicine, Vital and health statistics. Series 10, Data from the National Health Survey, Annual review of public health, Environmental health perspectives, Obesity reviews : an official journal of the International Association for the Study of Obesity, National health statistics reports, Tobacco control, Bulletin of the World Health Organization, Environmental research letters : ERL, Translational research : the journal of laboratory and clinical medicine, International journal of hygiene and environmental health, Ecotoxicology and environmental safety, The Journal of adolescent health : official publication of the Society for Adolescent Medicine, Euro surveillance : bulletin Européen sur les maladies transmissibles = European communicable disease bulletin, PLoS neglected tropical diseases, Indoor air, Journal of cancer epidemiology, Scandinavian journal of work, environment & health, PharmacoEconomics, Nicotine & tobacco research : official journal of the Society for Research on Nicotine and Tobacco, Preventive medicine, Epidemics, Occupational and environmental medicine, Vaccine, International journal of chronic obstructive pulmonary disease, Journal of occupational health psychology, American journal of public health, Journal of the International AIDS Society, Implementation science : IS, Reviews of environmental contamination and toxicology, AIDS patient care and STDs, Journal of epidemiology and community health, International journal of health geographics, Suicide & life-threatening behavior, Pediatric obesity, Clinical microbiology reviews, "Environmental health : a global access science source, Primary care respiratory journal : journal of the General Practice Airways Group, Burnout research, Population health metrics, Accident; analysis and prevention, Value in health : the journal of the International Society for Pharmacoeconomics and Outcomes Research, Vascular health and risk management, Influenza and other respiratory viruses, Medical care, Journal of health and social behavior, Tropical medicine & international health : TM & IH, NPJ primary care respiratory medicine, The Milbank quarterly, Population health management, The Lancet. Global health, Clinical epidemiology, Journal of global health
