# Supplementary material for: A systematic review of the clinical application of data-driven population segmentation analysis
Source: BMC Med Res Methodol. 2018 Nov 3;18:121. doi: 10.1186/s12874-018-0584-9 (PMC6215625; doi:10.1186/s12874-018-0584-9)
Supplement: Supplementary file 3 — Table S1. The population of interest in studies included in this systematic review. This file includes the features of target population in studies included in this systematic review as well their population size, country/region, data sources, and study settings. (DOCX 186 kb) [file 12874_2018_584_MOESM3_ESM.docx]

**Table S1. The population of interest in studies included in this systematic review**

| **Studies** | **Author** | **Target population** | **Population Size** | **Country/**  **Region** | **Data sources#** | | **Study settings** | |
| --- | --- | --- | --- | --- | --- | --- | --- | --- |
| A quantitative evidence base for population health: applying utilization- based cluster analysis to segment a patient population | Vuik et al. (2016) | General patient population | 300,000 | UK | • Secondary data  • Administrative healthcare database | | Healthcare Institutions | |
|  |  |  |  |  |  | |  | |
| A Typology of Predictive Risk Factors for Non- Adherent Medication-Related Behaviors among Chronic Non-Cancer Pain Patients Prescribed Opioids: A Cohort Study | Peacock et al. (2016) | Chronic pain patients | 1,514 | Australia | • Primary data  • Prospective cohort study  • Interviews and questionnaires | | Community | |
|  |  |  |  |  |  | |  | |
| Classification and Regression Tree Uncovered Hierarchy of Psychosocial Determinants Underlying Quality Of Life Response Shift in HIV/AIDS | Li et al. (2009) | HIV positive patients | 394 | US | • Secondary data  • Cohort study | | Community | |
|  |  |  |  |  |  | |  | |
| Clustering of adolescent health concerns: A latent class analysis of school students in New Zealand | Noel et al. (2016) | Secondary school and Alternative education students | 9,442 | New Zealand | • Primary data  • Cross-sectional study  • Questionnaires | | Community | |
|  |  |  |  |  |  | |  | |
| Continuum of Mammography Use among US Women: Classification Tree Analysis | Gjelsvik et al. (2014) | General female adult population | 169,427 | US | • Secondary data  • Cohort study | | Community | |
|  |  |  |  |  |  | |  | |
| Exploring Statistical Approaches to Diminish Subjectivity of Cluster Analysis to Derive Dietary Patterns-The Tomorrow Project | Siou et al. (2011) | General adult population | 16,744 | Canada | • Secondary data  • Cohort study | | Community | |
|  |  |  |  |  |  | |  | |
| Identifying adult asthma phenotypes using a clustering approach | Siroux et al. (2011) | Asthma patients (adult) | 1,895 | Europe | • Secondary data  • Cohort study | | Community | |
|  |  | Asthma patients (adult and children) | 641 | France | • Secondary data  • Case-control study | | Community | |
|  |  |  |  |  |  | |  | |
| Latent typologies of posttraumatic stress disorder in World Trade Center responders | Horn et al. (2011) | 9/11 World Trade Centre (WTC) responders | 4,352 | US | • Primary data  • Cross-sectional study  • Interviews and questionnaire | | Community | |
|  |  |  |  |  |  | |  | |
| Signiﬁcance of Symptom Clustering in Palliative Care of Advanced Cancer Patients | Tsai et al. (2010) | Cancer patients | 427 | Taiwan | • Primary data  • Cross-sectional study  • Questionnaire | | Healthcare Institutions | |
|  |  |  |  |  |  | |  | |
| Snoring, mouth-breathing, and apnea trajectories in a population-based cohort followed from infancy to 81 months: A cluster analysis | Freeman et al. (2012) | Sleep disordered breathing children | 10,441 | UK | • Secondary data  • Cohort study | | Community | |
|  |  |  |  |  |  | |  | |
| Ten-Year Follow-up of Cluster-based Asthma Phenotypes in Adults | Boudier et al. (2013) | Asthma patients (adult) | 3,320 | Europe | • Secondary data  • Two cohort studies and one case control study | | Community | |
|  |  |  |  |  |  | |  | |
| The clustering of health behaviors in Ireland and their relationship with mental health, self- rated health and quality of life | Conry et al. (2011) | General adult population | 7,350 | Ireland | • Secondary data  • Cross-sectional study | | Community | |
|  |  |  |  |  |  | |  | |
| Who does not reduce their sodium intake despite being advised to do so? A population segmentation analysis | Li et al. (2017) | General adult population | 125,764 | US | • Secondary data  • Cohort study | | Community | |
|  |  |  |  |  |  | |  | |
| A Classification and Regression Tree for Predicting Recurrent Falling among Community- dwelling Seniors Using Home-care Services | Leclerc et al. (2009) | General elderly population | 868 | Canada | • Primary data  • Cohort study  • Questionnaire | | Community | |
|  |  |  |  |  |  | |  | |
| Adults' Physical Activity Patterns across Life Domains: Cluster Analysis with Replication | Rovniak et al. (2010) | General adult population | 1,689 | US | • Secondary data  • Cohort study | | Community | |
|  |  |  |  |  |  | |  | |
| Collaborative evaluation and management of students’ health-related physical ﬁtness: applications of cluster analysis and the classiﬁcation tree | Chen et al. (2012) | General youth population | 742 | Taiwan | • Primary data  • Cross-sectional study  • Fitness tests and clinical assessment | | Community | |
|  |  |  |  |  |  | |  | |
| GIS-measured walkability, transit, and recreation environments in relation to older Adults' physical activity: A latent proﬁle analysis | Todd et al. (2016) | General elderly population | 714 | US | • Secondary data  • Cohort study | | Community | |
|  |  |  |  |  |  | |  | |
| Hierarchical cluster analysis of labor market regulations and population health: a taxonomy of low- and middle-income countries | Muntaner et al. (2012) | General population | N.A. | International | • Secondary data  • Databases at International Labor Organization and World Health Organization | | Community | |
|  |  |  |  |  |  | |  | |
| Identifying Unique Neighborhood Characteristics to Guide Health Planning for Stroke and Heart Attack: Fuzzy Cluster and Discriminant Analyses Approaches | Pedigo et al. (2011) | General population | N.A. | US | • Secondary data  • Databases at US Census and Tennessee Department of Health | | Community | |
|  |  |  |  |  |  | |  | |
| K-Means Cluster Analysis of Rehabilitation Service Users in the Home Health Care System of Ontario: Examining the Heterogeneity of a Complex Geriatric Population | Armstrong et al. (2012) | General elderly population | 150,253 | Canada | • Secondary data  • Administrative healthcare database | | Community | |
|  |  |  |  |  |  | |  | |
| Neighborhood socioeconomic status and food environment: A 20-year longitudinal latent class analysis among CARDIA participants | Richardson et al. (2014) | General adult population | 5,114 | US | • Secondary data  • Cohort study and US Census | | Community | |
|  |  |  |  |  |  | |  | |
| Neighborhood typology based on virtual audit of environmental obesogenic characteristics | Feuillet et al. (2015) | General population | N.A. | Europe | • Secondary data  • Google Street View and European Environment Agency | | Community | |
|  |  |  |  |  |  | |  | |
| Reducing consumption of confectionery foods: A post-hoc segmentation analysis using a social cognition approach | Naughton et al. (2017) | General adult population | 500 | Ireland | • Primary data  • Cross-sectional study  • Questionnaire | | Community | |
|  |  |  |  |  |  | |  | |
| Understanding the social patterning of smoking practices: a dynamic typology | Narcisse et al. (2009) | General population | 9,896 | Canada | • Secondary data  • Cohort study | | Community | |
|  |  |  |  |  |  | |  | |
| “Do you see what I see?” – Correlates of multidimensional measures of neighborhood types and perceived physical activity–related neighborhood barriers and facilitators for urban youth | Yan et al. (2010) | General youth population | 350 | US | • Primary data  • Cross-sectional study  • Questionnaire | | Community | |
|  |  |  |  |  |  | |  | |
| A latent class analysis of cancer risk behaviors among U.S. college students | Kang et al. (2014) | General youth population | 30,093 | US | • Secondary data  • Cross-sectional study | | Community | |
|  |  |  |  |  |  | |  | |
| A park typology in the QUALITY cohort: Implications for physical activity and truncal fat among youth at risk of obesity | Bird et al. (2016) | Children at risk of obesity | 380 | Canada | • Secondary data  • Cohort study | | Community | |
|  |  |  |  |  |  | |  | |
| Adolescent Physical Activity and Sedentary Behavior - Patterning and Long-Term Maintenance | Nelson et al. (2005) | General youth population | 11,957 | US | • Secondary data  • Cohort study | | Community | |
|  |  |  |  |  |  | |  | |
| Anger types and the use of cigarettes and smokeless tobacco among Native American adolescents | Kerby et al. (2003) | General youth population | 513 | US | • Secondary data  • Cross-sectional study | | Community | |
|  |  |  |  |  |  | |  | |
| Clusters of lifestyle behaviors: Results from the Dutch SMILE study | Vries et al. (2008) | General patient population | 9,449 | The Netherlands | • Secondary data  • Cohort study | | Community | |
|  |  |  |  |  |  | |  | |
| Identifying Heterogeneity Among Injection Drug Users: A Cluster Analysis Approach | Shaw et al. (2008) | Injection drug users | 435 | Canada | • Secondary data  • Cross-sectional study | | Community | |
|  |  |  |  |  |  | |  | |
| Lifestyle risk factors of students: A cluster analytical approach | Dodd et al. (2010) | General youth population | 410 | UK | • Primary data  • Cross-sectional study  • Questionnaire | | Community | |
|  |  |  |  |  |  | |  | |
| Longitudinal Patterns of Health Insurance Coverage Among a National Sample of Children in the Child Welfare System | Raghavan et al. (2008) | General children population | 2,501 | US | • Secondary data  • Cohort study | | Community | |
|  |  |  |  |  |  | |  | |
| Modiﬁable lifestyle behavior patterns, sedentary time and physical activity contexts: A cluster analysis among middle school boys and girls in the SALTA study | Marques et al. (2013) | General youth population | 636 | Portugal | • Secondary data  • Cohort study | | Community | |
|  |  |  |  |  |  | |  | |
| Neighborhood environment proﬁles related to physical activity and weight status: A latent proﬁle analysis | Adams et al. (2013) | General population | 2,199 | US | • Secondary data  • Cohort study | | Community | |
|  |  |  |  |  |  | |  | |
| Patterns of Obesogenic Neighborhood Features and Adolescent Weight -  A Comparison of Statistical Approaches | Wall et al. (2012) | Middle school and high school students | 2,682 | US | • Secondary data  • Cross-sectional study | | Community | |
|  |  |  |  |  |  | |  | |
| Patterns of Physical Activity Among Older Adults in New York City - A Latent Class Approach | Mooney et al. (2015) | General elderly population | 3,497 | US | • Secondary data  • Cross-sectional study | | Community | |
|  |  |  |  |  |  | |  | |
| Patterns of sun protective behaviors among Hispanic children in a skin cancer prevention intervention | Miller et al. (2015) | Primary school students | 972 | US | • Primary data  • Cross-sectional study  • Questionnaire | | Community | |
|  |  |  |  |  |  | |  | |
| Patterns of Visit Attendance in the Nurse –Family Partnership Program | Holland et al. (2014) | Pregnant women | 228 | US | • Secondary data  • Randomized controlled trial | | Community | |
|  |  |  |  |  |  | |  | |
| Patterns of Walkability, Transit, and Recreation Environment for Physical Activity | Adams et al. (2015) | General population | 2,199 | US | • Secondary data  • Cohort study | | Community | |
|  |  |  |  |  |  | |  | |
| The clustering of health-related behaviors in a British population sample: Testing for cohort differences | Mawditt et al. (2016) | General population | 21,019 | UK | • Secondary data  • Cross-sectional study | | Community | |
|  |  |  |  |  |  | |  | |
| A Latent Class Analysis of Dissociation and PTSD: Evidence for a Dissociative Subtype | Wolf et al. (2012) | Veterans and their spouses/intimate partners | 492 | US | • Primary data  • Cross-sectional study  • Clinical interviews and questionnaire | | Healthcare Institutions | |
|  |  |  |  |  |  | |  | |
| A Latent Profile Analysis of Neighborhood Recreation Environments in Relation to Adolescent Physical Activity, Sedentary Time, and Obesity | Norman et al. (2010) | General youth population | 871 | US | • Primary and secondary data  • Cross-sectional study  • Clinical assessment and US Census | | Community | |
|  |  |  |  |  |  | |  | |
| A typology of neighborhoods and blood pressure in the RECORD Cohort Study | Hulst et al. (2012) | General adult population | 7,290 | France | • Secondary data  • Cohort study | | Community | |
|  |  |  |  |  |  | |  | |
| An Investigation of Activity Profiles of Older Adults | Morrow-Howell et al. (2014) | General elderly population | 4,593 | US | • Secondary data  • Cohort study | | Community | |
|  |  |  |  |  |  | |  | |
| Applying Recursive Partitioning to a Prospective Study of Factors Associated with Adherence to Mammography Screening Guidelines | Calvocoressi et al. (2005) | General female adult population | 1,229 | US | • Primary and secondary data  • Cohort study  • Telephone interviews | | Community | |
|  |  |  |  |  |  | |  | |
| Associations between food patterns defined by cluster analysis and colorectal cancer incidence in the NIH–AARP diet and health study | Wirfalt et al. (2009) | General adult population | 492,306 | US | • Secondary data  • Cohort study | | Community | |
|  |  |  |  |  |  | |  | |
| Associations of empirically derived eating patterns with plasma lipid biomarkers: a comparison of factor and cluster analysis methods | Newby et al. (2004) | General adult population | 459 | US | • Secondary data  • Cross-sectional study | | Community | |
|  |  |  |  |  |  | |  | |
| Built and Social Environments - Associations with Adolescent Overweight and Activity | Nelson et al. (2006) | General youth population | 20,745 | US | • Secondary data  • Cohort study | | Community | |
|  |  |  |  |  |  | |  | |
| Capturing changes in dietary patterns among older adults: a latent class analysis of an ageing Irish cohort | Harrington et al. (2014) | General adult population | 923 | Ireland | • Secondary data  • Cohort study | | Community | |
|  |  |  |  |  |  | |  | |
| Characterizing Longitudinal Patterns of Physical Activity in Mid-Adulthood Using Latent Class Analysis: Results From a Prospective Cohort Study | Silverwood et al. (2011) | General adult population | 3,847 | UK | • Secondary data  • Cohort study | | Community | |
|  |  |  |  |  |  | |  | |
| Cluster Analysis and Clinical Asthma Phenotypes | Haldar et al. (2008) | Asthma patients | 184 | UK | • Secondary data  • Two cohort studies | | Healthcare Institutions | |
|  |  | Refractory asthma patients | 187 | UK | • Primary data  • cross-sectional study  • Clinical assessment | Healthcare Institutions | |  |
|  |  | Refractory asthma patients | 68 | UK | • Secondary data  • Randomized controlled trial | | Healthcare Institutions | |
|  |  |  |  |  |  | |  | |
| Cluster Analysis of Elderly Cardiac Patients’ Prehospital Symptomatology | Lindgren et al. (2008) | Elderly ischemic coronary heart disease patients | 247 | US | • Secondary data  • Randomized controlled trial | | Healthcare Institutions | |
|  |  |  |  |  |  | |  | |
| Cluster analysis of symptoms and health seeking behavior differentiates subgroups of patients with severe irritable bowel syndrome | Guthrie et al. (2003) | IBS patients | 107 | UK | • Secondary data  • Randomized controlled trial | | Healthcare Institutions | |
|  |  |  |  |  |  | |  | |
| Cluster analysis: a useful technique to identify elderly cardiac patients at risk for poor quality of life | Fukuoka et al. (2003) | Elderly ischemic coronary heart disease patients | 206 | US | • Secondary data  • Randomized controlled trial | | Healthcare Institutions | |
|  |  |  |  |  |  | |  | |
| Clustering of cardiovascular risk factors in Australian adolescents: association with dietary excesses and deficiencies | Milligan et al. (1995) | General youth population | 555 | Australia | • Secondary data  • Cohort study | | Community | |
|  |  |  |  |  |  | |  | |
| Clustering of health behaviors in adult survivors of childhood cancer and the general population | Rebholz et al. (2012) | Cancer patients | 2,505 | Switzerland | • Secondary data  • Two cohort studies | | Community | |
|  |  |  |  |  |  | |  | |
| Clustering of health risk behaviors and the relationship with mental disorders | Vermeulen-Smit et al. (2015) | General population | 5,303 | The Netherlands | • Secondary data  • Cohort study | | Community | |
|  |  |  |  |  |  | |  | |
| Clustering of lifestyle risk behaviors among residents of forty deprived neighborhoods in London: lessons for targeting public health interventions | Watts et al. (2015) | General adult population | 5,321 | UK | • Secondary data  • Cluster randomized trial | | Community | |
|  |  |  |  |  |  | |  | |
| Clustering of modifiable biobehavioral risk factors for chronic disease in US adults: a latent class analysis | Leventhal et al. (2014) | General adult population | 22,789 | US | • Secondary data  • Cohort study | | Community | |
|  |  |  |  |  |  | |  | |
| Clustering of Unhealthy Behaviors in the Aerobics Center Longitudinal Study | Héroux et al. (2012) | General adult population | 13,621 | US | • Secondary data  • Cohort study | | Community | |
|  |  |  |  |  |  | |  | |
| Clustering Women’s Health Behaviors | Hagoel et al. (2002) | General female adult population | 1,075 | Israel | • Secondary data  • Cohort study | | Community | |
|  |  |  |  |  |  | |  | |
| Comparative Strategies for Using Cluster Analysis to Assess Dietary Patterns | Bailey et al. (2006) | General elderly population | 179 | US | • Secondary data  • Cohort study | | Community | |
|  |  |  |  |  |  | |  | |
| Comparing 3 Dietary Pattern Methods—Cluster Analysis, Factor Analysis, and Index Analysis—With Colorectal Cancer Risk | Reedy et al. (2009) | General adult population | 492,306 | US | • Secondary data  • Cohort study | | Community | |
|  |  |  |  |  |  | |  | |
| Comparison of cluster and principal component analysis techniques to derive dietary patterns in Irish adults | Hearty et al. (2009) | General adult population | 1,379 | Ireland | • Secondary data  • Cross-sectional study | | Community | |
|  |  |  |  |  |  | |  | |
| Developing an empirical typology for regular exercise | Norman et al. (2003) | General adult population | 346 | US | • Primary data  • Cross-sectional study  • Telephone interview | | Community | |
|  |  |  |  |  |  | |  | |
| Dietary patterns among a national random sample of British adults | Pryer et al. (2001) | General adult population | 2,197 | UK | • Secondary data  • Cross-sectional study | | Community | |
|  |  |  |  |  |  | |  | |
| Dietary patterns among older Europeans: the EPIC-Elderly study | Bamia et al. (2005) | General elderly population | 99,744 | Europe | • Secondary data  • Cross-sectional study | | Community | |
|  |  |  |  |  |  | |  | |
| Dietary patterns and changes in body mass index and waist circumference in adults | Newby et al. (2003) | General adult population | 459 | US | • Secondary data  • Cross-sectional study | | Community | |
|  |  |  |  |  |  | |  | |
| Distinguishing phenotypes of childhood wheeze and cough using latent class analysis | Spycher et al. (2008) | Preschool children | 319 | UK | • Secondary data  • Cohort study | | Community | |
|  |  |  |  |  |  | |  | |
| Drug use patterns and adherence to treatment among HIV-positive patients: evidence from a large sample of French outpatients (ANRS-EN12-VESPA 2003) | Peretti-Watel et al. (2006) | HIV positive patients | 4,963 | France | • Secondary data  • Cross-sectional study | | Healthcare institutions | |
|  |  |  |  |  |  | |  | |
| Food patterns and cardiovascular disease risk factors: The Swedish INTERGENE research program | Berg et al. (2008) | General adult population | 3,452 | Sweden | • Secondary data  • Cohort study | | Community | |
|  |  |  |  |  |  | |  | |
| Food patterns deﬁned by cluster analysis and their utility as dietary exposure variables: a report from the Malmo Diet and Cancer Study | Wirfalt et al. (1999) | General adult population | 5,357 | Sweden | • Secondary data  • Cohort study | | Community | |
|  |  |  |  |  |  | |  | |
| Health Lifestyles: Audience Segmentation Analysis for Public Health Interventions | Slater et al. (1991) | General adult population | 2,502 | US | • Secondary data  • Cohort study | | Community | |
|  |  |  |  |  |  | |  | |
| Health State Profiles and Service Utilization in Community-Living Elderly | Lafortune et al. (2009) | Frail elderly population | 1,164 | Canada | • Secondary data  • Randomized controlled trial | | Community | |
|  |  |  |  |  |  | |  | |
| Heterogeneity in Hip Fracture Patients: Age, Functional Status, and Comorbidity | Penrod et al. (2007) | Hip fracture patients | 2,692 | US | • Secondary data  • Cohort study | | Healthcare Institutions | |
|  |  |  |  |  |  | |  | |
| Heterogeneity of severe asthma in childhood: Conﬁrmation by cluster analysis of children in the National Institutes of Health/National Heart, Lung, and Blood Institute Severe Asthma Research Program | Fitzpatrick et al. (2011) | Asthma patients (Children) | 161 | US | • Secondary data  • Cohort study | | Healthcare Institutions | |
|  |  |  |  |  |  | |  | |
| Identification of asthma clusters in two independent Korean adult asthma cohorts | Kim et al. (2013) | Asthma patients | 2,567 | Korea | • Secondary data  • Two cohort studies | | Healthcare Institutions | |
|  |  |  |  |  |  | |  | |
| Identiﬁcation of Asthma Phenotypes Using Cluster Analysis in the Severe Asthma Research Program | Moore et al. (2010) | Asthma patients | 726 | US | • Secondary data  • Cohort study | | Healthcare Institutions | |
|  |  |  |  |  |  | |  | |
| Identifying built environmental patterns using cluster analysis and GIS: Relationships with walking, cycling and body mass index in French adults | Charreire et al. (2012) | General adult population | 1,309 | France | • Secondary data  • Cohort study, French census | | Community | |
|  |  |  |  |  |  | |  | |
| Identifying mobility heterogeneity in very frail older adults. Are frail people all the same? | Montero-Odasso et al. (2009) | Frail elderly population | 1,160 | France | • Secondary data  • Cohort study | | Community | |
|  |  |  |  |  |  | |  | |
| Identifying Patterns of Eating and Physical activity in children: A Latent class analysis of Obesity Risk | Huh et al. (2010) | Primary school students | 997 | US | • Secondary data  • Randomized controlled trial | | Community | |
|  |  |  |  |  |  | |  | |
| Identifying risk profiles for childhood obesity using recursive partitioning based on individual, familial, and neighborhood environment factors | Hulst et al. (2015) | General youth population | 512 | Canada | • Secondary data  • Cohort study | | Community | |
|  |  |  |  |  |  | |  | |
| Is depression associated with health risk-related behavior clusters in adults? | Verger et al. (2009) | General adult population | 17,355 | France | • Secondary data  • Cross-sectional study | | Community | |
|  |  |  |  |  |  | |  | |
| Latent class analysis applied to health behaviors | Ingledew et al. (1995) | General adult population | 109 | UK | • Primary data  • Cross-sectional study  • Questionnaires | | Healthcare Institutions | |
|  |  |  |  |  |  | |  | |
| Latent Class Analysis of Lifestyle Characteristics and Health Risk Behaviors among College Youth | Laska et al. (2009) | General youth population | 2,026 | US | • Secondary data  • Cohort study | | Community | |
|  |  |  |  |  |  | |  | |
| Latent Transition Analysis: Benefits of a Latent Variable Approach to Modeling Transitions in Substance Use | Lanza et al. (2010) | General youth population | 718 | US | • Secondary data  • Cohort study | | Community | |
|  |  |  |  |  |  | |  | |
| Neighborhood Environment Profiles for Physical Activity Among Older Adults | Adams et al. (2012) | General elderly population | 728 | US | • Secondary data  • Cohort study | | Community | |
|  |  |  |  |  |  | |  | |
| Obesogenic clusters: multidimensional adolescent obesity- related behaviors in the U.S. | Boone-Heinonen et al. (2008) | General youth population | 9,251 | US | • Secondary data  • Cohort study | | Community | |
|  |  |  |  |  |  | |  | |
| Patterns of health risk behaviors among job-seekers: a latent class analysis | Schnuerer et al. (2015) | General adult population | 7,905 | Germany | • Secondary data  • Randomized controlled trial | | Community | |
|  |  |  |  |  |  | |  | |
| Patterns of neighborhood environment attributes related to physical activity across 11 countries: a latent class analysis | Adams et al. (2013) | General adult population | 11,541 | International | • Secondary data  • Cross-sectional study | | Community | |
|  |  |  |  |  |  | |  | |
| Patterns of Physical Activity, Sedentary Behavior, and Diet in U.S. Adolescents | Iannotti and Wang (2013) | General youth population | 9,174 | US | • Primary data  • Cross-sectional study  • Questionnaires | | Community | |
|  |  |  |  |  |  | |  | |
| Patterns of Substance Use in Early Through Late Adolescence | Zapert, Snow, and Tebes (2002) | General youth population | 764 | US | • Secondary data  • Cohort study | | Community | |
|  |  |  |  |  |  | |  | |
| Physical activity and sedentary activity patterns among children and adolescents: a latent class analysis approach | Heitzler et al. (2011) | General youth population | 720 | US | • Secondary data  • Cohort study | | Community | |
|  |  |  |  |  |  | |  | |
| Physical activity and sedentary behavior typologies of 10-11 year olds | Jago et al. (2010) | General youth population | 761 | UK | • Secondary data  • Cross-sectional study | | Community | |
|  |  |  |  |  |  | |  | |
| Recursive partitioning–based preoperative risk stratification for atrial fibrillation after coronary artery bypass surgery | Sedrakyan et al. (2006) | Post-CABG Patients | 1,209 | US | • Primary data  • Cohort study  • Clinical assessment | | Healthcare Institutions | |
|  |  |  |  |  |  | |  | |
| Risk behavior, parental background, and wealth: A cluster analysis among Swedish boys and girls in the HBSC study | Carlerby et al. (2012) | General youth population | 11,972 | Sweden | • Secondary data  • Cross-sectional study | | Community | |
|  |  |  |  |  |  | |  | |
| Socioeconomic differences in dietary patterns among middle-aged men and women | Martikainen et al. (2003) | General adult population | 8,004 | UK | • Secondary data  • Cross-sectional study | | Community | |
|  |  |  |  |  |  | |  | |
| Symptom clustering in advanced cancer | Walsh and Rybicki (2006) | Cancer patients | 922 | US | • Primary data  • Cross-sectional study  • Questionnaire | | Healthcare Institutions | |
|  |  |  |  |  |  | |  | |
| Symptom Clusters and Relationships to Symptom Interference with Daily Life in Taiwanese Lung Cancer Patients | Wang et al. (2008) | Cancer patients | 108 | Taiwan | • Primary data  • Cross-sectional study  • Questionnaire | | Healthcare Institutions | |
|  |  |  |  |  |  | |  | |
| The effect of symptom clusters on functional status and quality of life in women with breast cancer | Dodd et al. (2010) | Cancer patients | 112 | US | • Secondary data  • Cohort study | | Healthcare Institutions | |
|  |  |  |  |  |  | |  | |
| The influence of health behavior clusters on dietary change | Reedy et al. (2005) | Cancer patients | 595 | US | • Secondary data  • Randomized controlled trial | | Community | |
|  |  |  |  |  |  | |  | |
| The internal validity of a dietary pattern analysis. The Framingham Nutrition Studies | Quatromoni et al. (2001) | General female population | 1,942 | US | • Secondary data  • Cohort study | | Community | |
|  |  |  |  |  |  | |  | |
| The Structure of Posttraumatic Stress Disorder | Breslau et al. (2005) | Adults at risk of PTSD | 3,276 | US | • Secondary data  • Two cross-sectional studies | | Community | |
|  |  |  |  |  |  | |  | |
| Tobacco, Marijuana, and Alcohol Use in University Students: A Cluster Analysis | Primack et al. (2012) | General youth population | 111,245 | US | • Secondary data  • Cross-sectional study | | Community | |
|  |  |  |  |  |  | |  | |
| Toward an Empirical Taxonomy of Suicide Ideation: A Cluster Analysis of the Youth Risk Behavior Survey | Flannery et al. (2003) | General youth population | 2,730 | US | • Primary data  • Cross-sectional study  • Questionnaire | | Community | |
|  |  |  |  |  |  | |  | |
| Tracing the Mediterranean diet through principal components and cluster analyses in the Greek population | Costacou et al. (2003) | General adult population | 28,034 | Greece | • Secondary data  • Cohort study | | Community | |
|  |  |  |  |  |  | |  | |
| Trajectories of posttraumatic stress symptomatology in older persons affected by a large-magnitude disaster | Pietrzak et al. (2013) | Elderly at risk of PTSD | 206 | US | • Primary data  • Cohort study  • Telephone interview | | Community | |
|  |  |  |  |  |  | |  | |
| Transition to College: Α Classification and Regression Tree (CART) analysis of natural reduction of binge drinking | Vik et al. (2006) | General youth population | 201 | US | • Primary data  • Cross-sectional study  • Questionnaire | | Community | |
|  |  |  |  |  |  | |  | |
| Transitions in drug use among high-risk women: an application of latent class and latent transition analysis | Lanza and Bray (2010) | HIV-positive women | 457 | US | • Secondary data  • Cohort study | | Community | |
|  |  |  |  |  |  | |  | |
| Types of alcoholics, I - evidence for an Empirically Derived Typology Based on Indicators of Vulnerability and Severity | Babor et al. (1992) | Adult alcoholics | 321 | US | • Primary data  • Cohort study  • Clinical interviews, laboratory tests, and questionnaires | | Healthcare Institutions | |
|  |  |  |  |  |  | |  | |
| Typologies of posttraumatic stress disorder in the U.S. adult population | Pietrzak et al. (2014) | PTSD patients | 2,463 | US | • Secondary data  • Cohort study | | Community | |
|  |  |  |  |  |  | |  | |
| Typologies of posttraumatic stress disorder in treatment-seeking older adults | Böttche et al. (2015) | Elderly PTSD patients | 164 | Germany | • Primary data  • Cross-sectional study  • Questionnaires | | Community | |
|  |  |  |  |  |  | |  | |
| A latent class analysis of illicit drug abuse/dependence: results from the National Epidemiological Survey on Alcohol and Related Conditions | Agrawal et al. (2006) | General population | 43,093 | US | • Secondary data  • Cohort study | | Community | |
|  |  |  |  |  |  | |  | |
| A latent class analysis of underage problem drinking: Evidence from a community sample of 16–20 year olds | Reboussin et al. (2006) | Youth drinkers | 4,056 | US | • Secondary data  • Cross-sectional study | | Community | |
|  |  |  |  |  |  | |  | |
| A longitudinal investigation of the impact of typology of urinary incontinence on quality of life during midlife: Results from a British prospective study | Mishra et al. (2009) | General female adult population | 983 | UK | • Secondary data  • Cohort study | | Community | |
|  |  |  |  |  |  | |  | |
| A Longitudinal Typology of Symptoms of Depression and Anxiety Over the Life Course | Colman et al. (2007) | General population | 4,627 | UK | • Secondary data  • Cohort study | | Community | |
|  |  |  |  |  |  | |  | |
| An Empirical Study of the Classification of Eating Disorders | Bulik, Sullivan, and Kendler (2000) | General female population | 2,163 | US | • Secondary data  • Cohort study | | Community | |
|  |  |  |  |  |  | |  | |
| Application of a Latent Class Analysis to Empirically Define Eating Disorder Phenotypes | Keel et al. (2004) | Eating disorder patients | 1,179 | International | • Secondary data  • Cross-sectional study | | Community and healthcare Institutions | |
|  |  |  |  |  |  | |  | |
| Bone mineral density and dietary patterns in older adults: the Framingham Osteoporosis Study | Tucker et al. (2002) | General elderly population | 907 | US | • Secondary data  • Cohort study | | Community | |
|  |  |  |  |  |  | |  | |
| Characterization of different groups of elderly according to social engagement activity patterns | Croezen et al. (2009) | General elderly population | 22,026 | The Netherlands | • Secondary data  • Cross-sectional study | | Community | |
|  |  |  |  |  |  | |  | |
| Classes of disruptive behavior in a sample of young elementary school children | Lier et al. (2003) | Primary school students | 636 | The Netherlands | • Secondary data  • Cohort study | | Community | |
|  |  |  |  |  |  | |  | |
| Classification of suicide attempters by cluster analysis: a study of the temperamental heterogeneity in suicidal patients | Engstrom et al. (1996) | Suicide attempters | 215 | Sweden | • Primary data  • Cross-sectional study  • Questionnaires | | Healthcare institutions | |
|  |  |  |  |  |  | |  | |
| Classification of Suicide Attempters by Cluster Analysis | Paykel et al. (1978) | Suicide attempters | 236 | UK | • Secondary data  • Cross-sectional study | | Community | |
|  |  |  |  |  |  | |  | |
| Cluster Analysis Methods Help to Clarify the Activity–BMI Relationship of Chinese Youth | Monda and Popkin (2005) | General youth population | 5,631 | China | • Secondary data  • Cohort study | | Community | |
|  |  |  |  |  |  | |  | |
| Clustering of dietary variables and other lifestyle factors (Dutch Nutritional Surveillance System) | Huishof et al. (1992) | General adult population | 3,781 | The Netherlands | • Secondary data  • Cross-sectional study | | Community | |
|  |  |  |  |  |  | |  | |
| Creating Neighborhood Typologies of GIS-Based Data in the Absence of Neighborhood-Based Sampling: A Factor and Cluster Analytic Strategy | Gershoff, Pedersen, and Aber (2009) | General youth population | 908 | US | • Secondary data  • Cohort study | | Community | |
|  |  |  |  |  |  | |  | |
| Developmental Typology of Trajectories to Nighttime Bladder Control: Epidemiologic Application of Longitudinal Latent Class Analysis | Croudace et al. (2002) | General children population | 3,272 | UK | • Secondary data  • Cohort study | | Community | |
|  |  |  |  |  |  | |  | |
| Dietary patterns and adenocarcinoma of the esophagus and distal stomach | Chen et al. (2002) | Cancer patients | 697 | US | • Primary data and secondary data  • Case-control study and administrative database  • Telephone interviews | | Community | |
|  |  |  |  |  |  | |  | |
| Dietary Patterns and Cardiovascular Risk Factors in Elderly Men: The Zutphen Elderly Study | Huijbregts et al. (1995) | General elderly population | 518 | US | • Secondary data  • Cohort study | | Community | |
|  |  |  |  |  |  | |  | |
| Dietary patterns and lifestyle factors in the Norwegian EPIC cohort: The Norwegian Women and Cancer (NOWAC) study | Engeset et al. (2005) | General female adult population | 37,226 | Norway | • Secondary data  • Cross-sectional study | | Community | |
|  |  |  |  |  |  | |  | |
| Dietary Patterns and Nutrient Intakes of 7-Year-Old Children Taking Part in an Atherosclerosis Prevention Project in Finland | Rasanen et al. (2002) | General children population | 630 | Finland | • Secondary data  • Randomized control trial | | Community | |
|  |  |  |  |  |  | |  | |
| Dietary Patterns and Survival of Older Adults | Anderson et al. (2010) | General elderly population | 2,582 | US | • Secondary data  • Cohort study | | Community | |
|  |  |  |  |  |  | |  | |
| Dietary patterns and the adenoma- carcinoma sequence of colorectal cancer | Rouillier et al. (2005) | Patients with colorectal growth | 1,372 | France | • Secondary data  • Case-control study | | Community | |
|  |  |  |  |  |  | |  | |
| Dietary patterns are associated with lower incidence of type 2 diabetes in middle-aged women: the Shanghai Women’s Health Study | Villegas et al. (2010) | General female population | 64,191 | China | • Secondary data  • Cohort study | | Community | |
|  |  |  |  |  |  | |  | |
| Dietary Patterns Associated with Risk for Metabolic Syndrome in Urban Community of Karachi Defined by Cluster Analysis | Hydrie et al. (2010) | General adult population | 871 | Pakistan | • Primary data  • Cross-sectional study  • Questionnaire and clinical assessment | | Community | |
|  |  |  |  |  |  | |  | |
| Dietary patterns in middle-aged Irish men and women deﬁned by cluster analysis | Villegas et al. (2004) | General adult population | 1,018 | Ireland | • Secondary data  • Cross-sectional study | | Community | |
|  |  |  |  |  |  | |  | |
| Dietary patterns in the Southampton Women’s Survey | Crozier et al. (2006) | General female adult population | 6,125 | UK | • Secondary data  • Cross-sectional study | | Community | |
|  |  |  |  |  |  | |  | |
| Dietary patterns of elderly Boston­area residents defined by cluster analysis | Tucker et al. (1992) | General elderly population | 680 | US | • Secondary data  • Cross-sectional study | | Community | |
|  |  |  |  |  |  | |  | |
| Dietary Patterns of Hispanic Elders Are Associated with Acculturation and Obesity | Lin, Bermudez, and Tucker (2013) | General elderly population | 825 | US | • Secondary data  • Cross-sectional study | | Community | |
|  |  |  |  |  |  | |  | |
| Dietary patterns of men and women suggest targets for health promotion- the Framingham Nutrition Studies | Millen et al. (1996) | General adult population | 3,659 | US | • Secondary data  • Cross-sectional study | | Community | |
|  |  |  |  |  |  | |  | |
| Dietary Patterns of Rural Older Adults Are Associated with Weight and Nutritional Status | Ledikwe et al. (2004) | General elderly population | 179 | US | • Secondary data  • Cross-sectional study | | Community | |
|  |  |  |  |  |  | |  | |
| Dietary patterns predict the development of overweight in women- The Framingham nutrition studies. | Quatromoni et al. (2002) | General female adult population | 737 | US | • Secondary data  • Cohort study | | Community | |
|  |  |  |  |  |  | |  | |
| Disentangling women’s responses on complex dietary intake patterns from an Indian cross-sectional survey: a latent class analysis | Padmadas, Dias, and Willekens (2006) | General female population | 90,180 | India | • Secondary data  • Cross-sectional study | | Community | |
|  |  |  |  |  |  | |  | |
| Distinct clinical phenotypes of airways disease defined by cluster analysis | Weatherall et al. (2009) | Respiratory disease patients | 175 | New Zealand | • Secondary data  • Cross-sectional study | | Community | |
|  |  |  |  |  |  | |  | |
| Empirically derived symptom sub-groups correspond poorly with diagnostic criteria for functional dyspepsia and irritable bowel syndrome. A factor and cluster analysis of a patient sample | Eslick et al. (2003) | Patients with gastrointestinal symptoms | 897 | Australia | • Primary data  • Cross-sectional study  • Questionnaire and clinical assessment | | Healthcare institutions | |
|  |  |  |  |  |  | |  | |
| Gastrointestinal Symptoms and Subjects Cluster Into Distinct Upper and Lower Groupings in the Community: A Four Nations Study | Talley et al. (2000) | General adult population | 5,372 | International | • Primary data  • Cross-sectional study  • Questionnaire and clinical assessment | | Community | |
|  |  |  |  |  |  | |  | |
| Identification and Prediction of Latent Classes of Weight-loss Strategies Among Women | Lanza, Savage, and Birch (2010) | General female adult population | 197 | US | • Secondary data  • Cohort study | | Community | |
|  |  |  |  |  |  | |  | |
| Identification of groups who report similar patterns of diet among a representative national sample of British adults aged 65 years of age or more | Pryer, Cook, and Shetty (2000) | General elderly population | 1,097 | UK | • Secondary data  • Cross-sectional study | | Community | |
|  |  |  |  |  |  | |  | |
| Identifying dietary patterns using a normal mixture model: application to the EPIC study | Fahey et al. (2011) | General female adult population | 12,018 | Europe | • Secondary data  • Cohort study | | Community | |
|  |  |  |  |  |  | |  | |
| Identifying target segments of male drinkers for health promotion | Wyllie and Casswell (1993) | Male drinkers | 723 | New Zealand | • Secondary data  • Cross-sectional study | | Community | |
|  |  |  |  |  |  | |  | |
| Latent Class Analysis Is Useful to Classify Pregnant Women into Dietary Patterns | Sotres-Alvarez, Herring, and Siega-Riz (2010) | Pregnant women | 1,352 | US | • Secondary data  • Cohort study | | Healthcare institutions | |
|  |  |  |  |  |  | |  | |
| Latent Class Analysis of Lifetime Depressive Symptoms in the National Comorbidity Survey | Sullivan, Kessler, and Kendler (1998) | General population | 2,836 | US | • Secondary data  • Cohort study | | Community | |
|  |  |  |  |  |  | |  | |
| Latent Transition Models to Study Women’s Changing of Dietary Patterns From Pregnancy to 1 Year Postpartum | Sotres-Alvarez, Herring, and Siega-Riz (2013) | Pregnant and postpartum women | 424 | US | • Secondary data  • Cohort study | | Healthcare institutions | |
|  |  |  |  |  |  | |  | |
| Leisure-time physical activity and sedentary behavior clusters and their associations with overweight in middle-aged French adults | Charreire et al. (2010) | General adult population | 4,682 | France | • Secondary data  • Randomized controlled trial | | Community | |
|  |  |  |  |  |  | |  | |
| Patterns of health-related behavior and their cross-cultural validity - A comparative study on two populations of young people | Karvonen et al. (2000) | General youth population | 552 | Finland and Switzerland | • Secondary data  • Two cross-sectional studies | | Community | |
|  |  |  |  |  |  | |  | |
| Relationships of dietary patterns with body composition in older adults differ by gender and PPAR-γ Pro12Ala genotype | Anderson et al. (2010) | General elderly population | 1,809 | US | • Secondary data  • Cohort study | | Community | |
|  |  |  |  |  |  | |  | |
| Resilience and patterns of health risk behaviors in California adolescents | Mistry et al. (2009) | General youth population | 4,010 | US | • Secondary data  • Cross-sectional study | | Community | |
|  |  |  |  |  |  | |  | |
| Seven unique food consumption patterns identiﬁed among women in the UK Women’s Cohort Study | Greenwood et al. (2000) | General female adult population | 33,971 | UK | • Secondary data  • Cohort study | | Community | |
|  |  |  |  |  |  | |  | |
| Subgroups of Patients With Cancer With Different Symptom Experiences and Quality-of-Life Outcomes: A Cluster Analysis | Miaskowski et al. (2006) | Cancer patients | 191 | US | • Primary data  • Cross-sectional study  • Questionnaires | | Community | |
|  |  |  |  |  |  | |  | |
| The Structure of Psychosis - Latent Class Analysis of Probands From the Roscommon Family Study | Kendler et al. (1998) | Patients with schizophrenia and affective illness | 343 | Ireland | • Primary data  • Cross-sectional study  • Household interviews | | Community | |
|  |  |  |  |  |  | |  | |
| Using Cluster Analysis to Examine Dietary Patterns- Nutrient Intakes, Gender, and Weight Status Differ Across Food Pattern Clusters | Wirfalt and Jeffery (1997) | General adult population | 522 | US | • Secondary data  • Cross-sectional study | | Community | |
|  |  |  |  |  |  | |  | |
| A Latent Class Analysis of Stigmatizing Attitudes and Knowledge of HIV Risk among Youth in South Africa | Brinkley-Rubinstein et al. (2014) | General youth population | 1,347 | South Africa | • Secondary data  • Cohort study | | Community | |
|  |  |  |  |  |  | |  | |
| A person-centred segmentation study in elderly care: Towards efﬁcient demand-driven care | Laan et al. (2014) | General elderly population | 2,019 | The Netherlands | • Secondary data  • Cross-sectional study | | Community | |
|  |  |  |  |  |  | |  | |
| Health status transitions in community-living elderly with complex care needs: a latent class approach | Lafortune et al. (2009) | Frail elderly population | 1,164 | Canada | • Secondary data  • Randomized controlled trial | | Community | |
|  |  |  |  |  |  | |  | |
| The Clustering of Health Behaviours in Older Australians and its Association with Physical and Psychological Status, and Sociodemographic Indicators | Griffin  et al. (2014) | General adult population | 96,276 | Australia | • Secondary data  • Cohort study | | Community | |
|  |  |  |  |  |  | |  | |
| The heterogeneous health latent classes of elderly people and their socio-demographic characteristics in Taiwan | Liu et al. (2014) | General elderly population | 2,449 | Taiwan | • Secondary data  • Cohort study | | Community | |
|  |  |  |  |  |  | |  | |
| Utilization of health care services by elderly people with National Health Insurance in Taiwan: The heterogeneous health proﬁle approach | Liu et al. (2012) | General elderly population | 1,491 | Taiwan | • Secondary data  • Cohort study | | Community | |
|  |  |  |  |  |  | |  | |
| A Cluster Analysis of Physical Activity and Sedentary Behavior Patterns in Middle School Girls | Trilk et al. (2012) | Female youth population | 957 | US | • Secondary data  • Randomized controlled trial | | Community | |
|  |  |  |  |  |  | |  | |
| A hierarchy of sociodemographic and environmental correlates of walking and obesity | Frank et al. (2008) | General population | 13,065 | US | • Secondary data  • Cross-sectional study | | Community | |
|  |  |  |  |  |  | |  | |
| A Latent Class Analysis of DSM-IV and Fagerström (FTND) Criteria for Nicotine Dependence | Agrawal et al. (2011) | Smokers | 624 | US | • Secondary data  • A national registry | | Community | |
|  |  |  |  |  |  | |  | |
| A Latent Class Analysis of Risk Factors for Acquiring HIV Among Men Who Have Sex with Men: Implications for Implementing Pre-Exposure Prophylaxis Programs | Chan et al. (2015) | Men who have sex with men | 449 | US | • Primary data  • Cross-sectional study  • Questionnaires | | Healthcare institutions | |
|  |  |  |  |  |  | |  | |
| A latent class model to identify city/town chronic disease patterns | Jiang et al. (2015) | General population | 39 cities/towns | US | • Secondary data  • Cross-sectional studies and administrative databases | | Community and healthcare institutions | |
|  |  |  |  |  |  | |  | |
| A Latent Transition Model of the Effects of a Teen Dating Violence Prevention Initiative | Williams et al. (2015) | General youth population | 1,517 | US | • Secondary data  • Cohort study | | Community | |
|  |  |  |  |  |  | |  | |
| Adolescent physical activity and the built environment: A latent class analysis approach | McDonald et al. (2012) | General youth population | 344 | US | • Secondary data  • Cohort study | | Community | |
|  |  |  |  |  |  | |  | |
| Comparison of Suicide Attempters and Decedents in the U.S. Army: A Latent Class Analysis | Skopp et al. (2016) | Soldiers who attempted suicide | 1,857 | US | • Secondary data  • Administrative databases | | Community | |
|  |  |  |  |  |  | |  | |
| Complex Comorbidity Clusters in OEF/OIF Veterans - The Polytrauma Clinical Triad and Beyond | Pugh et al. (2014) | Veterans | 191,797 | US | • Secondary data  • Administrative databases | | Community | |
|  |  |  |  |  |  | |  | |
| Coping, Stress, and Social Support Associations With Internalizing and Externalizing Behavior Among Urban Adolescents and Young Adults: Revelations From a Cluster Analysis | Tandon et al. (2013) | General youth population | 683 | US | • Primary data  • Cross-sectional study  • Questionnaires | | Community | |
|  |  |  |  |  |  | |  | |
| Differences in environmental preferences towards cycling for transport among adults: a latent class analysis | Mertens et al. (2016) | General adult population | 1,950 | Belgium | • Primary data  • Cross-sectional study  • Questionnaires | | Community | |
|  |  |  |  |  |  | |  | |
| Distinct symptom experiences in subgroups of patients with COPD | Christensen et al. (2016) | Adult patients with COPD | 267 | Norway | • Primary data  • Cross-sectional study  • Questionnaires | | Community | |
|  |  |  |  |  |  | |  | |
| Effects of clustering of multiple lifestyle-related behaviors on blood pressure in adolescents from two observational studies | Moraes et al. (2016) | General youth population | 1,252 | Europe and Brazil | • Secondary data  • Cross-sectional study | | Community | |
|  |  |  |  |  |  | |  | |
| Exploring the application of latent class cluster analysis for investigating pedestrian crash injury severities in Switzerland | Sasidharan et al. (2015) | General population | N.A. | Switzerland | • Secondary data  • Administrative databases | | Community | |
|  |  |  |  |  |  | |  | |
| Health lifestyle behaviors among U.S. adults | Onge and Krueger (2017) | General adult population | 152,500 | US | • Secondary data  • Cross-sectional study | | Community | |
|  |  |  |  |  |  | |  | |
| Health-Related Fitness Proﬁles in Adolescents With Complex Congenital Heart Disease | Klausen et al. (2015) | Youth with previous surgery for complex congenital heart disease | 158 | Denmark | • Primary data  • Cross-sectional study  • Clinical assessment | | Community | |
|  |  |  |  |  |  | |  | |
| Latent class analysis of acceptability and willingness to pay for self-HIV testing in a United States urban neighborhood with high rates of HIV infection | Nunn et al. (2017) | General adult population | 1,535 | US | • Primary data  • Cross-sectional study  • Questionnaire | | Community | |
|  |  |  |  |  |  | |  | |
| Latent class modelling of the association between socioeconomic background and breast cancer survival status at 5 years incorporating stage of disease | Downing et al. (2010) | Patients with invasive breast cancer | 11,781 | UK | • Secondary data  • Administrative databases | | Healthcare institutions | |
|  |  |  |  |  |  | |  | |
| Latent Classes of Young adults Based on Use of Multiple types of tobacco and nicotine Products | Erickson, Lenk, and Forster (2014) | General youth population | 2,624 | US | • Secondary data  • Cohort study | | Community | |
|  |  |  |  |  |  | |  | |
| Latent Homeless Risk Proﬁles of a National Sample of Homeless Veterans and Their Relation to Program Referral and Admission Patterns | Tsai, Kasprow, and Rosenheck (2013) | Homeless Veterans | 120,852 | US | • Secondary data  • Administrative databases | | Community | |
|  |  |  |  |  |  | |  | |
| Latent variable mixture models to test for differential item functioning: a population- based analysis | Wu et al. (2017) | General adult population | 9,423 | Canada | • Secondary data  • Cohort study | | Community | |
|  |  |  |  |  |  | |  | |
| Pattern Analysis of Suicide Mortality Surveillance Data in Urban South Africa | Burrows and Laflamme (2008) | Suicide attempters | 4,977 | South Africa | • Secondary data  • Administrative databases | | Community | |
|  |  |  |  |  |  | |  | |
| Patterns of Alternative Tobacco Product Use: Emergence of Hookah and E-cigarettes as Preferred Products Amongst Youth | Gilreath et al. (2016) | General youth population | 2,097 | US | • Secondary data  • Cohort study | | Community | |
|  |  |  |  |  |  | |  | |
| Patterns of Hospitalization Risk for Women Surviving Into Very Old Age | Dolja-Gore et al. (2017) | Elderly female population | 1,936 | Australia | • Secondary data  • Cohort study and administrative databases | | Community and healthcare institutions | |
|  |  |  |  |  |  | |  | |
| Patterns of neighborhood environment attributes in relation to children's physical activity | Kurka et al. (2015) | General youth population | 678 | US | • Primary data  • Cross-sectional study  • Questionnaire | | Community | |
|  |  |  |  |  |  | |  | |
| Patterns of Physical Activity, Sedentary Behavior and Diet in US Adolescents | Iannotti and Wang (2013) | General youth population | 9,206 | US | • Primary data  • Cross-sectional study  • Questionnaire | | Community | |
|  |  |  |  |  |  | |  | |
| Patterns of risky behaviors associated with methamphetamine use among young Thai adults: A latent class analysis | Sherman et al. (2009) | Youth methamphetamine smokers | 1,189 | Thailand | • Primary data  • Cross-sectional study  • Questionnaire | | Community | |
|  |  |  |  |  |  | |  | |
| Prevalence and Patterns of Polysubstance Use in a Nationally Representative Sample of 10th Graders in the United States | Conway et al. (2013) | General youth population | 2,524 | US | • Secondary data  • Cohort study | | Community | |
|  |  |  |  |  |  | |  | |
| Smoking Patterns and their relationship to Drinking among First-Year College students | Hoeppner et al. (2014) | General youth population | 266 | US | • Primary data  • Cohort study  • Questionnaire | | Community | |
|  |  |  |  |  |  | |  | |
| Smoking patterns during pregnancy and postnatal period and depressive symptoms | Munafo, Heron, and Araya (2007) | Women during pregnancy and postnatal period | 7,089 | UK | • Secondary data  • Cohort study | | Community | |
|  |  |  |  |  |  | |  | |
| Subgrouping outpatients of an environmental medicine unit using SCL-90-R and cluster analysis | Helm and Eis (2007) | Environmental medicine patients | 169 | Germany | • Primary data  • Cohort study  • Questionnaire | | Healthcare institutions | |
|  |  |  |  |  |  | |  | |
| The Neighbourhood Built Environment and Trajectories of Depression Symptom Episodes in Adults: A Latent Class Growth Analysis | Gariepy et al. (2015) | General adult population | 7,114 | Canada | • Secondary data  • Cross-sectional study | | Community | |
|  |  |  |  |  |  | |  | |
| Time Use and Food Pattern Influences on Obesity | Kolodinsky and Goldstein (2011) | General adult population | 1,451 | US | • Secondary data  • Cross-sectional studies | | Community | |
|  |  |  |  |  |  | |  | |
| Tobacco Use and Suicidality: Latent Patterns of Co-occurrence Among Black Adolescents | Gilreath, Connell, and Leventhal (2012) | General youth population | 2,931 | US | • Secondary data  • Cross-sectional studies | | Community | |
|  |  |  |  |  |  | |  | |
| Trajectories and predictors of return to work after traumatic limb injury – a 2-year follow-up study | Hou et al. (2012) | Patients with traumatic limb injury | 804 | Taiwan | • Primary data  • Cohort study  • Questionnaire | | Healthcare institutions | |
|  |  |  |  |  |  | |  | |
| Trajectories of Cigarette smoking From adolescence to adulthood as Predictors of Unemployment status | Brook et al. (2014) | General population | 806 | US | • Secondary data  • Cohort study | | Community | |
|  |  |  |  |  |  | |  | |
| Trajectories of Depressive Symptoms Among Web-Based Health Risk Assessment Participants | Bedrosian, Hawrilenko, and Cole-Lewis (2017) | General adult population | 22,963 | US | • Secondary data  • Administrative databases | | Community | |
|  |  |  |  |  |  | |  | |
| Trajectories of Depressive Symptoms in Canadian Emerging Adults | Ferro, Gorter, and Boyle (2015) | General youth population | 2,825 | Canada | • Secondary data  • Cohort study | | Community | |
|  |  |  |  |  |  | |  | |
| Trajectories of Kinematic Risky Driving Among Novice Teenagers | Simons-Morton et al. (2013) | General youth population | 42 | US | • Primary data  • Cohort study  • Questionnaire and driving data acquisition system | | Community | |
|  |  |  |  |  |  | |  | |
| Trajectories of Loneliness in Adolescents With Congenital Heart Disease: Associations With Depressive Symptoms and Perceived Health | Vanhalst et al. (2013) | Youth patients with congenital heart disease | 429 | Belgium | • Primary data  • Cohort study  • Questionnaire and administrative databases | | Community and healthcare institutions | |
|  |  |  |  |  |  | |  | |
| Trajectories of physical functioning and their prognostic indicators: A prospective cohort study in older adults with joint pain and comorbidity | Hermsen et al. (2014) | Elderly patients with joint pain and comorbidity | 407 | The Netherlands | • Primary data  • Cohort study  • Questionnaire | | Community | |
|  |  |  |  |  |  | |  | |
| Trajectories of productivity loss over a 20-year period: an analysis of the National Longitudinal Survey of Youth | Besen and Pransky (2014) | General adult population | 5,699 | US | • Secondary data  • Cohort study | | Community | |
|  |  |  |  |  |  | |  | |
| Trajectories of Social Engagement and Limitations in Late Life | Thomas (2011) | General elderly population | 1,667 | US | • Secondary data  • Cohort study | | Community | |
|  |  |  |  |  |  | |  | |
| Trajectories of Suicidal Ideation from Sixth through Tenth Grades in Predicting Suicide Attempts in Young Adulthood in an Urban African American Cohort | Musci et al. (2016) | General youth population | 581 | US | • Secondary data  • Cohort study | | Community | |
|  |  |  |  |  |  | |  | |
| Trajectories or Parental Monitoring and Communication and Effects on Drug Use Among Urban Young Adolescents | Tobler and Komro (2010) | General youth population | 2,621 | US | • Secondary data  • Randomized controlled trial | | Community | |
|  |  |  |  |  |  | |  | |
| Trajectory Classes of Decline in Health-Related Quality of Life in Parkinson’s Disease: A Pilot Study | Klotsche et al. (2011) | Patients with Parkinson’s Disease | 145 | Germany | • Secondary data  • Cohort study | | Healthcare institutions | |
|  |  |  |  |  |  | |  | |
| Transitions in Smokers’ Social Networks After Quit Attempts: A Latent Transition Analysis | Bray et al. (2016) | Smokers | 691 | US | • Secondary data  • Randomized controlled trial | | Healthcare institutions | |
|  |  |  |  |  |  | |  | |
| Transitions in Suicide Risk in a Nationally Representative Sample of Adolescents | Thompson, Kuruwita, and Foster (2009) | General youth population | 10,424 | US | • Secondary data  • Cohort study | | Community | |
|  |  |  |  |  |  | |  | |

**Abbreviations:**  GP: General Practitioner; US: The United States; UK: United Kingdom; GIS: Geographic information systems; PTSD: Post-traumatic stress disorder; BMI: Body mass index; SBP: Systolic blood pressure; DBP: Diastolic blood pressure; IBS: Irritable bowel syndrome; FEV1: Forced expiratory volume during the 1st second; AF: Atrial fibrillation; CABG: Coronary artery bypass grafting; HIV: human immunodeficiency virus; HRQoL: Health related quality of life; BMD: Bone mineral density; DSM: Diagnostic and Statistical Manual of Mental Disorders; COPD: chronic obstructive pulmonary disease; N.A. Not available

# For secondary data, we described data source (e.g. from administrative databases, cohort study etc.); for primary data, we summarized study types (e.g. cross-sectional study) and data collection methods (e.g. questionnaire)
