# Supplementary material for: A systematic review of the clinical application of data-driven population segmentation analysis
Source: BMC Med Res Methodol. 2018 Nov 3;18:121. doi: 10.1186/s12874-018-0584-9 (PMC6215625; doi:10.1186/s12874-018-0584-9)
Supplement: Supplementary file 4 — Table S2. Segmentation details in studies included for this systematic review. This file includes the details of the segmentation results in studies included in this systematic review, including the objectives of segmentation, segmentation variables, statistical methods used, software, number and names of segments. (DOCX 229 kb) [file 12874_2018_584_MOESM4_ESM.docx]

**Table S2. Segmentation details in studies included for this systematic review**

| **Studies** | **Author** | **Objective of Segmentation** | **Segmentation variables** | **Statistical methods** | **Software** | **No. of Segments** | **Names of segments** |
| --- | --- | --- | --- | --- | --- | --- | --- |
| A quantitative evidence base for population health: applying utilization- based cluster analysis to segment a patient population | Vuik et al. (2016) | • Resource allocation  • Health Grouping/Profiling | Care utilization (e.g. non-elective inpatient admissions, elective inpatient admissions, outpatient visits, GP practice visits, GP home visits, and prescriptions) | Hierarchical analysis (Ward's methods) followed by K-means cluster analysis. | Stata 14 SPSS 23 | 8 | “Very low overall care use”, “Low primary care use”, “High emergency care use”, “Specialist care use”, “High primary care use” etc. |
|  |  |  |  |  |  |  |  |
| A Typology of Predictive Risk Factors for Non- Adherent Medication-Related Behaviors among Chronic Non-Cancer Pain Patients Prescribed Opioids: A Cohort Study | Peacock et al. (2016) | • Health Grouping/Profiling  • Delivery of healthcare interventions | Psychosocial (e.g. social support) and clinical (e.g. personal history of drug and/or alcohol misuse, abuse or dependence) | Latent class analysis | Mplus 7 | 4 | “Poor Physical Functioning”, “Poor Coping/Physical Functioning”, “Substance Use Problems”, and “Multiple Comorbid Problems” |
|  |  |  |  |  |  |  |  |
| Classification and Regression Tree Uncovered Hierarchy of Psychosocial Determinants Underlying Quality Of Life Response Shift in HIV/AIDS | Li et al. (2009) | • Health Grouping/Profiling | Dependent variables: HRQoL discrepancy score (difference between observed score and an expected value).  Independent variables: Negative experiences, feelings, concerns, and personal goals (e.g. things that make you feel worried) | Classification and Regression Trees (CART) methods | N.A. | 9 | Nil |
|  |  |  |  |  |  |  |  |
| Clustering of adolescent health concerns: A latent class analysis of school students in New Zealand | Noel et al. (2016) | • Health Grouping/Profiling  • Delivery of healthcare interventions | Behavioural (e.g. unsafe sexual health behaviors), Psychosocial (e.g. depression) | Latent class analysis | Mplus 6.12 | 4 | “Healthy”, “ Risky”, “ Distressed”, and “Multiple” |
|  |  |  |  |  |  |  |  |
| Continuum of Mammography Use among US Women: Classification Tree Analysis | Gjelsvik et al. (2014) | • Resource allocation  • Health Grouping/Profiling  • Delivery of healthcare interventions | Dependent variable: Mammography status.  Independent variable: access to care (e.g insurance status), health behaviors (e.g. smoking), health status (e.g. functional limitation), and socio-demographics (e.g. income) | Exhaustive Chi-Squared Automatic Interaction Detection (E-CHAID) | SPSS Answer  Tree 2.0 | 43 | Nil |
|  |  |  |  |  |  |  |  |
| Exploring Statistical Approaches to Diminish Subjectivity of Cluster Analysis to Derive Dietary Patterns-The Tomorrow Project | Siou et al. (2011) | • Health Grouping/Profiling  • Delivery of healthcare interventions | Dietary intake (percentage of daily total energy intake contributed by the 55 food groups) | Hierarchical analysis (Ward's methods) | SAS 9.1.3 | N.A. | Nil |
|  |  |  |  | K-means cluster analysis | SAS 9.1.3 | 4 for male, 3 for female | “Dairy and sweets”, “ Western”, ‘‘Healthy’’ etc. |
|  |  |  |  | Hierarchical analysis (flexible beta) | SAS 9.1.3 | N.A. | Nil |
|  |  |  |  |  |  |  |  |
| Identifying adult asthma phenotypes using a clustering approach | Siroux et al. (2011) | • Health Grouping/Profiling  • Delivery of healthcare interventions | Disease status (e.g. age of asthma onset and symptoms), laboratory tests (e.g. lung function tests), clinical treatment | Latent class analysis | SAS 9.1 | 4 | “Active treated allergic childhood-onset asthma”, ‘‘Active treated adult-onset asthma’’, ‘‘Inactive /mild untreated allergic asthma’’ etc. |
|  |  |  |  |  |  |  |  |
| Latent typologies of posttraumatic stress disorder in World Trade Center responders | Horn et al. (2011) | • Health Grouping/Profiling | PTSD symptoms (e.g. nightmares) | Latent class analysis | Mplus 7.11 | 3 | “High-Symptom”, “Dysphoric”, and “Threat” |
|  |  |  |  |  |  |  |  |
| Signiﬁcance of Symptom Clustering in Palliative Care of Advanced Cancer Patients | Tsai et al. (2010) | • Health Grouping/Profiling  • Delivery of healthcare interventions | Physical symptoms (e.g. fatigue) | Hierarchical analysis (Ward's methods) followed by K-means cluster analysis | SPSS 11.0 | 6 | “Loss of energy”, ‘‘Poor intake”, “Autonomic dysfunction’’, ‘‘Aerodigestive impairment’’, and ‘‘Pain complex’’ |
|  |  |  |  |  |  |  |  |
| Snoring, mouth-breathing, and apnea trajectories in a population-based cohort followed from infancy to 81 months: A cluster analysis | Freeman et al. (2012) | • Health Grouping/Profiling  • Delivery of healthcare interventions | Symptoms of sleep disordered breathing (e.g. snoring) | K-means cluster analysis | SAS FASTCLUS 9.2 | 5 | ‘‘Normals’’, ‘‘Late snores and mouth-breathing’’, ‘‘Early snores’’, ‘‘Early apnea’’, and ‘‘all SDB after infancy’’ |
|  |  |  |  |  |  |  |  |
| Ten-Year Follow-up of Cluster-based Asthma Phenotypes in Adults | Boudier et al. (2013) | • Health Grouping/Profiling  • Delivery of healthcare interventions | Asthma symptoms, laboratory tests (e.g. lung function tests), clinical treatment | Latent transition analysis | SAS 9.3 | 7 | “Allergic, Few Symptoms, No Treatment”, “Nonallergic, Few Symptoms, No Treatment”, “Nonallergic, High Symptoms, Treatment” etc. |
|  |  |  |  |  |  |  |  |
| The clustering of health behaviors in Ireland and their relationship with mental health, self- rated health and quality of life | Conry et al. (2011) | • Health Grouping/Profiling  • Delivery of healthcare interventions | Health behaviors (e.g. physical activity and diet) | TwoStep Cluster analysis | SPSS 15.0 | 6 | “Former Smokers”, “ Temperate”, “ Physically Inactive”, “ Healthy Lifestyle”, “ Multiple Risk Factor”, and “ Mixed Lifestyle” |
|  |  |  |  |  |  |  |  |
| Who does not reduce their sodium intake despite being advised to do so? A population segmentation analysis | Li et al. (2017) | • Health Grouping/Profiling  • Delivery of healthcare interventions | Dependent variable: self-reported action to reduce sodium intake  Independent variables: Demographics (e.g. age), disease status (e.g. chronic kidney disease), geographic location, BMI, income | Exhaustive Chi-Squared Automatic Interaction Detection (E-CHAID) | SPSS 24 | 26 | Nil |
|  |  |  |  |  |  |  |  |
| A Classification and Regression Tree for Predicting Recurrent Falling among Community- dwelling Seniors Using Home-care Services | Leclerc et al. (2009) | • Health Grouping/Profiling | Dependent variables: Length of time before becoming a recurrent faller  Independent variables: Number of falls in the previous 3 months, nutrition, BMI, gait, medications, alcohol consumptions, number of home environmental hazards, and housing type | Classification and Regression Trees (CART) methods | SPSS 15.0 | 5 | Nil |
|  |  |  |  |  |  |  |  |
| Adults' Physical Activity Patterns across Life Domains: Cluster Analysis with Replication | Rovniak et al. (2010) | • Health Grouping/Profiling | Self-reported moderate- and vigorous-intensity physical activity | Hierarchical analysis (Ward's methods) followed by K-means cluster analysis | SPSS 15.0 | 3 for both population | “Low Activity”, “Active Leisure”, and “Active Job” |
|  |  |  |  |  |  |  |  |
| Collaborative evaluation and management of students’ health-related physical ﬁtness: applications of cluster analysis and the classiﬁcation tree | Chen et al. (2012) | • Health Grouping/Profiling  • Delivery of healthcare interventions | Dependent variables: Number of metabolic risk factors (e.g. high total cholesterol)  Independent variables: Physical fitness (e.g. 1 min sit-ups, standing long jump) | Classification and Regression Trees (CART) methods | S-Plus 6.2 | 6 | Nil |
|  |  |  | Physical fitness (e.g. 1 min sit-ups, standing long jump) | Hierarchical analysis (Ward's methods) followed by K-means cluster analysis | SPSS 18.0 | 3 | “Good”, “Moderate”, and “Poor” |
|  |  |  |  |  |  |  |  |
| GIS-measured walkability, transit, and recreation environments in relation to older Adults' physical activity: A latent proﬁle analysis | Todd et al. (2016) | • Health Grouping/Profiling | Built environment variables (e.g. residential density and recreation facility density) | Latent profile analysis | Mplus 7.22 | 3 | “Low walkability/  transit/recreation”, “Mean walkability/transit/  recreation”, and “High walkability/transit/  recreation” |
|  |  |  |  |  |  |  |  |
| Hierarchical cluster analysis of labour market regulations and population health: a taxonomy of low- and middle-income countries | Muntaner et al. (2012) | • Resource allocation  • Health Grouping/Profiling | Labor market variables (e.g. average income level and labor force participation gap between female and male workers) | Hierarchical analysis (Ward's method) | Stata 10.0 | 6 | “Residual”, “Emerging”, “Informal”, “Post- Communist”, “Less Successful Informal”, and “Insecure” |
|  |  |  |  |  |  |  |  |
| Identifying Unique Neighborhood Characteristics to Guide Health Planning for Stroke and Heart Attack: Fuzzy Cluster and Discriminant Analyses Approaches | Pedigo et al. (2011) | • Health Grouping/Profiling  • Delivery of healthcare interventions | Socioeconomic and demographic characteristics (e.g. race, gender, age, per capita income, and housing ownership) | Fuzzy K-means cluster analysis | NCSS 2007 | 4 | Nil |
|  |  |  |  |  |  |  |  |
| K-Means Cluster Analysis of Rehabilitation Service Users in the Home Health Care System of Ontario: Examining the Heterogeneity of a Complex Geriatric Population | Armstrong et al. (2012) | • Health Grouping/Profiling  • Delivery of healthcare interventions | Demographics (age, sex), activities of daily living (ADLs), instrumental activities of daily living (IADLs), disease diagnoses (e.g. pressure ulcers), Changes in Health, End-Stage Disease, Symptoms and Signs (CHESS), which is a health instability outcome measure | K-means cluster analysis | SAS 9.1 | 7 | “Dependent and immobile clients with cognitive problems”, “Dependent but mobile clients with cognitive problems”, “Primarily women clients requiring assistance with IADLs and some ADLs” etc. |
|  |  |  |  |  |  |  |  |
| Neighborhood socioeconomic status and food environment: A 20-year longitudinal latent class analysis among CARDIA participants | Richardson et al. (2014) | • Health Grouping/Profiling | Socioeconomic indicators (e.g. income, education, and occupation) | Latent class analysis | Mplus 7 | 4 | “Downwardly mobile neighborhood SES residents”, “Stable low neighborhood SES residents”, “Upwardly mobile neighborhood SES residents”, and “Stable high neighborhood SES residents” |
|  |  |  |  |  |  |  |  |
| Neighborhood typology based on virtual audit of environmental obesogenic characteristics | Feuillet et al. (2015) | • Health Grouping/Profiling | Food environment, recreational facilities and active mobility features | Hierarchical analysis (Ward's method) | R 3.1.1 | 4 | “Green neighborhoods with low residential density”, “Neighborhoods supportive of active mobility”, “High residential density neighborhoods with food and recreational facilities”, and “High residential density neighborhoods with low level of aesthetics” |
|  |  |  |  |  |  |  |  |
| Reducing consumption of confectionery foods: A post-hoc segmentation analysis using a social cognition approach | Naughton et al. (2017) | • Health Grouping/Profiling  • Delivery of healthcare interventions | Social cognition variables (e.g. perceived need to regulate the amount of confectionery foods they consumed) | Latent class analysis | SAS 9.3 | 4 | “Unmotivated”, “Triers”, “Successful actors”, and “Thrivers” |
|  |  |  |  |  |  |  |  |
| Understanding the social patterning of smoking practices: a dynamic typology | Narcisse et al. (2009) | • Health Grouping/Profiling | Smoking practices (cigarette smoking, smoking cessation, and smoking abstinence) | Hierarchical analysis (Ward's method) | N.A. | 6 | “ The abstinents”, “ The ex-smokers”, “ The low addicted”, “ The moderate addicted”, “ The highly addicted”, and “ The chronically addicted” |
|  |  |  |  |  |  |  |  |
| “Do you see what I see?” – Correlates of multidimensional measures of neighborhood types and perceived physical activity–related neighborhood barriers and facilitators for urban youth | Yan et al. (2010) | • Health Grouping/Profiling | Physical urban environment attributes (e.g. road density:) | K-means cluster analysis | SAS 9.2 | 4 | “Arterial development”, “Inner-city area”, “Suburban residential”, and “Central business district” |
|  |  |  |  |  |  |  |  |
| A latent class analysis of cancer risk behaviors among U.S. college students | Kang et al. (2014) | • Health Grouping/Profiling | Cancer risk behaviours and conditions (e.g. tobacco use) | Latent class analysis | R | 4 | Nil |
|  |  |  |  |  |  |  |  |
| A park typology in the QUALITY cohort: Implications for physical activity and truncal fat among youth at risk of obesity | Bird et al. (2016) | • Health Grouping/Profiling | Park features (e.g. cycle path) | Hierarchical analysis (Ward's method) | SAS 9.4 | 9 | “No team sports features; no play area”, “No team sports features; no walking paths”, “Variety of physical activity installations; skate parks; high on incivilities” etc. |
|  |  |  |  |  |  |  |  |
| Adolescent Physical Activity and Sedentary Behavior - Patterning and Long-Term Maintenance | Nelson et al. (2005) | • Health Grouping/Profiling | Physical activity and sedentary behavior (e.g. video/computer gaming) | K-means cluster analysis | SAS | 7 | “ high television/video, video gaming”, “High skating, video gaming”, “High sports participation with parents, high overall sports participation” etc. |
|  |  |  |  |  |  |  |  |
| Anger types and the use of cigarettes and smokeless tobacco among Native American adolescents | Kerby et al. (2003) | • Health Grouping/Profiling | Behavior when angry (e.g. drink alcohol) | Hierarchical analysis (Ward's methods) followed by K-means cluster analysis | SPSS 10.2 | 4 | “Low Response”, “Adaptive”, “Internalizing”, and “Externalizing” |
|  |  |  |  |  |  |  |  |
| Clusters of lifestyle behaviors: Results from the Dutch SMILE study | Vries et al. (2008) | • Health Grouping/Profiling | Health behaviors (e.g. fruit consumption) | Latent class analyses | Latent Gold | 3 | “Healthy”, “Unhealthy”, and “Poor nutrition” |
|  |  |  |  |  |  |  |  |
| Identifying Heterogeneity Among Injection Drug Users: A Cluster Analysis Approach | Shaw et al. (2008) | • Health Grouping/Profiling | Syringe sharing, ethnicity, and types of drugs injected | Hierarchical analysis (Ward's methods) | Stata 9 | 7 | Nil |
|  |  |  |  |  |  |  |  |
| Lifestyle risk factors of students: A cluster analytical approach | Dodd et al. (2010) | • Health Grouping/Profiling  • Delivery of healthcare interventions | Health lifestyle (e.g. binge drinking) | TwoStep Cluster analysis | PASW 17 | 3 | “Unhealthy/high risk”, “Moderately healthy/ moderate risk”, and “Healthy/low risk” |
|  |  |  |  |  |  |  |  |
| Longitudinal Patterns of Health Insurance Coverage Among a National Sample of Children in the Child Welfare System | Raghavan et al. (2008) | • Resource allocation  • Health Grouping/Profiling | Health insurance coverage (yes or no) | Latent class analyses | Stata 9 | 2 | “Gainer” and “Maintainer” |
|  |  |  |  |  |  |  |  |
| Modiﬁable lifestyle behavior patterns, sedentary time and physical activity contexts: A cluster analysis among middle school boys and girls in the SALTA study | Marques et al. (2013) | • Health Grouping/Profiling | Lifestyle risk factors (e.g. BMI) | Hierarchical analysis (Ward's methods) followed by K-means cluster analysis | Stata 12.1 | 4 | “Unhealthy/high-risk” “Moderately healthy/moderate-risk”, “Healthy/low-risk proﬁle except HDL-c”, and “Highly Healthy/low-risk” |
|  |  |  |  |  |  |  |  |
| Neighborhood environment proﬁles related to physical activity and weight status: A latent proﬁle analysis | Adams et al. (2013) | • Health Grouping/Profiling | Built environment features (e.g. residential density) | Latent proﬁle analyses | Mplus 6.0 | 4 | “Low Walkable/Transit and Recreationally Sparse”, “Low Walkable/Recreationally Sparse”, “Moderately Walkable/Recreationally Dense”, “High Walkable/Recreationally Dense” |
|  |  |  |  |  |  |  |  |
| Patterns of Obesogenic Neighborhood Features and Adolescent Weight -  A Comparison of Statistical Approaches | Wall et al. (2012) | • Health Grouping/Profiling | Neighborhood environment features (e.g. green space) | Latent class analysis | Mplus 6.1 | 6 | “Suburban isolated, high SES”, “City residential with parks, nearby convenience food, high SES and transit”, “City residential with parks, nearby convenience food, median SES, low safety and transit” etc. |
|  |  |  |  |  |  |  |  |
| Patterns of Physical Activity Among Older Adults in New York City - A Latent Class Approach | Mooney et al. (2015) | • Health Grouping/Profiling | Physical activity (e.g. endurance exercise duration) | Latent class analysis | R 2.15.3 | 5 | “Least active,” “Walkers,” “Domestic/gardening,” “Athletic,” and “Domestic/gardening athletic” |
|  |  |  |  |  |  |  |  |
| Patterns of sun protective behaviors among Hispanic children in a skin cancer prevention intervention | Miller et al. (2015) | • Health Grouping/Profiling  • Delivery of healthcare interventions | Sun protective behaviors (e.g. the use of sunscreen) | Latent class analysis | Mplus 6.0 | 4 | “Multiple protective behaviors”, “Clothing and shade“, “Pants only”, and “Low/inconsistent protective behaviors” |
|  |  |  |  |  |  |  |  |
| Patterns of Visit Attendance in the Nurse –Family Partnership Program | Holland et al. (2014) | • Resource allocation  • Health Grouping/Profiling | Percentage of recommended attendance achieved | Latent class analysis | SAS 9.3 | 3 | “High attenders”, “Low attenders”, and “Increasing attenders” |
|  |  |  |  |  |  |  |  |
| Patterns of Walkability, Transit, and Recreation Environment for Physical Activity | Adams et al. (2015) | • Health Grouping/Profiling | Built environment features (e.g. public park density) | Latent proﬁle analyses | Mplus 7.11 | 4 | “Low walkability/transit/  recreation”, “Mean walkability/transit/  recreation”, “Moderately high walkability/transit/ recreation”, and “High walkability/transit/  recreation” |
|  |  |  |  |  |  |  |  |
| The clustering of health-related behaviors in a British population sample: Testing for cohort differences | Mawditt et al. (2016) | • Health Grouping/Profiling | Health-related behaviors (e.g. vegetable consumption) | Latent proﬁle analyses | Mplus 7 | 3 | “Risky”, “Moderate Smokers”, and “Mainstream” |
|  |  |  |  |  |  |  |  |
| A Latent Class Analysis of Dissociation and PTSD: Evidence for a Dissociative Subtype | Wolf et al. (2012) | • Health Grouping/Profiling  • Delivery of healthcare interventions | PTSD symptom score (e.g. severity of depersonalization) | Latent proﬁle analyses | Mplus 5.2 | 3 | “Low PTSD”, “High PTSD”, and “High PTSD/high dissociation” |
|  |  |  |  |  |  |  |  |
| A Latent Profile Analysis of Neighborhood Recreation Environments in Relation to Adolescent Physical Activity, Sedentary Time, and Obesity | Norman et al. (2010) | • Health Grouping/Profiling | Neighborhood environmental variables (e.g. size of neighborhood parks) | Latent profile analysis | Mplus 4.2 | 3 | “Open Space”, “Residential with Cul-de-Sacs”, and “Housing & Facility Dense” |
|  |  |  |  |  |  |  |  |
| A typology of neighborhoods and blood pressure in the RECORD Cohort Study | Hulst et al. (2012) | • Health Grouping/Profiling | Neighborhood environmental variables (e.g. concentrations of nitrogen dioxide and particulate matters) | Hierarchical analysis (Ward's methods) | SAS 9.2 | 6 | “Suburban, low social standing”, “Suburban, high social standing”, “Urban, low social standing”, etc. |
|  |  |  |  |  |  |  |  |
| An Investigation of Activity Profiles of Older Adults | Morrow-Howell et al. (2014) | • Health Grouping/Profiling | Physical activity (e.g. sport/exercise intensity) | Latent class analysis | Mplus 5 | 5 | “Low Activity”, “Moderate Activity”, “High Activity”, “Working”, and “Physically Active” |
|  |  |  |  |  |  |  |  |
| Applying Recursive Partitioning to a Prospective Study of Factors Associated with Adherence to Mammography Screening Guidelines | Calvocoressi et al. (2005) | • Health Grouping/Profiling  • Delivery of healthcare interventions | Dependent variable: adherence to mammography screening guidelines  Independent variables:  Risk factors associated with non-adherence (e.g. insurance coverage) | Classification and Regression Trees (CART) methods | Automated CART software | 8 | Nil |
|  |  |  |  |  |  |  |  |
| Associations between food patterns defined by cluster analysis and colorectal cancer incidence in the NIH–AARP diet and health study | Wirfalt et al. (2009) | • Health Grouping/Profiling | Dietary intake (e.g. high-fiber food intake) | K-means cluster analysis | SAS 8.1 | 4 for male  3 for female | “Many foods”, “Vegetables and fruits”, “Fatty meats”, “Fat-reduced foods” etc. |
|  |  |  |  |  |  |  |  |
| Associations of empirically derived eating patterns with plasma lipid biomarkers: a comparison of factor and cluster analysis methods | Newby et al. (2004) | • Health Grouping/Profiling | Dietary intake (e.g. whole grains intake) | K-means cluster analysis | SAS 8.2 | 5 | “Healthy”, “White bread“, “Alcohol”, “Sweets”, and “Meat and potatoes” |
|  |  |  |  |  |  |  |  |
| Built and Social Environments - Associations with Adolescent Overweight and Activity | Nelson et al. (2006) | • Health Grouping/Profiling | Neighborhood environmental variables (e.g. street connectivity) | K-means cluster analysis | SAS 9 | 6 | “Rural working class”, “Exurban”, “Newer suburban”, “Upper-middle class, older suburban” etc. |
|  |  |  |  |  |  |  |  |
| Capturing changes in dietary patterns among older adults: a latent class analysis of an ageing Irish cohort | Harrington et al. (2014) | • Health Grouping/Profiling | Dietary intake (e.g. low-fat dairy intake) | Latent class analysis | Mplus 6.11 | 3 | “Western”, “Healthy”, and “Low-Energy” |
|  |  |  |  |  |  |  |  |
| Characterizing Longitudinal Patterns of Physical Activity in Mid-Adulthood Using Latent Class Analysis: Results From a Prospective Cohort Study | Silverwood et al. (2011) | • Health Grouping/Profiling | Physical activity (e.g. heavy gardening) | Latent class analysis | Mplus 6 | 2 for walking,  2 for cycling,  3 for leisure | “Low activity’’, ‘‘sports and leisure activity’’, ‘‘Gardening and do-it-yourself activities’’ etc. |
|  |  |  |  |  |  |  |  |
| Cluster Analysis and Clinical Asthma Phenotypes | Haldar et al. (2008) | • Health Grouping/Profiling  • Delivery of healthcare interventions | Clinical assessment (e.g. topic status, asthma symptoms), laboratory test (e.g. lung function tests), psychological concerns (e.g. anxiety) | Hierarchical analysis (Ward's methods) followed by K-means cluster analysis | SPSS 14 | 3, 4, 3, for three study population respectively | “Early-onset atopic”, “ and Obese and noneosinophilic”, “Early-onset symptom predominant” etc. |
|  |  |  |  |  |  |  |  |
| Cluster Analysis of Elderly Cardiac Patients’ Prehospital Symptomatology | Lindgren et al. (2008) | • Health Grouping/Profiling  • Delivery of healthcare interventions | Cardiac symptoms (e.g. pain score) | Hierarchical analysis (Ward's methods) | Stata 9 | 3 | “Classic Acute Coronary Syndrome”, “Weary”, and “Diffuse Symptoms” |
|  |  |  |  |  |  |  |  |
| Cluster analysis of symptoms and health seeking behavior differentiates subgroups of patients with severe irritable bowel syndrome | Guthrie et al. (2003) | • Health Grouping/Profiling  • Delivery of healthcare interventions | Bowel symptoms (e.g. abdominal pain), rectal sensitivity and psychological symptoms | K-means cluster analysis | SPSS 10.1 | 3 | Nil |
|  |  |  |  |  |  |  |  |
| Cluster analysis: a useful technique to identify elderly cardiac patients at risk for poor quality of life | Fukuoka et al. (2003) | • Health Grouping/Profiling  • Delivery of healthcare interventions | Cardiac symptoms (e.g. pain score) | Hierarchical analysis (Ward's methods) | Stata 9 | 3 | “Weary,” “Diffuse symptom”, and “Breathless” |
|  |  |  |  |  |  |  |  |
| Clustering of cardiovascular risk factors in Australian adolescents: association with dietary excesses and deficiencies | Milligan et al. (1995) | • Health Grouping/Profiling | Cardiovascular risk factors (e.g. BMI) | K-means cluster analysis | SPSS | 2 | “Higher risk” and “Lower risk” |
|  |  |  |  |  |  |  |  |
| Clustering of health behaviors in adult survivors of childhood cancer and the general population | Rebholz et al. (2012) | • Health Grouping/Profiling | Health behaviors (e.g. smoking) | Latent class analysis | Mplus 6 | 4 | “Risk-avoidance”, “Moderate drinking”, “Risk-taking”, and “Smoking” |
|  |  |  |  |  |  |  |  |
| Clustering of health risk behaviors and the relationship with mental disorders | Vermeulen-Smit et al. (2015) | • Health Grouping/Profiling | Health behaviors (e.g. heavy drinking) | Latent class analysis | Mplus 6.11 | 4 | “Most healthy”, “Smokers, moderate drinkers, inactive, unhealthy diet” etc. |
|  |  |  |  |  |  |  |  |
| Clustering of lifestyle risk behaviors among residents of forty deprived neighborhoods in London: lessons for targeting public health interventions | Watts et al. (2015) | • Health Grouping/Profiling  • Delivery of healthcare interventions | Health behaviors (e.g. smoking) | Latent class analysis | Mplus 7 | 4 | “Minimal behaviours”, “Smoking”, “Maximal behaviours”, and “Sedentary lifestyle” |
|  |  |  |  |  |  |  |  |
| Clustering of modifiable biobehavioral risk factors for chronic disease in US adults: a latent class analysis | Leventhal et al. (2014) | • Health Grouping/Profiling | Health risk factors (e.g. alcohol abuse, obesity) | Latent class analysis | Mplus 6 | 5 | “obese, active non-substance abusers”, “Nicotine-dependent, active, and non-obese” etc. |
|  |  |  |  |  |  |  |  |
| Clustering of Unhealthy Behaviors in the Aerobics Center Longitudinal Study | Héroux et al. (2012) | • Health Grouping/Profiling | Health behaviors (e.g. smoking) | Latent class analysis | SAS 9.1 | 2 | Nil |
|  |  |  |  |  |  |  |  |
| Clustering Women’s Health Behaviors | Hagoel et al. (2002) | • Health Grouping/Profiling | Health behaviors (e.g. smoking) | Hierarchical analysis (Ward's methods) followed by K-means cluster analysis | N.A. | 3 | “Health promoting”, “Inactive”, and “Ambivalent” |
|  |  |  |  |  |  |  |  |
| Comparative Strategies for Using Cluster Analysis to Assess Dietary Patterns | Bailey et al. (2006) | • Health Grouping/Profiling | Dietary intake (e.g. processed meats intake) | K-means cluster analysis | SAS 8 | 2 | Nil |
|  |  |  |  |  |  |  |  |
| Comparing 3 Dietary Pattern Methods—Cluster Analysis, Factor Analysis, and Index Analysis—With Colorectal Cancer Risk | Reedy et al. (2009) | • Health Grouping/Profiling | Dietary intake (e.g. processed meats intake) | K-means cluster analysis | SAS 8.1 | 4 for male,  5 for female | “Many foods”, “Vegetables and fruits”, “Fatty meats”, “Fat-reduced foods”, and “Diet foods/lean meats” |
|  |  |  |  |  |  |  |  |
| Comparison of cluster and principal component analysis techniques to derive dietary patterns in Irish adults | Hearty et al. (2009) | • Health Grouping/Profiling | Dietary intake (e.g. sugar-free drinks) | K-means cluster analysis | SPSS 12 | 6 | “Traditional Irish”, “Continental”, “Unhealthy foods”, “Light-meal foods & low-fat milk”, “Healthy foods”, and “Wholemeal bread & dessert” |
|  |  |  |  |  |  |  |  |
| Developing an empirical typology for regular exercise | Norman et al. (2003) | • Health Grouping/Profiling | Perceptions of exercises (e.g. the benefits of exercise) | Hierarchical analysis (Ward's methods) | N.A. | 6 | “Disengaged”, “Immotive”, “Relapse Risk”, “Early Action”, “Maintainers”, and “Habituated” |
|  |  |  |  |  |  |  |  |
| Dietary patterns among a national random sample of British adults | Pryer et al. (2001) | • Health Grouping/Profiling | Dietary intake (e.g. sugar/confectionery) | Hierarchical analysis (Ward's methods) | SPSS | 4 for both men and women | “Beer and convenience food”, “Traditional British diet”, “Healthier but sweet diet” etc. |
|  |  |  |  |  |  |  |  |
| Dietary patterns among older Europeans: the EPIC-Elderly study | Bamia et al. (2005) | • Health Grouping/Profiling | Dietary intake (e.g. vegetables) | Hierarchical analysis (Ward's methods) | Stata 6.0 | 3 | Nil |
|  |  |  |  |  |  |  |  |
| Dietary patterns and changes in body mass index and waist circumference in adults | Newby et al. (2003) | • Health Grouping/Profiling | Dietary intake (e.g. vegetables) | K-means cluster analysis | SAS 8.2 | 5 | “Healthy”, “White bread”, “Alcohol”, “Sweets”, and “Meat and potatoes” |
|  |  |  |  |  |  |  |  |
| Distinguishing phenotypes of childhood wheeze and cough using latent class analysis | Spycher et al. (2008) | • Health Grouping/Profiling  • Delivery of healthcare interventions | Wheeze and cough symptoms and laboratory tests (e.g. skin-prick tests) | Latent class analysis | Multimix | 3 for wheeze phenotypes, 2 for cough phenotypes | “Persistent cough”, “Transient cough”, “Atopic persistent wheeze”, “Nonatopic persistent wheeze”, and “Transient viral wheeze” |
|  |  |  |  |  |  |  |  |
| Drug use patterns and adherence to treatment among HIV-positive patients: evidence from a large sample of French outpatients (ANRS-EN12-VESPA 2003) | Peretti-Watel et al. (2006) | • Health Grouping/Profiling | Drug use (e.g. cannabis use) | Hierarchical analysis (Ward's methods) | N.A. | 5 | “Low rates for drinking and smoking”, “Daily smokers”, “Alcohol abuse” etc. |
|  |  |  |  |  |  |  |  |
| Food patterns and cardiovascular disease risk factors: The Swedish INTERGENE research program | Berg et al. (2008) | • Health Grouping/Profiling | Dietary intake (e.g. sugar intake) | K-means cluster analysis | SAS 9.1 | 5 | “Healthy”, “Sweet”, “Coffee”, “Traditional” and “Fast energy” |
|  |  |  |  |  |  |  |  |
| Food patterns deﬁned by cluster analysis and their utility as dietary exposure variables: a report from the Malmo Diet and Cancer Study | Wirfalt et al. (1999) | • Health Grouping/Profiling | Dietary intake (e.g. high-fat meat intake) | K-means cluster analysis | SPSS | 6 | “Many foods and drinks”, “Fibre bread”, “ Low fat and high ﬁbre”, White bread”, “Milk fat”, and “Sweets and cakes” |
|  |  |  |  |  |  |  |  |
| Health Lifestyles: Audience Segmentation Analysis for Public Health Interventions | Slater et al. (1991) | • Health Grouping/Profiling  • Delivery of healthcare interventions | Health attitudes, social influences, and behaviors (e.g. smoking) | K-means cluster analysis | SAS | 7 | “Healthful adults”, “Unhealthful adults”, “Worried older adults” etc. |
|  |  |  |  |  |  |  |  |
| Health State Profiles and Service Utilization in Community-Living Elderly | Lafortune et al. (2009) | • Resource allocation  • Health Grouping/Profiling | Chronic diseases (e.g. hypertension),sensory limitations (e.g. vision), functional limitations (e.g. using toilet), cognition, depression, and social demographic variables (e.g. education) | Latent class analysis | Mplus 5 | 4 | “Cognitively and physically impaired”, “Cognitively Impaired”, “Physically impaired”, and “Relatively healthy” |
|  |  |  |  |  |  |  |  |
| Heterogeneity in Hip Fracture Patients: Age, Functional Status, and Comorbidity | Penrod et al. (2007) | • Health Grouping/Profiling  • Delivery of healthcare interventions | Pre-fracture characteristics (e.g. age, comorbidities and independence level of activities of daily livings) | Hierarchical analysis | SPSS | 7 | Nil |
|  |  |  |  |  |  |  |  |
| Identification of asthma clusters in two independent Korean adult asthma cohorts | Kim et al. (2013) | • Health Grouping/Profiling  • Delivery of healthcare interventions | Asthma history (e.g. age of onset), laboratory tests (e.g. FEV1) | Hierarchical analysis (Ward's methods) followed by K-means cluster analysis | SPSS 12.0 | 4 | “Smoking asthma”, “Severe obstructive asthma”, “Early-onset atopic asthma”, and “Late-onset mild asthma” |
|  |  |  |  |  |  |  |  |
| Identiﬁcation of Asthma Phenotypes Using Cluster Analysis in the Severe Asthma Research Program | Moore et al. (2010) | • Health Grouping/Profiling  • Delivery of healthcare interventions | Demographic data, asthma history (e.g. age of onset), laboratory tests (e.g. lung function, atopy). | Hierarchical analysis (Ward's methods) | SAS 9.1 | 5 | Nil |
|  |  |  |  |  |  |  |  |
| Identifying built environmental patterns using cluster analysis and GIS: Relationships with walking, cycling and body mass index in French adults | Charreire et al. (2012) | • Health Grouping/Profiling | Built environment variables (e.g. cycle paths) | Hierarchical analysis (Ward's methods) | SPAD 7.3 | 7 | Nil |
|  |  |  |  |  |  |  |  |
| Identifying mobility heterogeneity in very frail older adults. Are frail people all the same? | Montero-Odasso et al. (2009) | • Health Grouping/Profiling | Self-reported mobility indicators (e.g. difﬁculty raising your arms over your head) | K-means cluster analysis | SAS 9.1 | 3 | “Mild”, “Moderate”, and “Severe” |
|  |  |  |  |  |  |  |  |
| Identifying Patterns of Eating and Physical activity in children: A Latent class analysis of Obesity Risk | Huh et al. (2010) | • Health Grouping/Profiling | Obesity risk factors (e.g. physical activity, eating and sedentary behavior) | Latent class analysis | Mplus 5.0 | 5 | “High-sedentary, high-fat/high-sugar snacks, not weight conscious”, “Dieting without exercise, weight conscious” etc. |
|  |  |  |  |  |  |  |  |
| Identifying risk profiles for childhood obesity using recursive partitioning based on individual, familial, and neighborhood environment factors | Hulst et al. (2015) | • Health Grouping/Profiling | Dependent variables: BMI at baseline and 2-year changes in BMI  Independent variables: Individual (e.g. physical activity), familial (e.g. parental obesity), and neighborhood (e.g. presence pf fast food restaurants) factors | Classification and Regression Trees (CART) methods | R | 7 | Nil |
|  |  |  |  |  |  |  |  |
| Is depression associated with health risk-related behavior clusters in adults? | Verger et al. (2009) | • Health Grouping/Profiling | Health-related behaviors (e.g. tobacco use and alcohol use) | Hierarchical analysis (Ward's methods) | SAS 9 | 5 | “Healthy lifestyles”, “Non-daily- consumers-fruit-and-green-vegetables”, “Regular alcohol users” etc. |
|  |  |  |  |  |  |  |  |
| Latent class analysis applied to health behaviors | Ingledew et al. (1995) | • Health Grouping/Profiling | Health-related behaviors (e.g. healthy eating) | Latent class analysis | MLLSA | 2 | “Healthy behavior” and “More mixed behavior” |
|  |  |  |  |  |  |  |  |
| Latent Class Analysis of Lifestyle Characteristics and Health Risk Behaviors among College Youth | Laska et al. (2009) | • Health Grouping/Profiling | Health-related behaviors (e.g. substance use) | Latent class analysis | SAS 9.1 | 4 | “Poor lifestyle yet low-risk behaviors”, “High risk”, “Moderate lifestyle, few risk behaviors” etc. |
|  |  |  |  |  |  |  |  |
| Latent Transition Analysis: Benefits of a Latent Variable Approach to Modeling Transitions in Substance Use | Lanza et al. (2010) | • Health Grouping/Profiling | Substance use behavior (e.g. marijuana use) | Latent transition analysis | SAS | 4 | “Non-Users”, “Cigarette Smokers”, “Binge Drinkers”, and “Bingers with Marijuana Use” |
|  |  |  |  |  |  |  |  |
| Neighborhood Environment Profiles for Physical Activity Among Older Adults | Adams et al. (2012) | • Health Grouping/Profiling | Built environment features (e.g. residential density) | Latent proﬁle analyses | Mplus 6.0 | 4 for one sub-sample,  3 for the other | “Low Walkable, Transit, and Recreation”, “ Moderately Walkable / Moderately Recreational”, “High Walkable / Recreationally Dense” etc. |
|  |  |  |  |  |  |  |  |
| Obesogenic clusters: multidimensional adolescent obesity- related behaviors in the U.S. | Boone-Heinonen et al. (2008) | • Health Grouping/Profiling | Obesity-related behaviors (e.g. junk food intake) | K-means cluster analysis | SAS 9 | 7 for male  6 for female | “School Clubs & Sports”, “Sports”, “Moderately Active”, “Sedentary Behaviors”, “Junk Food & Smoke” etc. |
|  |  |  |  |  |  |  |  |
| Patterns of health risk behaviors among job-seekers: a latent class analysis | Schnuerer et al. (2015) | • Health Grouping/Profiling | Health-related behaviors (e.g. smoking) | Latent class analysis | Mplus 6.12 | 3 | “Substance use”, “Non- exercising overweight”, and “Health-conscious” |
|  |  |  |  |  |  |  |  |
| Patterns of neighborhood environment attributes related to physical activity across 11 countries: a latent class analysis | Adams et al. (2013) | • Health Grouping/Profiling | Built environment features (e.g. residential density) | Latent class analysis | Mplus 6.0 | 5 | “Overall Activity Supportive”, “High Walkable and Unsafe with Few Recreation Facilities” etc. |
|  |  |  |  |  |  |  |  |
| Patterns of Physical Activity, Sedentary Behavior, and Diet in U.S. Adolescents | Iannotti and Wang (2013) | • Health Grouping/Profiling | Physical activity, sedentary behavior (e.g. television viewing), and dietary intake | Latent class analysis | Mplus 5.1 | 3 | “Healthful”, “Unhealthful”, and “Typical” |
|  |  |  |  |  |  |  |  |
| Patterns of Substance Use in Early Through Late Adolescence | Zapert, Snow, and Tebes (2002) | • Health Grouping/Profiling | Substance use behavior (e.g. marijuana use) | Hierarchical analysis (Ward's methods) | Sleipner | 6 | “Nonusers”, “Alcohol experimenters”, “Low escalators”, “Early starters”, “Late starters”, and “High escalators” |
|  |  |  |  |  |  |  |  |
| Physical activity and sedentary activity patterns among children and adolescents: a latent class analysis approach | Heitzler et al. (2011) | • Health Grouping/Profiling | Physical activity and sedentary behaviors (e.g. watching TV) | Latent class analysis | SAS 9.1 | 3 | “Active”, “Sedentary”, and “Low Media/Moderate Activity” |
|  |  |  |  |  |  |  |  |
| Physical activity and sedentary behavior typologies of 10-11 year olds | Jago et al. (2010) | • Health Grouping/Profiling | Physical activity and sedentary behaviors (e.g. watching TV) | Partition based cluster analysis | SPSS 16 | 3 | “High active/Low sedentary”, “Low active/Moderate sedentary”, and “High Active/High sedentary” |
|  |  |  |  |  |  |  |  |
| Recursive partitioning–based preoperative risk stratification for atrial fibrillation after coronary artery bypass surgery | Sedrakyan et al. (2006) | • Health/Prognostic Index  • Health Grouping/Profiling  • Delivery of healthcare interventions | Dependent variables: AF  Independent variables: Risk factors for postoperative AF (e.g. triple-vessel coronary artery disease) | Classification and Regression Trees (CART) methods | RTREE | 8 | Nil |
|  |  |  |  |  |  |  |  |
| Risk behavior, parental background, and wealth: A cluster analysis among Swedish boys and girls in the HBSC study | Carlerby et al. (2012) | • Health Grouping/Profiling | Health related behaviors (e.g. smoking) | K-means cluster analysis | SPSS 17.0 | 5 | “Low-risk behavior”, “Wish to lose weight”, “Inadequate tooth brushing”, etc. |
|  |  |  |  |  |  |  |  |
| Socioeconomic differences in dietary patterns among middle-aged men and women | Martikainen et al. (2003) | • Health Grouping/Profiling | Dietary intake (e.g. wholemeal bread intake) | K-means cluster analysis | SAS | 6 | “Very healthy”, “Moderately healthy”, “Very unhealthy”, “Sweet” etc. |
|  |  |  |  |  |  |  |  |
| Symptom clustering in advanced cancer | Walsh and Rybicki (2006) | • Health Grouping/Profiling  • Delivery of healthcare interventions | Cancer-related symptoms (e.g. fatigue) | Hierarchical analysis (average linkage) | SAS 6.12 | 7 | “Fatigue: anorexia–cachexia”, “Neuropsychological”, “Upper gastrointestinal” etc. |
|  |  |  |  |  |  |  |  |
| Symptom Clusters and Relationships to Symptom Interference with Daily Life in Taiwanese Lung Cancer Patients | Wang et al. (2008) | • Health Grouping/Profiling  • Delivery of healthcare interventions | Cancer-related symptoms (e.g. fatigue) | Hierarchical analysis (average linkage) | N.A. | 2 | “General Symptom Cluster” and “Gastrointestinal Symptom Cluster” |
|  |  |  |  |  |  |  |  |
| The effect of symptom clusters on functional status and quality of life in women with breast cancer | Dodd et al. (2010) | • Health Grouping/Profiling | Cancer-related symptoms | Hierarchical analysis (average linkage) | SPSS 15.0 | 4 at initial two time points  3 at the final time point | “All low”, “Mild”, “Moderate”, and “All high” |
|  |  |  |  |  |  |  |  |
| The influence of health behavior clusters on dietary change | Reedy et al. (2005) | • Health Grouping/Profiling  • Delivery of healthcare interventions | Fruit and vegetable intake, physical activity, multivitamin use, and BMI | K-means cluster analysis | SAS 8.2 | 5 | “Healthy Choices”, “Eating Well”, “ Physically Active”, “Average Americans”, and “Most Challenged” |
|  |  |  |  |  |  |  |  |
| The internal validity of a dietary pattern analysis. The Framingham Nutrition Studies | Quatromoni et al. (2001) | • Health Grouping/Profiling | Dietary intake (e.g. vegetable intake) | Hierarchical analysis (Ward's methods) | SAS 6 | 5 | “Heart Healthy”, “Light Eating”, “Wine and Moderate Eating”, “High Fat”, and “Empty Calorie” |
|  |  |  |  |  |  |  |  |
| The Structure of Posttraumatic Stress Disorder | Breslau et al. (2005) | • Health Grouping/Profiling  • Delivery of healthcare interventions | PTSD symptoms (e.g. Intrusive memories) | Latent class analysis | Latent Gold | 3 | Nil |
|  |  |  |  |  |  |  |  |
| Tobacco, Marijuana, and Alcohol Use in University Students: A Cluster Analysis | Primack et al. (2012) | • Health Grouping/Profiling | Substance use behaviors (e.g. marijuana use) | TwoStep Cluster analysis | SPSS 11.5 | 6 | “Global Abstainers”, “Drawn to Hookah, Dislike Cigars”, “Marijuana Users, Will Smoke Cigarettes and Drink” etc. |
|  |  |  |  |  |  |  |  |
| Toward an Empirical Taxonomy of Suicide Ideation: A Cluster Analysis of the Youth Risk Behavior Survey | Flannery et al. (2003) | • Health/Prognostic Index  • Health Grouping/Profiling | Health related behaviors (e.g. sexual activity) | Hierarchical analysis (Ward's methods) | SAS | 6 | “Health Safety”, “Poor Health”, “Sexually Active”, “Silent Suicide”, “Moderate Risk”, “Extreme Risk” |
|  |  |  |  |  |  |  |  |
| Tracing the Mediterranean diet through principal components and cluster analyses in the Greek population | Costacou et al. (2003) | • Health Grouping/Profiling | Dietary intake (e.g. vegetables intake) | Hierarchical analysis | SAS | 3 | Nil |
|  |  |  |  |  |  |  |  |
| Trajectories of posttraumatic stress symptomatology in older persons affected by a large-magnitude disaster | Pietrzak et al. (2013) | • Health Grouping/Profiling | PTSD symptoms (e.g. disturbing memories) | Latent growth mixture modeling | Mplus | 3 | “Resistant”, “Chronic”, and “Delayed-onset” |
|  |  |  |  |  |  |  |  |
| Transition to College: Α Classification and Regression Tree (CART) analysis of natural reduction of binge drinking | Vik et al. (2006) | • Health Grouping/Profiling | Dependent variable: heavy drinking  Independent variables: drinking experiences, careless behavior (e.g., missing class), and psychological variables (e.g. anticipated outcome from drinking alcohol) | Classification and Regression Trees (CART) methods | N.A. | 10 | Nil |
|  |  |  |  |  |  |  |  |
| Transitions in drug use among high-risk women: an application of latent class and latent transition analysis | Lanza and Bray et al. (2010) | • Health Grouping/Profiling | Substance use behaviors (e.g. heroin use) | Latent class analysis | SAS 9 | 4 | “Non-users”, “Smokers”, “Moderate Drinkers/ Smokers”, and “Crack, cocaine or heroin users” |
|  |  |  | Substance use behaviors (e.g. heroin use) at multiple time points | Latent transition analysis | SAS 9 | 4 | Same as above |
|  |  |  |  |  |  |  |  |
| Types of alcoholics, I - evidence for an Empirically Derived Typology Based on Indicators of Vulnerability and Severity | Babor et al. (1992) | • Health Grouping/Profiling | Premorbid risk factors (e.g. familial alcoholism), use of substances (e.g. benzodiazepine use), and alcohol-related consequences (e.g. cognitive impairment) | K-means cluster analysis | BMDP | 2 | Nil |
|  |  |  |  |  |  |  |  |
| Typologies of posttraumatic stress disorder in the U.S. adult population | Pietrzak et al. (2014) | • Health Grouping/Profiling | PTSD symptoms (e.g. nightmares) | Latent class analysis | Mplus 7.11 | 3 | “Anxious-Re-experiencing”, “Dysphoric”, and “High Symptom” |
|  |  |  |  |  |  |  |  |
| Typologies of posttraumatic stress disorder in treatment-seeking older adults | Böttche et al. (2015) | • Health Grouping/Profiling | PTSD symptom domains (e.g. avoidance) | Latent profile analysis | Mplus 5.0 | 3 | “Intermediate disturbance”, “Pervasive disturbance low avoidance”, and “Pervasive disturbance high avoidance” |
|  |  |  |  |  |  |  |  |
| A latent class analysis of illicit drug abuse/dependence: results from the National Epidemiological Survey on Alcohol and Related Conditions | Agrawal et al. (2006) | • Health Grouping/Profiling | Substance use (e.g. cannabis use) | Latent class analysis | Mplus 3.1 | 5 | “No abuse/dependence”, “Cannabis abuse/dependence only”, “Stimulants + hallucinogen abuse/dependence” (class 3: 0.6%) etc. |
|  |  |  |  |  |  |  |  |
| A latent class analysis of underage problem drinking: Evidence from a community sample of 16–20 year olds | Reboussin et al. (2006) | • Health/Prognostic Index  • Health Grouping/Profiling | Drinking behaviors (e.g. binge drinking) and alcohol-related problems (e.g. headache) | Latent class analysis | N.A. | 2 | “Risky drinkers” and “Regular drinkers” |
|  |  |  |  |  |  |  |  |
| A longitudinal investigation of the impact of typology of urinary incontinence on quality of life during midlife: Results from a British prospective study | Mishra et al. (2009) | • Health Grouping/Profiling | Urinary incontinence symptoms (e.g. lose urine when coughing, sneezing, laughing, running or exercising) | Longitudinal latent class analysis | Mplus | 4 | “Low symptom”, “Onset”, “Recovering”, and “Chronic” |
|  |  |  |  |  |  |  |  |
| A Longitudinal Typology of Symptoms of Depression and Anxiety Over the Life Course | Colman et al. (2007) | • Health Grouping/Profiling | Anxious and depressive symptoms | Longitudinal latent class analysis | Latent Gold 4.0 | 6 | “Absence of symptoms”, “Adult-onset moderate symptoms”, “Repeated moderate symptoms” etc. |
|  |  |  |  |  |  |  |  |
| An Empirical Study of the Classification of Eating Disorders | Bulik, Sullivan, and Kendler (2000) | • Health Grouping/Profiling  • Delivery of healthcare interventions | Anorectic and bulimic behaviors (e.g. eating binges) | Latent class analysis | FORTRAN | 6 | “Shape/ Weight Preoccupied”, “Low Weight With Binging”, “Low Weight Without Binging” etc. |
|  |  |  |  |  |  |  |  |
| Application of a Latent Class Analysis to Empirically Define Eating Disorder Phenotypes | Keel et al. (2004) | • Health Grouping/Profiling | Eating disorder symptoms (e.g. self-induced vomiting) | Latent class analysis | N.A. | 4 | “Restricting anorexia nervosa”, “Anorexia nervosa and bulimia nervosa with multiple methods of purging” etc. |
|  |  |  |  |  |  |  |  |
| Bone mineral density and dietary patterns in older adults: the Framingham Osteoporosis Study | Tucker et al. (2002) | • Health Grouping/Profiling | Dietary intake (e.g. red meat intake) | K-means cluster analysis | SAS 7 | 6 | “Meat, dairy, and bread”,  “Meat and sweet baked”,  “Sweet baked products”, “Alcohol” etc. |
|  |  |  |  |  |  |  |  |
| Characterization of different groups of elderly according to social engagement activity patterns | Croezen et al. (2009) | • Health Grouping/Profiling  • Delivery of healthcare interventions | Social engagement activity (e.g. work for church) | Hierarchical analysis followed by K-means cluster analysis | SPSS 15.0 | 5 | “Less social engaged elderly”, “Less social engaged caregivers”, “Social engaged caregivers” etc. |
|  |  |  |  |  |  |  |  |
| Classes of disruptive behavior in a sample of young elementary school children | Lier et al. (2003) | • Health Grouping/Profiling | Disruptive behavior (e.g. cruel to animals) | Latent class analysis | Mplus 2.02 | 3 | “High levels of oppositional deﬁant (ODD) problems and attention-deﬁcit/hyperactivity (ADH) problems and intermediate levels of Conduct problems”, “intermediate ODD problems and ADH problems and low levels of Conduct problems”, and “Low levels on all disruptive behaviors” |
|  |  |  |  |  |  |  |  |
| Classification of suicide attempters by cluster analysis: a study of the temperamental heterogeneity in suicidal patients | Engstrom et al. (1996) | • Health Grouping/Profiling | Personality variables (e.g. irritability) | Hierarchical analysis (Ward's methods) | N.A. | 6 | Nil |
|  |  |  |  |  |  |  |  |
| Classification of Suicide Attempters by Cluster Analysis | Paykel et al. (1978) | • Health Grouping/Profiling | Suicidal behavior, its motivation, mental state, and demographic | Hierarchical analysis (Ward's methods) | CLUSTAN | 3 | “Non-overdosers”, “Overdosers”, and “Recurrent” |
|  |  |  |  |  |  |  |  |
| Cluster Analysis Methods Help to Clarify the Activity–BMI Relationship of Chinese Youth | Monda and Popkin et al. (2005) | • Health Grouping/Profiling | Physical activity (e.g. swimming) | K-means cluster analysis | SAS 8.1 | 7 | “Moderate physical activity (PA)/moderate inactivity (IA)”, “Moderate PA/high IA”, “High PA/mod IA” etc. |
|  |  |  |  |  |  |  |  |
| Clustering of dietary variables and other lifestyle factors (Dutch Nutritional Surveillance System) | Huishof et al. (1992) | • Health Grouping/Profiling | Dietary intake (e.g. fiber intake) | K-means cluster analysis | BMDP | 8 | “High fat/low alcohol”, “Moderate fat/low alcohol” etc. |
|  |  |  |  |  |  |  |  |
| Creating Neighborhood Typologies of GIS-Based Data in the Absence of Neighborhood-Based Sampling: A Factor and Cluster Analytic Strategy | Gershoff, Pedersen, and Aber (2009) | • Health/Prognostic Index  • Health Grouping/Profiling | Neighborhood characteristics (e.g. Number of recreational facilities) | Hierarchical analysis | Sleipner 2.1 | 5 | ‘‘Average’’, ‘‘High Risk’’, ‘High Services’’, ‘‘High Flux’’, and ‘‘Low Risk’’ |
|  |  |  |  |  |  |  |  |
| Developmental Typology of Trajectories to Nighttime Bladder Control: Epidemiologic Application of Longitudinal Latent Class Analysis | Croudace et al. (2002) | • Health Grouping/Profiling  • Delivery of healthcare interventions | Bed wetting patterns (e.g. wetting in the past month) | Longitudinal latent class analysis | Mplus 2.12 | 4 | “Normal”, “Persistent”, “Chronic”, “Onset” |
|  |  |  |  |  |  |  |  |
| Dietary patterns and adenocarcinoma of the esophagus and distal stomach | Chen et al. (2002) | • Health Grouping/Profiling | Dietary intake (e.g. red meat intake) | K-means cluster analysis | SAS 6.12 | 6 | “Healthy”, “High meat”, “High salty snacks”, “High dessert”, “High milk”, and “High white bread” |
|  |  |  |  |  |  |  |  |
| Dietary Patterns and Cardiovascular Risk Factors in Elderly Men: The Zutphen Elderly Study | Huijbregts et al. (1995) | • Health Grouping/Profiling | Dietary intake (e.g. fiber intake) | K-means cluster analysis | SAS | 4 | “Alcohol cluster”, “Meat cluster”, “Healthy cluster”, and “Refined sugars cluster” |
|  |  |  |  |  |  |  |  |
| Dietary patterns and lifestyle factors in the Norwegian EPIC cohort: The Norwegian Women and Cancer (NOWAC) study | Engeset et al. (2005) | • Health Grouping/Profiling | Dietary intake (e.g. red meat intake) | K-means cluster analysis | SAS | 6 | “Traditional fish eaters”, “Healthy eaters”, “Average, less fish, less healthy”, “Western”, “Traditional bread eaters”, and “Alcohol users” |
|  |  |  |  |  |  |  |  |
| Dietary Patterns and Nutrient Intakes of 7-Year-Old Children Taking Part in an Atherosclerosis Prevention Project in Finland | Rasanen et al. (2002) | • Health Grouping/Profiling | Dietary intake (e.g. vegetables intake) | K-means cluster analysis | SAS 6.11 | 4 | “1.5% fat milk and butter”, “Sugar and sweets”, “Cereal, rice, and pasta”, and “Bread, skim milk, and margarine” |
|  |  |  |  |  |  |  |  |
| Dietary Patterns and Survival of Older Adults | Anderson et al. (2010) | • Health Grouping/Profiling | Dietary intake (e.g. processed meat intake) | K-means cluster analysis | SAS 9.1 | 6 | “Healthy foods”, “High-fat dairy products”, “Meat, fried foods, and alcohol” etc. |
|  |  |  |  |  |  |  |  |
| Dietary patterns and the adenoma-carcinoma sequence of colorectal cancer | Rouillier et al. (2005) | • Health Grouping/Profiling | Dietary intake (e.g. fruit intake) | Hierarchical analysis (Ward's methods) | SAS 8 | 5 | “Low-energy”, “High-starch, high- fat, and low-fruit”, “High-processed meat, -energy, -alcohol, and -starchy foods” etc. |
|  |  |  |  |  |  |  |  |
| Dietary patterns are associated with lower incidence of type 2 diabetes in middle-aged women: the Shanghai Women’s Health Study | Villegas et al. (2010) | • Health Grouping/Profiling | Dietary intake (e.g. fruit intake) | K-means cluster analysis | SAS | 3 | Nil |
|  |  |  |  |  |  |  |  |
| Dietary Patterns Associated with Risk for Metabolic Syndrome in Urban Community of Karachi Defined by Cluster Analysis | Hydrie et al. (2010) | • Health Grouping/Profiling | Dietary intake (e.g. fruit intake) | K-means cluster analysis | SPSS | 5 | Nil |
|  |  |  |  |  |  |  |  |
| Dietary patterns in middle-aged Irish men and women deﬁned by cluster analysis | Villegas et al. (2004) | • Health Grouping/Profiling | Dietary intake (e.g. fruit intake) | K-means cluster analysis | MINITAB 13 | 3 | “Traditional Diet”, “Prudent Diet”, and “Alcohol & Convenience Foods” |
|  |  |  |  |  |  |  |  |
| Dietary patterns in the Southampton Women’s Survey | Crozier et al. (2006) | • Health Grouping/Profiling | Dietary intake (e.g. fruit intake) | Hierarchical analysis (Ward's methods) followed by K-means cluster analysis | Stata 8 | 2 | “More healthy” and “Less healthy” |
|  |  |  |  |  |  |  |  |
| Dietary patterns of elderly Boston­area residents defined by cluster analysis | Tucker et al. (1992) | • Health Grouping/Profiling | Dietary intake (e.g. fruit intake) | K-means cluster analysis | SPSS 4.1 | 4 | Nil |
|  |  |  |  |  |  |  |  |
| Dietary Patterns of Hispanic Elders Are Associated with Acculturation and Obesity | Lin, Bermudez, and Tucker (2013) | • Health Grouping/Profiling | Dietary intake (e.g. fruit intake) | K-means cluster analysis | SAS 8 | 5 | “Fruit and breakfast cereal”, “Starchy vegetables”, ”Rice”, “Whole milk”, and “Sweets” |
|  |  |  |  |  |  |  |  |
| Dietary patterns of men and women suggest targets for health promotion- the Framingham Nutrition Studies | Millen et al. (1996) | • Health Grouping/Profiling | Dietary intake (e.g. vegetables intake) | Hierarchical analysis (Ward's methods) | SAS | 5 | Nil |
|  |  |  |  |  |  |  |  |
| Dietary Patterns of Rural Older Adults Are Associated with Weight and Nutritional Status | Ledikwe et al. (2004) | • Health Grouping/Profiling | Dietary intake (e.g. vegetables intake) | K-means cluster analysis | SAS 8 | 2 | “Low-Nutrient-Dense” and “High-Nutrient-Dense” |
|  |  |  |  |  |  |  |  |
| Dietary patterns predict the development of overweight in women- The Framingham nutrition studies. | Quatromoni et al. (2002) | • Health Grouping/Profiling | Dietary intake (e.g. vegetables intake) | Hierarchical analysis (Ward's methods) | SAS | 5 | “Heart healthy”, Light eating”, “Wine and moderate eating”, “High fat”, and “Empty calorie” |
|  |  |  |  |  |  |  |  |
| Disentangling women’s responses on complex dietary intake patterns from an Indian cross-sectional survey: a latent class analysis | Padmadas, Dias, and Willekens (2006) | • Health Grouping/Profiling | Dietary intake (e.g. vegetable  intake) | Latent class analysis | MATLAB 6.5 | 5 | “Very high mixed-diet cluster”, “High mixed- diet cluster”, “Moderate mixed-diet cluster” etc. |
|  |  |  |  |  |  |  |  |
| Distinct clinical phenotypes of airways disease defined by cluster analysis | Weatherall et al. (2009) | • Health Grouping/Profiling  • Delivery of healthcare interventions | History of respiratory diseases (e.g. sputum production) and laboratory tests (e.g. FEV1/FVC ratio) | Hierarchical analysis | R | 5 | “Severe and markedly variable airflow obstruction with features of atopic asthma, chronic bronchitis and emphysema”, “Features of emphysema alone” etc. |
|  |  |  |  |  |  |  |  |
| Empirically derived symptom sub-groups correspond poorly with diagnostic criteria for functional dyspepsia and irritable bowel syndrome. A factor and cluster analysis of a patient sample | Eslick et al. (2003) | • Health Grouping/Profiling  • Delivery of healthcare interventions | Gastrointestinal symptoms (e.g. loose/watery stools) | K-means cluster analysis | N.A. | 7 | “Diarrhea”, “Meal-related pain”, “Abdominal pain”, “Faucal indicators”, “Nausea/ vomiting/ weight loss”, “Undifferentiated” and “Constipation” |
|  |  |  |  |  |  |  |  |
| Gastrointestinal Symptoms and Subjects Cluster Into Distinct Upper and Lower Groupings in the Community: A Four Nations Study | Talley et al. (2000) | • Health Grouping/Profiling  • Delivery of healthcare interventions | Gastrointestinal symptoms (e.g. loose/watery stools) | K-means cluster analysis | N.A. | 4 in Rochester, US  5 in Sydney, Australia  6 in Essen, Germany  3 in Osthammar, Sweden | “Reﬂux”, “Bowel Dysfunction”, “Abdominal Pain”, and “Health” |
|  |  |  |  |  |  |  |  |
| Identification and Prediction of Latent Classes of Weight-loss Strategies Among Women | Lanza, Savage, and Birch (2010) | • Health Grouping/Profiling  • Delivery of healthcare interventions | Weight-loss strategies (e.g. fasting) | Latent class analysis | SAS | 4 | “No Weight Loss Strategy”, “Dietary Guidelines”, “Guidelines + Macronutrients”, and “Guidelines + Macronutrients + Restrictive” |
|  |  |  |  |  |  |  |  |
| Identification of groups who report similar patterns of diet among a representative national sample of British adults aged 65 years of age or more | Pryer, Cook, and Shetty (2000) | • Health Grouping/Profiling | Dietary intake (e.g. vegetable  intake) | Hierarchical analysis (Ward's methods) | SPSS | 3 for both male and female elderly population | “Mixed diet”, “healthy diet”, “Traditional diet high in alcohol” etc. |
|  |  |  |  |  |  |  |  |
| Identifying dietary patterns using a normal mixture model: application to the EPIC study | Fahey et al. (2011) | • Health Grouping/Profiling | Dietary intake (e.g. sugar and confectionery intake) | Latent class analysis | Latent Gold 3.0 and 4.0 | 8 | Nil |
|  |  |  |  |  |  |  |  |
| Identifying target segments of male drinkers for health promotion | Wyllie and Casswell (1993) | • Health Grouping/Profiling  • Delivery of healthcare interventions | Drinking attitudes and behaviors (e.g. drinking frequency) | K-means cluster analysis | SAS | 5 | “Light-Drinking”, “Spirits-Drinking Men with Decreased Consumption” etc. |
|  |  |  |  |  |  |  |  |
| Latent Class Analysis Is Useful to Classify Pregnant Women into Dietary Patterns | Sotres-Alvarez, Herring, and Siega-Riz (2010) | • Health Grouping/Profiling | Dietary intake (e.g. folate intake) | Latent class analysis | SAS 9.1 | 3 | “Prudent”, “Health Conscious Western”, and “Hard Core Western” |
|  |  |  |  |  |  |  |  |
| Latent Class Analysis of Lifetime Depressive Symptoms in the National Comorbidity Survey | Sullivan, Kessler, and Kendler (1998) | • Health Grouping/Profiling | Depression symptoms (e.g. insomnia) | Latent class analysis | N.A. | 6 | “Severe Typical”, “Mild Typical”, “Severe Atypical”, “Mild Atypical”, “Intermediate”, and “Minimal Symptoms” |
|  |  |  |  |  |  |  |  |
| Latent Transition Models to Study Women’s Changing of Dietary Patterns From Pregnancy to 1 Year Postpartum | Sotres-Alvarez, Herring, and Siega-Riz (2013) | • Health Grouping/Profiling | Dietary intake (e.g. vegetables intake) | Latent transition analysis | SAS | 3 | “Prudent”, “Health Conscious Western”, and  “Western” |
|  |  |  |  |  |  |  |  |
| Leisure-time physical activity and sedentary behavior clusters and their associations with overweight in middle-aged French adults | Charreire et al. (2010) | • Health Grouping/Profiling | Leisure physical activity and sedentary behavior (e.g. gardening) | Hierarchical analysis (Ward's methods) | SAS 9.3 | 4 for male,  3 for female | “Walking and gardening-low TV”, “Walking and gardening-high TV”, “Multiple activity-low TV” etc. |
|  |  |  |  |  |  |  |  |
| Patterns of health-related behavior and their cross-cultural validity - A comparative study on two populations of young people | Karvonen et al. (2000) | • Health Grouping/Profiling | Health related behaviors (e.g. smoking) | K-means cluster analysis | SPSS 6.12 | 3 | “Healthy”, “Unhealthy”, and “Mix” |
|  |  |  |  |  |  |  |  |
| Relationships of dietary patterns with body composition in older adults differ by gender and PPAR-γ Pro12Ala genotype | Anderson et al. (2010) | • Health Grouping/Profiling | Dietary intake (e.g. vegetables intake) | K-means cluster analysis | SAS 9.1 | 6 | “Healthy foods”, “Meat, snacks, fats and alcohol”, “Sweets and desserts”, “Reﬁned grains”, “Breakfast cereal”, and “High-fat dairy products” |
|  |  |  |  |  |  |  |  |
| Resilience and patterns of health risk behaviors in California adolescents | Mistry et al. (2009) | • Health Grouping/Profiling | Health related behaviors (e.g. smoking) | K-means cluster analysis | Stata 9.2 | 4 | “Salutary Adherents”, “Active Snackers”, “Sedentary Snackers”, and “Risk Takers” |
|  |  |  |  |  |  |  |  |
| Seven unique food consumption patterns identiﬁed among women in the UK Women’s Cohort Study | Greenwood et al. (2000) | • Health Grouping/Profiling | Dietary intake (e.g. vegetables intake) | K-means cluster analysis | SPSS 8 | 7 | “Monotonous low-quantity omnivores”, “Health conscious”, “Traditional meat, chips and pudding eaters” etc. |
|  |  |  |  |  |  |  |  |
| Subgroups of Patients With Cancer With Different Symptom Experiences and Quality-of-Life Outcomes: A Cluster Analysis | Miaskowski et al. (2006) | • Health Grouping/Profiling | Cancer-related symptoms (e.g. fatigue) | Hierarchical analysis (average linkage) | Stata 8.0 and  SAS 9.1 | 4 | “All low”, “High fatigue and low pain”, “Low fatigue and high pain”, “All high” |
|  |  |  |  |  |  |  |  |
| The Structure of Psychosis - Latent Class Analysis of Probands From the Roscommon Family Study | Kendler et al. (1998) | • Health Grouping/Profiling  • Delivery of healthcare interventions | Psychiatric symptoms (e.g. elevated mood) | Latent class analysis | FORTRAN | 6 | “Classic schizophrenia”, “Major depression”, “Schizophreniform disorder”, “Bipolar-schizomania”, “Schizo- depression, and “Hebephrenia” |
|  |  |  |  |  |  |  |  |
| Using Cluster Analysis to Examine Dietary Patterns- Nutrient Intakes, Gender, and Weight Status Differ Across Food Pattern Clusters | Wirfalt and Jeffery (1997) | • Health Grouping/Profiling | Dietary intake (e.g. vegetables intake) | K-means cluster analysis | SAS | 6 | “Soft drinks”, “Pastry”, “Skim milk”, “Meat”, “Meat-cheese”, and “White bread” |
|  |  |  |  |  |  |  |  |
| A Latent Class Analysis of Stigmatizing Attitudes and Knowledge of HIV Risk among Youth in South Africa | Brinkley-Rubinstein et al. (2014) | • Health Grouping/Profiling | Knowledge regarding the risk of contracting HIV (e.g. ‘‘Do you think you can get HIV by shaking hands?’’) | Latent class analysis | Mplus 6 | 4 | “Perceived widespread risk”, “Intercourse- based risk”, “Perceived minimal risk”, “Intimate activity-based risk” |
|  |  |  |  |  |  |  |  |
| A person-centred segmentation study in elderly care: Towards efﬁcient demand-driven care | Laan et al. (2014) | • Resource allocation  • Health Grouping/Profiling  • Delivery of healthcare interventions | Unfulﬁlled biopsychosocial needs (e.g experience emptiness around him/her) | Latent class analysis | Latent Gold 4.5 | 5 | “Feeling vital”, Difﬁculties with psychosocial coping”, “Physical and mobility complaints”, “Difﬁculties experienced in multiple domains”, “Feeling extremely frail” |
|  |  |  |  |  |  |  |  |
| Health status transitions in community-living elderly with complex care needs: a latent class approach | Lafortune et al. (2009) | • Resource allocation  • Health Grouping/Profiling  • Delivery of healthcare interventions | Chronic diseases (e.g. hypertension),sensory limitations (e.g. vision), functional limitations (e.g. using toilet), cognition, depression, and social demographic variables (e.g. education) | Latent transition analysis | Mplus 5 | 4 | “Cognitively & Physically Impaired”, “Cognitively Impaired”, “Physically Impaired”, “Relatively Healthy” |
|  |  |  |  |  |  |  |  |
| Heterogeneity of severe asthma in childhood: Conﬁrmation by cluster analysis of children in the National Institutes of Health/National Heart, Lung, and Blood Institute Severe Asthma Research Program | Fitzpatrick et al. (2011) | • Health Grouping/Profiling  • Delivery of healthcare interventions | Asthma symptoms (e.g. frequency of symptoms), laboratory tests (e.g. lung function test), treatment (e.g. b-agonist use over the previous 3 months), demographics (e.g. age), health care use | Hierarchical analysis (Ward's methods) | SAS 9.1 | 4 | “Late-onset symptomatic asthma”, “Early-onset atopic asthma with normal lung function”, “Early-onset atopic asthma with mild airflow limitation and co-morbidities”, and “Early-onset atopic asthma with advanced airflow limitation” |
|  |  |  |  |  |  |  |  |
| The Clustering of Health Behaviors in Older Australians and its Association with Physical and Psychological Status, and Sociodemographic Indicators | Griffin et al. (2014) | • Health/Prognostic Index  • Health Grouping/Profiling | Behavioral (e.g. diet and cancer screening behaviors) | TwoStep Cluster analysis | SPSS 19.0 | 6 | “Smokers”, “Non-screeners”, “Higher risk ex- smokers”, “Lower risk ex- smokers”, and “ Sedentary non-smokers” |
|  |  |  |  |  |  |  |  |
| The heterogeneous health latent classes of elderly people and their socio-demographic characteristics in Taiwan | Liu et al. (2014) | • Health Grouping/Profiling  • Delivery of healthcare interventions | Chronic conditions (e.g. diabetes), sensory limitations (e.g. problems with hearing), and functional impairments (e.g. difficulty with dressing) | Latent class analysis | Mplus 6.0 | 4 | “High Comorbidity”, “Functional Impairment”, “Frail”, “Relatively Healthy” |
|  |  |  |  |  |  |  |  |
| Utilization of health care services by elderly people with National Health Insurance in Taiwan: The heterogeneous health proﬁle approach | Liu et al. (2012) | • Resource allocation  • Health Grouping/Profiling  • Delivery of healthcare interventions | Chronic conditions (e.g. diabetes), sensory limitations (e.g. problems with hearing), and functional impairments (e.g. difficulty with dressing) | Latent class analysis | Mplus 5.0 | 4 | “High Comorbidity”, “Functional Impairment”, “Frail”, and “Relative Healthy” |
|  |  |  |  |  |  |  |  |
| A Cluster Analysis of Physical Activity and Sedentary Behavior Patterns in Middle School Girls | Trilk et al. (2012) | • Health Grouping/Profiling | Self-reported physical activity | K-means cluster analysis | SAS | 6 | “Educational sedentary”, “Sports and play”, “Organized sports teams/classes/lessons in past year” etc. |
|  |  |  |  |  |  |  |  |
| A hierarchy of sociodemographic and environmental correlates of walking and obesity | Frank et al. (2008) | • Health Grouping/Profiling | Dependent variables: physical walking and weight status  Independent variables: built environment and sociodemographic characteristics that are jointly associated with | Chi-squared Automatic Interaction Detector (CHAID) | N.A. | 11, 18, 17 for three dependent variables respectively | Nil |
|  |  |  |  |  |  |  |  |
| A Latent Class Analysis of DSM-IV and Fagerström (FTND) Criteria for Nicotine Dependence | Agrawal et al. (2011) | • Health Grouping/Profiling | Seven DSM-IV nicotine dependence criteria (e.g. tolerance and withdrawal) and six FTND criteria (e.g. time to first cigarette and smoking where prohibited) | Latent class analyses | Mplus 5.1 | 4 | “High DSM-high FTND”, “Moderate DSM-moderate FTND”, “Low DSM-low FTND”, and “Lighter smokers–moderate FTND” |
|  |  |  |  |  |  |  |  |
| A Latent Class Analysis of Risk Factors for Acquiring HIV Among Men Who Have Sex with Men: Implications for Implementing Pre-Exposure Prophylaxis Programs | Chan et al. (2015) | • Health/Prognostic Index  • Health Grouping/Profiling | HIV related history (e.g. date of last HIV test, number of oral sex partners in the last 12 months) | Latent class analyses | Mplus 7.1 | 4 | Nil |
|  |  |  |  |  |  |  |  |
| A latent class model to identify city/town chronic disease patterns | Jiang et al. (2015) | • Resource allocation  • Health Grouping/Profiling  • Delivery of healthcare interventions | Demographics and socioeconomic status, health behaviors and chronic diseases prevalence, and healthcare utilizations | Latent class analyses | Latent Gold 4.5 | 3 | “Afﬂuent & healthy towns”, “Economically stable towns”, and “With socioeconomic and health challenges” |
|  |  |  |  |  |  |  |  |
| A Latent Transition Model of the Effects of a Teen Dating Violence Prevention Initiative | Williams et al. (2015) | • Health Grouping/Profiling | Teen dating violence related behaviors (e.g. sexual harassment, and bullying) | Latent class and latent transition analysis | Mplus 6 and SAS | 4 | “Multiproblem”, “Bully-harassment victimization”, “Bully-psychological victimization”, and “Bully victimization” |
|  |  |  |  |  |  |  |  |
| Adolescent physical activity and the built environment: A latent class analysis approach | McDonald et al. (2012) | • Health Grouping/Profiling | Neighborhood characteristics (e.g. distance to gym Retail establishment density) | Latent class analysis | SAS 9.1 | 4 | “Low- density retail/transit, low walkability index (WI), further from recreation, “High-density retail/ transit, high WI, closer to recreation” etc. |
|  |  |  |  |  |  |  |  |
| Comparison of Suicide Attempters and Decedents in the U.S. Army: A Latent Class Analysis | Skopp et al. (2016) | • Health/Prognostic Index  • Health Grouping/Profiling | Suicide related indicators (e.g. failed intimate relationship in last 90 days, legal/administrative problems last 90 days) | Latent class analysis | N.A. | 3 | “External/Antisocial Risk Factors”, “Mental Health Risk Factors”, and “No Pattern” |
|  |  |  |  |  |  |  |  |
| Complex Comorbidity Clusters in OEF/OIF Veterans - The Polytrauma Clinical Triad and Beyond | Pugh et al. (2014) | • Health Grouping/Profiling  • Delivery of healthcare interventions | Comorbidity (e.g. posttraumatic stress disorder, hypertension, diabetes) | Latent class analysis | N.A. | 6 | “Polytrauma clinical triad (PCT, traumatic brain injury (TBI), posttraumatic stress disorder (PTSD) and pain) + Chronic Disease”, “ PCT”, “ Mental Health + Substance Abuse” etc. |
|  |  |  |  |  |  |  |  |
| Coping, Stress, and Social Support Associations With Internalizing and Externalizing Behavior Among Urban Adolescents and Young Adults: Revelations From a Cluster Analysis | Tandon et al. (2013) | • Health/Prognostic Index  • Health Grouping/Profiling | Coping, support, and stress subscales | K-means cluster analysis | Stata 9.1 | 3 | “High Risk/High protective”, “Moderate Risk/Moderate protective”, and “Moderate Risk/Lower protective” |
|  |  |  |  |  |  |  |  |
| Differences in environmental preferences towards cycling for transport among adults: a latent class analysis | Mertens et al. (2016) | • Health Grouping/Profiling | Micro-environmental preferences (e.g. traffic density, evenness of the cycle path) | Latent class analysis | Sawtooth Software (SSI Web 8.3.8.) | 3 | Nil |
|  |  |  |  |  |  |  |  |
| Distinct symptom experiences in subgroups of patients with COPD | Christensen et al. (2016) | • Health Grouping/Profiling | Self-reported symptoms (e.g. shortness of breath) | Latent class analysis | Stata/SE 14.1 | 3 | “High”, “Intermediate”, and “Low” |
|  |  |  |  |  |  |  |  |
| Effects of clustering of multiple lifestyle-related behaviors on blood pressure in adolescents from two observational studies | Moraes et al. (2016) | • Health Grouping/Profiling | Health related behaviors (e.g. weekly consumption of fruits and vegetables, weekly consumption of sugar-sweetened beverages) | Hierarchical analysis (Ward's methods) followed by K-means cluster analysis | Stata 13 | 3 | “Sedentary”, “Healthy Eating” etc. |
|  |  |  |  |  |  |  |  |
| Exploring the application of latent class cluster analysis for investigating pedestrian crash injury severities in Switzerland | Sasidharan et al. (2015) | • Health Grouping/Profiling | Crash characteristics (e.g. roadway characteristics, vehicle characteristics, environmental conditions) | Latent class analysis | N.A. | 7 | “Nighttime midblock crashes”, “Driver violating right of way of pedestrians during daytime”, “Weekend night time crashes under inﬂuence” etc. |
|  |  |  |  |  |  |  |  |
| Health lifestyle behaviors among U.S. adults | Onge and Krueger (2017) | • Health Grouping/Profiling | Self-reported health related behaviors (e.g. smoking status, alcohol use, physical activity) | Latent class analysis | N.A. | 7 | Nil |
|  |  |  |  |  |  |  |  |
| Health-Related Fitness Proﬁles in Adolescents With Complex Congenital Heart Disease | Klausen et al. (2015) | • Health Grouping/Profiling | Health-related ﬁtness (e.g. peak oxygen uptake, heart rate (HR) reserve, body mass index) | Hierarchical analysis (Ward's methods) followed by K-means cluster analysis | SPSS 20 | 3 | “Robust”, “Moderately Robust”, and “Less robust” |
|  |  |  |  |  |  |  |  |
| Latent class analysis of acceptability and willingness to pay for self-HIV testing in a United States urban neighborhood with high rates of HIV infection | Nunn et al. (2017) | • Health Grouping/Profiling  • Delivery of healthcare interventions | HIV-risk behaviors (e.g. condomless sex) | Latent class analysis | N.A. | 4 | “Low risk”, “Concurrent partnerships”, “Incarceration and substance use”, and “Condomless sex/ multiple partners” |
|  |  |  |  |  |  |  |  |
| Latent class modelling of the association between socioeconomic background and breast cancer survival status at 5 years incorporating stage of disease | Downing et al. (2010) | • Health Grouping/Profiling | Patient and tumor characteristics (e.g. patient age and tumor stage) | Latent class analysis | Latent GOLD | 2 | Nil |
|  |  |  |  |  |  |  |  |
| Latent Classes of Young adults Based on Use of Multiple types of tobacco and nicotine Products | Erickson, Lenk, and Forster (2014) | • Health Grouping/Profiling | Substance use (e.g. cigarettes in the past 30 days) | Latent class analysis | SAS 9.2 | 5 | “No/limited use”, “Snuff/snus”, “Cigarillos and hookah”, “Cigarette smokers”, and “Poly-users” |
|  |  |  |  |  |  |  |  |
| Latent Homeless Risk Proﬁles of a National Sample of Homeless Veterans and Their Relation to Program Referral and Admission Patterns | Tsai, Kasprow, and Rosenheck (2013) | • Resource allocation  • Health/Prognostic Index  • Health Grouping/Profiling | Homeless risk factors (e.g. Incarceration history) | Latent class analysis | N.A. | 4 | “Relatively few problems”, “Dual diagnosis”, “Poverty–substance abuse–incarceration”, and “Disabling medical problems” |
|  |  |  |  |  |  |  |  |
| Latent variable mixture models to test for differential item functioning: a population- based analysis | Wu et al. (2017) | • Health Grouping/Profiling | Self-report health status measures (e.g. Walking several blocks) | Latent variable mixture models | Mplus 7.11 | 3 for physical functioning and 2 for mental health sub-scales | Nil |
|  |  |  |  |  |  |  |  |
| Pattern Analysis of Suicide Mortality Surveillance Data in Urban South Africa | Burrows and Laflamme (2008) | • Health Grouping/Profiling | Variables descriptive of the events (method, place/scene, day of week, season), their victims (race, age, sex), and the suicide level of the cities | Hierarchical analysis | SPAD 6.5 | 6 | “Suicides among Asians”, “Poisonings among females”, “Suicides among Whites etc. |
|  |  |  |  |  |  |  |  |
| Patterns of Alternative Tobacco Product Use: Emergence of Hookah and E-cigarettes as Preferred Products Amongst Youth | Gilreath et al. (2016) | • Health Grouping/Profiling | Lifetime and current (past 30 days) use of cigarettes, cigars/cigarillos/little cigars, e- cigarettes, hookah/waterpipe, and smokeless/dip/chewing tobacco | Latent class analysis | Mplus 7.3 | 4 | “Nonusers”, “Polytobacco experimenters”, “E-cigarette/hookah users”, and “Polytobacco users” |
|  |  |  |  |  |  |  |  |
| Patterns of Hospitalization Risk for Women Surviving Into Very Old Age | Dolja-Gore et al. (2017) | • Resource allocation  • Health/Prognostic Index  • Health Grouping/Profiling | Hospital admissions | Latent class analysis | SAS 9.4 | 4 | “Low Risk”, “Moderate Risk”, “Increased Risk”, and “High Risk” |
|  |  |  |  |  |  |  |  |
| Patterns of neighborhood environment attributes in relation to children's physical activity | Kurka et al. (2015) | • Health Grouping/Profiling | Neighborhood environment characteristics (e.g. walkability, transit access etc.) | Latent proﬁle analysis | Mplus 7.11 | 4 for San Diego region and 3 for Seattle region | “Low Walkable, Unsafe, Parks and Recreation Sparse”, “Moderate Walkable, Transit Access, and Recreation” etc. |
|  |  |  |  |  |  |  |  |
| Patterns of Physical Activity, Sedentary Behavior and Diet in US Adolescents | Iannotti and Wang (2013) | • Health Grouping/Profiling | Physical Activity, Sedentary Behavior and Diet | Latent class analysis | Mplus 5.1 | 3 | “Healthful”, “Unhealthful”, and “Typical” |
|  |  |  |  |  |  |  |  |
| Prevalence and Patterns of Polysubstance Use in a Nationally Representative Sample of 10th Graders in the United States | Conway et al. (2013) | • Health Grouping/Profiling | Substance use (e.g. use of marijuana) | Latent class analysis | Mplus 6.1 | 4 | “Non-user”, ”Predominant alcohol user”, “Predominant marijuana user”, and “Predominant polysubstance user” |
|  |  |  |  |  |  |  |  |
| Smoking Patterns and their relationship to Drinking among First-Year College students | Hoeppner et al. (2014) | • Health Grouping/Profiling | Cigarette use | Group-based trajectory modeling | SAS | 5 | “Stable Infrequent Smokers”, “Increasing Infrequent Smokers”, “Decreasing Infrequent Smokers”, “Occasional Smokers”, and “Frequent Smokers” |
|  |  |  |  |  |  |  |  |
| Smoking patterns during pregnancy and postnatal period and depressive symptoms | Munafo, Heron, and Araya (2007) | • Health Grouping/Profiling | Maternal smoking status (e.g. number of cigarettes per day) | Longitudinal latent class analysis | Mplus | 7 | “Persistent smokers”, “Temporary quitters (quit early, relapsed early)” etc. |
|  |  |  |  |  |  |  |  |
| Subgrouping outpatients of an environmental medicine unit using SCL-90-R and cluster analysis | Helm and Eis (2007) | • Health Grouping/Profiling | Psychosocial measures (e.g. SCL-90-R scores) | TwoStep Cluster analysis | SPSS | 3 | Nil |
|  |  |  |  |  |  |  |  |
| The Neighbourhood Built Environment and Trajectories of Depression Symptom Episodes in Adults: A Latent Class Growth Analysis | Gariepy et al. (2015) | • Health Grouping/Profiling | Depression symptoms | Latent class growth modelling | Stata 12.1 | 3 | “Low prevalence of depression symptom episodes”, “Moderate prevalence of depression symptom episodes”, and “High prevalence of depression symptom episodes” |
|  |  |  |  |  |  |  |  |
| Time Use and Food Pattern Influences on Obesity | Kolodinsky and Goldstein (2011) | • Health Grouping/Profiling | Expenditure shares for food (e.g. lamb, eggs) and minutes of time use (e.g. sleep, screen time) | TwoStep Cluster analysis | SPSS 15.0 | 5 | “Average”, “Fullfafh”, “Fastfood”, “etc. for food patterns and “Workers”, “Active”, “Household” etc. for time use patterns |
|  |  |  |  |  |  |  |  |
| Tobacco Use and Suicidality: Latent Patterns of Co-occurrence Among Black Adolescents | Gilreath, Connell, and Leventhal (2012) | • Health Grouping/Profiling | Lifetime and recent tobacco use (e.g. number of days smoked in the past 30 days) and suicidality (e.g. whether or not the respondent had ever felt sad/ hopeless for 2 weeks) | Latent class analysis | Mplus 5.21 | 4 for tobacco use behavior and 3 for suicidality | “Nonsmokers”, “Former smokers”, “Light current smokers”, and “Frequent current smokers” for tobacco use behavior and “not suicidal, “mild suicidality”, and “Suicidal” for suicidality |
|  |  |  |  |  |  |  |  |
| Trajectories and predictors of return to work after traumatic limb injury – a 2-year follow-up study | Hou et al. (2012) | • Health Grouping/Profiling | Return to work status | Group-based trajectory model | SAS 9.1.3 | 3 | “Fast RTW”, “Average RTW”, and “Slow RTW” |
|  |  |  |  |  |  |  |  |
| Trajectories of Cigarette smoking From adolescence to adulthood as Predictors of Unemployment status | Brook et al. (2014) | • Health Grouping/Profiling | Cigarette smoking (frequency and quantity) | Growth mixture modeling | Mplus | 5 | “Heavy/continuous smokers”, “Occasional smokers”, “Late-starting smokers”, “Quitters/decreasers”, and “Nonsmokers” |
|  |  |  |  |  |  |  |  |
| Trajectories of Depressive Symptoms Among Web-Based Health Risk Assessment Participants | Bedrosian, Hawrilenko, and Cole-Lewis (2017) | • Health/Prognostic Index  • Health Grouping/Profiling | Depressive symptoms (Center for Epidemiological Studies Depression Scale) | Growth mixture modeling | Mplus 7.3 | 5 | “Minimal depression”, “Low risk”, “Deteriorating”, “Chronic”, and “Remitting” |
|  |  |  |  |  |  |  |  |
| Trajectories of Depressive Symptoms in Canadian Emerging Adults | Ferro, Gorter, and Boyle (2015) | • Health Grouping/Profiling | Depressive symptoms (Center for Epidemiological Studies Depression Scale) | Latent class growth modeling | SAS 9.4 | 3 | “Minimal”, “Subclinical”, and “Clinical” |
|  |  |  |  |  |  |  |  |
| Trajectories of Kinematic Risky Driving Among Novice Teenagers | Simons-Morton et al. (2013) | • Health/Prognostic Index  • Health Grouping/Profiling | Driving performance and driver characteristics (e.g. lateral negative/left turn ≤ −0.50 g) | Latent class analysis | R | 2 | “Higher-risk and” Lower-risk” |
|  |  |  |  |  |  |  |  |
| Trajectories of Loneliness in Adolescents With Congenital Heart Disease: Associations With Depressive Symptoms and Perceived Health | Vanhalst et al. (2013) | • Health Grouping/Profiling | Loneliness (University of California Los Angeles Loneliness Scale) | Latent class growth analysis | N.A. | 5 | “Chronically high”, “High decreasing”, “Moderate stable”, “Low increasing”, and “Stable low” |
|  |  |  |  |  |  |  |  |
| Trajectories of physical functioning and their prognostic indicators: A prospective cohort study in older adults with joint pain and comorbidity | Hermsen et al. (2014) | • Health Grouping/Profiling | Physical functioning (RAND-36 PF subscale) | Latent class growth modelling | Mplus 7.11 | 3 | “Good PF”, “Moderate PF”, and “Poor PF” |
|  |  |  |  |  |  |  |  |
| Trajectories of productivity loss over a 20-year period: an analysis of the National Longitudinal Survey of Youth | Besen and Pransky (2014) | • Health/Prognostic Index  • Health Grouping/Profiling | Health-related productivity loss (e.g. health fully preventing a person from working) | Latent class growth analysis | Stata 13.1 | 5 | “No risk”, “Low risk”, “High risk”, “Increasing risk at early ages”, and “Increasing risk at later ages” |
|  |  |  |  |  |  |  |  |
| Trajectories of Social Engagement and Limitations in Late Life | Thomas (2011) | • Health Grouping/Profiling | Social engagement levels (e.g. how many hours were spent on volunteer work in the past 12 months) | Growth mixture modeling | Mplus 5 | 5 | Nil |
|  |  |  |  |  |  |  |  |
| Trajectories of Suicidal Ideation from Sixth through Tenth Grades in Predicting Suicide Attempts in Young Adulthood in an Urban African American Cohort | Musci et al. (2016) | • Health/Prognostic Index  • Health Grouping/Profiling | Suicidal ideation | Longitudinal latent class analysis | Mplus 7.1 | 2 | “Ideators” and “Nonideators” |
|  |  |  |  |  |  |  |  |
| Trajectories or Parental Monitoring and Communication and Effects on Drug Use Among Urban Young Adolescents | Tobler and Komro (2010) | • Health/Prognostic Index  • Health Grouping/Profiling | Parental monitoring and communication (e.g. eat dinner with a parent or guardian?) | Multilevel general growth mixture modeling | Mplus | 4 | “High”, “Medium”, “Decreasing”, and “Inconsistent” |
|  |  |  |  |  |  |  |  |
| Trajectory Classes of Decline in Health-Related Quality of Life in Parkinson’s Disease: A Pilot Study | Klotsche et al. (2011) | • Health Grouping/Profiling | HRQoL data (EuroQol, Parkinson’s Disease Questionnaire [PDQ]-39) | Latent growth mixture model | Stata 10.2 | 4 | Nil |
|  |  |  |  |  |  |  |  |
| Transitions in Smokers’ Social Networks After Quit Attempts: A Latent Transition Analysis | Bray et al. (2016) | • Health/Prognostic Index  • Health Grouping/Profiling | Social network variables (e.g. network size, new network members, members’ smoking habits) | Latent class analysis and latent transition analysis | N.A. | 5 | “Immersed”, “Low Smoking Exposure”, “Smoking Partner”, “Isolated”, and “Distant Smoking Exposure” |
|  |  |  |  |  |  |  |  |
| Transitions in Suicide Risk in a Nationally Representative Sample of Adolescents | Thompson, Kuruwita, and Foster (2009) | • Health/Prognostic Index  • Health Grouping/Profiling | Suicide risk (e.g. family and friend history of suicidal behavior) | Latent class analysis and latent transition analysis | Mplus | 3 | “Low risk”, “Medium risk”, “High risk” |
|  |  |  |  |  |  |  |  |
|  |  |  |  |  |  |  |  |

**Abbreviations:**

GP: General Practitioner; US: United States; UK: United Kingdom; GIS: Geographic information systems; PTSD: Post-traumatic stress disorder; BMI: Body mass index; SBP: Systolic blood pressure; DBP: Diastolic blood pressure; IBS: Irritable bowel syndrome; FEV1: Forced expiratory volume during the first second; AF: Atrial fibrillation; CABG: Coronary artery bypass grafting HIV: Human immunodeficiency virus; HRQoL: Health related quality of life BMD: Bone mineral density DSM: Diagnostic and Statistical Manual of Mental Disorders; SES: Socioeconomic status; COPD: Chronic obstructive pulmonary disease;
