# Supplementary material for: A systematic review of the clinical application of data-driven population segmentation analysis
Source: BMC Med Res Methodol. 2018 Nov 3;18:121. doi: 10.1186/s12874-018-0584-9 (PMC6215625; doi:10.1186/s12874-018-0584-9)
Supplement: Supplementary file 5 — Table S3. Evaluation of segmentation outcome in studies included for this systematic review. This files includes the detailed assessment of segmentation outcome in studies included for this systematic review, including their internal validation, external validation and validation variables used, identifiability/interpretability, substantiality, stability, and actionability/accessibility (DOCX 160 kb) [file 12874_2018_584_MOESM5_ESM.docx]

**Table S3. Evaluation of segmentation outcome in studies included for this systematic review**

| **Studies** | **Author** | **Internal validation** | **External validation and**  **validation variables used** | **Identiﬁability/**  **Interpretability** | **Substantiality** | **Stability** | **Actionability/**  **Accessibility** |
| --- | --- | --- | --- | --- | --- | --- | --- |
| A quantitative evidence base for population health: applying utilization- based cluster analysis to segment a patient population | Vuik et al. (2016) | Yes | Disease conditions (e.g. diabetes) | Yes | Yes | Nil | Yes |
|  |  |  |  |  |  |  |  |
| A Typology of Predictive Risk Factors for Non- Adherent Medication-Related Behaviors among Chronic Non-Cancer Pain Patients Prescribed Opioids: A Cohort Study | Peacock et al. (2016) | Yes | Non-adherent behavior | Yes | Yes | Nil | Yes |
|  |  |  |  |  |  |  |  |
| Classification and Regression Tree Uncovered Hierarchy of Psychosocial Determinants Underlying Quality Of Life Response Shift in HIV/AIDS | Li et al. (2009) | Yes | Nil | Yes | Yes | Nil | Yes |
|  |  |  |  |  |  |  |  |
| Clustering of adolescent health concerns: A latent class analysis of school students in New Zealand | Noel et al. (2016) | Yes | Nil | Yes | Yes | Nil | Yes |
|  |  |  |  |  |  |  |  |
| Continuum of Mammography Use among US Women: Classification Tree Analysis | Gjelsvik et al. (2014) | Yes | Nil | Yes | Yes | Nil | Yes |
|  |  |  |  |  |  |  |  |
| Exploring Statistical Approaches to Diminish Subjectivity of Cluster Analysis to Derive Dietary Patterns-The Tomorrow Project | Siou et al. (2011) | Yes | Nil | Yes | Yes | Nil | Yes |
|  |  | Yes | Anthropometric variables (e.g. BMI) and physical activity | Yes | Yes | Nil | Yes |
|  |  | Yes | Nil | Yes | Yes | Nil | Yes |
|  |  |  |  |  |  |  |  |
| Identifying adult asthma phenotypes using a clustering approach | Siroux et al. (2011) | Yes | Quality of life, inflammatory markers (blood eosinophil and neutrophil counts) | Yes | Yes | Nil | Yes |
|  |  |  |  |  |  |  |  |
| Latent typologies of posttraumatic stress disorder in World Trade Center responders | Horn et al. (2011) | Yes | Alcohol use problem, depression, functional impairment | Yes | Yes | Nil | Yes |
|  |  |  |  |  |  |  |  |
| Signiﬁcance of Symptom Clustering in Palliative Care of Advanced Cancer Patients | Tsai et al. (2010) | Yes | Survival, functional status, bone metastasis status, physical examination findings,  psychological distress | Yes | Yes | Nil | Yes |
|  |  |  |  |  |  |  |  |
| Snoring, mouth-breathing, and apnea trajectories in a population-based cohort followed from infancy to 81 months: A cluster analysis | Freeman et al. (2012) | Yes | Risks of tonsillectomies and wheezing frequency | Yes | Yes | Nil | Yes |
|  |  |  |  |  |  |  |  |
| Ten-Year Follow-up of Cluster-based Asthma Phenotypes in Adults | Boudier et al. (2013) | Yes | Asthma exacerbations | Yes | Yes | Yes | Yes |
|  |  |  |  |  |  |  |  |
| The clustering of health behaviors in Ireland and their relationship with mental health, self- rated health and quality of life | Conry et al. (2011) | Yes | Mental health, self-rated health and quality of life. | Yes | Yes | Nil | Yes |
|  |  |  |  |  |  |  |  |
| Who does not reduce their sodium intake despite being advised to do so? A population segmentation analysis | Li et al. (2017) | Yes | Nil | Yes | Yes | Nil | Yes |
|  |  |  |  |  |  |  |  |
| A Classification and Regression Tree for Predicting Recurrent Falling among Community- dwelling Seniors Using Home-care Services | Leclerc et al. (2009) | Yes | Nil | Yes | Yes | Nil | Yes |
|  |  |  |  |  |  |  |  |
| Adults' Physical Activity Patterns across Life Domains: Cluster Analysis with Replication | Rovniak et al. (2010) | Yes | Objectively measured physical activity, psychosocial, and built environment (e.g. pedestrian traffic safety) | Yes | Yes | Nil | Yes |
|  |  |  |  |  |  |  |  |
| Collaborative evaluation and management of students’ health-related physical ﬁtness: applications of cluster analysis and the classiﬁcation tree | Chen et al. (2012) | Yes | Nil | Yes | Yes | Nil | Yes |
|  |  |  | Biochemical measurements (e.g. total cholesterol, blood pressure). | Yes | Yes | Nil | Yes |
|  |  |  |  |  |  |  |  |
| GIS-measured walkability, transit, and recreation environments in relation to older Adults' physical activity: A latent proﬁle analysis | Todd et al. (2016) | Yes | Accelerometer-measured moderate-to-vigorous physical activity, sedentary time and self-reported physical activity | Yes | Yes | Nil | Yes |
|  |  |  |  |  |  |  |  |
| Hierarchical cluster analysis of labour market regulations and population health: a taxonomy of low- and middle-income countries | Muntaner et al. (2012) | Yes | Adult mortality, healthy life expectancy, infant mortality, maternal mortality, neonatal mortality, under-5 mortality, and years of life lost to diseases. | Yes | Yes | Nil | Nil |
|  |  |  |  |  |  |  |  |
| Identifying Unique Neighborhood Characteristics to Guide Health Planning for Stroke and Heart Attack: Fuzzy Cluster and Discriminant Analyses Approaches | Pedigo et al. (2011) | Yes | Stroke and MI mortality risks | Yes | Yes | Nil | Yes |
|  |  |  |  |  |  |  |  |
| K-Means Cluster Analysis of Rehabilitation Service Users in the Home Health Care System of Ontario: Examining the Heterogeneity of a Complex Geriatric Population | Armstrong et al. (2012) | Yes | 1-year service outcomes (e.g. successful completion of care plan and mortality) | Yes | Yes | Nil | Yes |
|  |  |  |  |  |  |  |  |
| Neighborhood socioeconomic status and food environment: A 20-year longitudinal latent class analysis among CARDIA participants | Richardson et al. (2014) | Yes | Neighborhood food environment (e.g. counts of chain fast-food restaurants | Yes | Yes | Nil | Yes |
|  |  |  |  |  |  |  |  |
| Neighborhood typology based on virtual audit of environmental obesogenic characteristics | Feuillet et al. (2015) | Yes | Nil | Yes | Yes | Nil | Yes |
|  |  |  |  |  |  |  |  |
| Reducing consumption of confectionery foods: A post-hoc segmentation analysis using a social cognition approach | Naughton et al. (2017) | Yes | Sugar consumption g/d | Yes | Yes | Nil | Yes |
|  |  |  |  |  |  |  |  |
| Understanding the social patterning of smoking practices: a dynamic typology | Narcisse et al. (2009) | Yes | Nil | Yes | Yes | Nil | Yes |
|  |  |  |  |  |  |  |  |
| “Do you see what I see?” – Correlates of multidimensional measures of neighborhood types and perceived physical activity–related neighborhood barriers and facilitators for urban youth | Yan et al. (2010) | Yes | Nil | Yes | Yes | Nil | Yes |
|  |  |  |  |  |  |  |  |
| A latent class analysis of cancer risk behaviors among U.S. college students | Kang et al. (2014) | Yes | Nil | Yes | Yes | Nil | Yes |
|  |  |  |  |  |  |  |  |
| A park typology in the QUALITY cohort: Implications for physical activity and truncal fat among youth at risk of obesity | Bird et al. (2016) | Yes | Physical activity and truncal fat | Yes | Yes | Nil | Yes |
|  |  |  |  |  |  |  |  |
| Adolescent Physical Activity and Sedentary Behavior - Patterning and Long-Term Maintenance | Nelson et al. (2005) | Yes | The likelihood of meeting national activity recommendations in adulthood | Yes | Yes | Nil | Yes |
|  |  |  |  |  |  |  |  |
| Anger types and the use of cigarettes and smokeless tobacco among Native American adolescents | Kerby et al. (2003) | Yes | Nil | Yes | Yes | Nil | Yes |
|  |  |  |  |  |  |  |  |
| Clusters of lifestyle behaviors: Results from the Dutch SMILE study | Vries et al. (2008) | Yes | Nil | Yes | Yes | Nil | Yes |
|  |  |  |  |  |  |  |  |
| Identifying Heterogeneity Among Injection Drug Users: A Cluster Analysis Approach | Shaw et al. (2008) | Yes | Infection risk | Yes | Yes | Nil | Yes |
|  |  |  |  |  |  |  |  |
| Lifestyle risk factors of students: A cluster analytical approach | Dodd et al. (2010) | Yes | Nil | Yes | Yes | Nil | Yes |
|  |  |  |  |  |  |  |  |
| Longitudinal Patterns of Health Insurance Coverage Among a National Sample of Children in the Child Welfare System | Raghavan et al. (2008) | Yes | Nil | Yes | Yes | Nil | Yes |
|  |  |  |  |  |  |  |  |
| Modiﬁable lifestyle behavior patterns, sedentary time and physical activity contexts: A cluster analysis among middle school boys and girls in the SALTA study | Marques et al. (2013) | Yes | Physical activity | Yes | Yes | Nil | Yes |
|  |  |  |  |  |  |  |  |
| Neighborhood environment proﬁles related to physical activity and weight status: A latent proﬁle analysis | Adams et al. (2013) | Yes | Physical activity and BMI | Yes | Yes | Nil | Yes |
|  |  |  |  |  |  |  |  |
| Patterns of Obesogenic Neighborhood Features and Adolescent Weight -  A Comparison of Statistical Approaches | Wall et al. (2012) | Yes | BMI | Yes | Yes | Nil | Yes |
|  |  |  |  |  |  |  |  |
| Patterns of Physical Activity Among Older Adults in New York City - A Latent Class Approach | Mooney et al. (2015) | Yes | BMI | Yes | Yes | Nil | Yes |
|  |  |  |  |  |  |  |  |
| Patterns of sun protective behaviors among Hispanic children in a skin cancer prevention intervention | Miller et al. (2015) | Yes | Nil | Yes | Yes | Nil | Yes |
|  |  |  |  |  |  |  |  |
| Patterns of Visit Attendance in the Nurse –Family Partnership Program | Holland et al. (2014) | Yes | Child outcome (e.g. academic achievement) | Yes | Yes | Nil | Yes |
|  |  |  |  |  |  |  |  |
| Patterns of Walkability, Transit, and Recreation Environment for Physical Activity | Adams et al. (2015) | Yes | Physical activity and BMI | Yes | Yes | Nil | Yes |
|  |  |  |  |  |  |  |  |
| The clustering of health-related behaviors in a British population sample: Testing for cohort differences | Mawditt et al. (2016) | Yes | Nil | Yes | Yes | Nil | Yes |
|  |  |  |  |  |  |  |  |
| A Latent Class Analysis of Dissociation and PTSD: Evidence for a Dissociative Subtype | Wolf et al. (2012) | Yes | Nil | Yes | Yes | Nil | Yes |
|  |  |  |  |  |  |  |  |
| A Latent Profile Analysis of Neighborhood Recreation Environments in Relation to Adolescent Physical Activity, Sedentary Time, and Obesity | Norman et al. (2010) | Yes | Physical activity, sedentary time, and obesity | Yes | Yes | Nil | Yes |
|  |  |  |  |  |  |  |  |
| A typology of neighborhoods and blood pressure in the RECORD Cohort Study | Hulst et al. (2012) | Yes | SBP and DBP | Yes | Yes | Nil | Yes |
|  |  |  |  |  |  |  |  |
| An Investigation of Activity Profiles of Older Adults | Morrow-Howell et al. (2014) | Yes | Self-reported well-being | Yes | Yes | Nil | Yes |
|  |  |  |  |  |  |  |  |
| Applying Recursive Partitioning to a Prospective Study of Factors Associated with Adherence to Mammography Screening Guidelines | Calvocoressi et al. (2005) | Yes | Nil | Yes | Yes | Nil | Yes |
|  |  |  |  |  |  |  |  |
| Associations between food patterns defined by cluster analysis and colorectal cancer incidence in the NIH–AARP diet and health study | Wirfalt et al. (2009) | Yes | Colorectal cancer incidence | Yes | Yes | Nil | Yes |
|  |  |  |  |  |  |  |  |
| Associations of empirically derived eating patterns with plasma lipid biomarkers: a comparison of factor and cluster analysis methods | Newby et al. (2004) | Yes | Blood lipid profile | Yes | Yes | Nil | Yes |
|  |  |  |  |  |  |  |  |
| Built and Social Environments - Associations with Adolescent Overweight and Activity | Nelson et al. (2006) | Yes | Physically activity and BMI | Yes | Yes | Nil | Yes |
|  |  |  |  |  |  |  |  |
| Capturing changes in dietary patterns among older adults: a latent class analysis of an ageing Irish cohort | Harrington et al. (2014) | Yes | Health outcome (e.g. BMI, HbA1c, and SBP) | Yes | Yes | Nil | Yes |
|  |  |  |  |  |  |  |  |
| Characterizing Longitudinal Patterns of Physical Activity in Mid-Adulthood Using Latent Class Analysis: Results From a Prospective Cohort Study | Silverwood et al. (2011) | Yes | Nil | Yes | Yes | Nil | Yes |
|  |  |  |  |  |  |  |  |
| Cluster Analysis and Clinical Asthma Phenotypes | Haldar et al. (2008) | Yes | Asthma exacerbation frequency, inhaled corticosteroid dose | Yes | Yes | Nil | Yes |
|  |  |  |  |  |  |  |  |
| Cluster Analysis of Elderly Cardiac Patients’ Prehospital Symptomatology | Lindgren et al. (2008) | Yes | Quality of Life and psychological distress | Yes | Yes | Nil | Yes |
|  |  |  |  |  |  |  |  |
| Cluster analysis of symptoms and health seeking behavior differentiates subgroups of patients with severe irritable bowel syndrome | Guthrie et al. (2003) | Yes | Nil | Yes | Yes | Nil | Yes |
|  |  |  |  |  |  |  |  |
| Cluster analysis: a useful technique to identify elderly cardiac patients at risk for poor quality of life | Fukuoka et al. (2003) | Yes | Quality of Life and psychological distress | Yes | Yes | Nil | Yes |
|  |  |  |  |  |  |  |  |
| Clustering of cardiovascular risk factors in Australian adolescents: association with dietary excesses and deficiencies | Milligan et al. (1995) | Yes | Nil | Yes | Yes | Nil | Yes |
|  |  |  |  |  |  |  |  |
| Clustering of health behaviors in adult survivors of childhood cancer and the general population | Rebholz et al. (2012) | Yes | Nil | Yes | Yes | Nil | Yes |
|  |  |  |  |  |  |  |  |
| Clustering of health risk behaviors and the relationship with mental disorders | Vermeulen-Smit et al. (2015) | Yes | Nil | Yes | Yes | Nil | Yes |
|  |  |  |  |  |  |  |  |
| Clustering of lifestyle risk behaviors among residents of forty deprived neighborhoods in London: lessons for targeting public health interventions | Watts et al. (2015) | Yes | Nil | Yes | Yes | Nil | Yes |
|  |  |  |  |  |  |  |  |
| Clustering of modifiable biobehavioral risk factors for chronic disease in US adults: a latent class analysis | Leventhal et al. (2014) | Yes | Nil | Yes | Yes | Nil | Yes |
|  |  |  |  |  |  |  |  |
| Clustering of Unhealthy Behaviors in the Aerobics Center Longitudinal Study | Héroux et al. (2012) | Yes | All-cause mortality | Yes | Yes | Nil | Yes |
|  |  |  |  |  |  |  |  |
| Clustering Women’s Health Behaviors | Hagoel et al. (2002) | Yes | Mammography screening compliance | Yes | Yes | Nil | Yes |
|  |  |  |  |  |  |  |  |
| Comparative Strategies for Using Cluster Analysis to Assess Dietary Patterns | Bailey et al. (2006) | Yes | Nil | Yes | Yes | Nil | Yes |
|  |  |  |  |  |  |  |  |
| Comparing 3 Dietary Pattern Methods—Cluster Analysis, Factor Analysis, and Index Analysis—With Colorectal Cancer Risk | Reedy et al. (2009) | Yes | Colorectal cancer incidence | Yes | Yes | Nil | Yes |
|  |  |  |  |  |  |  |  |
| Comparison of cluster and principal component analysis techniques to derive dietary patterns in Irish adults | Hearty et al. (2009) | Yes | Nil | Yes | Yes | Nil | Yes |
|  |  |  |  |  |  |  |  |
| Developing an empirical typology for regular exercise | Norman et al. (2003) | Yes | Exercise behavior | Yes | Yes | Nil | Yes |
|  |  |  |  |  |  |  |  |
| Dietary patterns among a national random sample of British adults | Pryer et al. (2001) | Yes | Other health behaviors (e.g. smoking) | Yes | Yes | Nil | Yes |
|  |  |  |  |  |  |  |  |
| Dietary patterns among older Europeans: the EPIC-Elderly study | Bamia et al. (2005) | Yes | Nil | Yes | Yes | Nil | Yes |
|  |  |  |  |  |  |  |  |
| Dietary patterns and changes in body mass index and waist circumference in adults | Newby et al. (2003) | Yes | BMI and waist circumference | Yes | Yes | Nil | Yes |
|  |  |  |  |  |  |  |  |
| Distinguishing phenotypes of childhood wheeze and cough using latent class analysis | Spycher et al. (2008) | Yes | Future wheeze, chronic cough and inhaler use | Yes | Yes | Nil | Yes |
|  |  |  |  |  |  |  |  |
| Drug use patterns and adherence to treatment among HIV-positive patients: evidence from a large sample of French outpatients (ANRS-EN12-VESPA 2003) | Peretti-Watel et al. (2006) | Yes | Non-adherence to HAART | Yes | Yes | Nil | Yes |
|  |  |  |  |  |  |  |  |
| Food patterns and cardiovascular disease risk factors: The Swedish INTERGENE research program | Berg et al. (2008) | Yes | Cardiovascular risk factors (e.g. blood pressure and plasma glucose) | Yes | Yes | Nil | Yes |
|  |  |  |  |  |  |  |  |
| Food patterns deﬁned by cluster analysis and their utility as dietary exposure variables: a report from the Malmo Diet and Cancer Study | Wirfalt et al. (1999) | Yes | BMI | Yes | Yes | Nil | Yes |
|  |  |  |  |  |  |  |  |
| Health Lifestyles: Audience Segmentation Analysis for Public Health Interventions | Slater et al. (1991) | Yes | Future health behavior (e.g. use of vitamins) | Yes | Yes | Nil | Yes |
|  |  |  |  |  |  |  |  |
| Health State Profiles and Service Utilization in Community-Living Elderly | Lafortune et al. (2009) | Yes | Healthcare utilization | Yes | Yes | Nil | Yes |
|  |  |  |  |  |  |  |  |
| Heterogeneity in Hip Fracture Patients: Age, Functional Status, and Comorbidity | Penrod et al. (2007) | Yes | 6-month follow up functional outcomes (e.g. independence level of activities of daily livings) | Yes | Yes | Nil | Yes |
|  |  |  |  |  |  |  |  |
| Identification of asthma clusters in two independent Korean adult asthma cohorts | Kim et al. (2013) | Yes | Nil | Yes | Yes | Nil | Yes |
|  |  |  |  |  |  |  |  |
| Identiﬁcation of Asthma Phenotypes Using Cluster Analysis in the Severe Asthma Research Program | Moore et al. (2010) | Yes | Nil | Yes | Yes | Nil | Yes |
|  |  |  |  |  |  |  |  |
| Identifying built environmental patterns using cluster analysis and GIS: Relationships with walking, cycling and body mass index in French adults | Charreire et al. (2012) | Yes | Physical activity (e.g. walk and cycle) and BMI | Yes | Yes | Nil | Yes |
|  |  |  |  |  |  |  |  |
| Identifying mobility heterogeneity in very frail older adults. Are frail people all the same? | Montero-Odasso et al. (2009) | Yes | Mortality, institutionalization, hip fracture, and hospitalization | Yes | Yes | Nil | Yes |
|  |  |  |  |  |  |  |  |
| Identifying Patterns of Eating and Physical activity in children: A Latent class analysis of Obesity Risk | Huh et al. (2010) | Yes | BMI | Yes | Yes | Nil | Yes |
|  |  |  |  |  |  |  |  |
| Identifying risk profiles for childhood obesity using recursive partitioning based on individual, familial, and neighborhood environment factors | Hulst et al. (2015) | Yes | Nil | Yes | Yes | Nil | Yes |
|  |  |  |  |  |  |  |  |
| Is depression associated with health risk-related behavior clusters in adults? | Verger et al. (2009) | Yes | Depression | Yes | Yes | Nil | Yes |
|  |  |  |  |  |  |  |  |
| Latent class analysis applied to health behaviors | Ingledew et al. (1995) | Yes | Nil | Yes | Yes | Nil | Nil |
|  |  |  |  |  |  |  |  |
| Latent Class Analysis of Lifestyle Characteristics and Health Risk Behaviors among College Youth | Laska et al. (2009) | Yes | Nil | Yes | Yes | Nil | Yes |
|  |  |  |  |  |  |  |  |
| Latent Transition Analysis: Benefits of a Latent Variable Approach to Modeling Transitions in Substance Use | Lanza et al. (2010) | Yes | Nil | Yes | Yes | Yes | Yes |
|  |  |  |  |  |  |  |  |
| Neighborhood Environment Profiles for Physical Activity Among Older Adults | Adams et al. (2012) | Yes | Physical activity and BMI | Yes | Yes | Nil | Yes |
|  |  |  |  |  |  |  |  |
| Obesogenic clusters: multidimensional adolescent obesity- related behaviors in the U.S. | Boone-Heinonen et al. (2008) | Yes | Obesity incidence | Yes | Yes | Nil | Yes |
|  |  |  |  |  |  |  |  |
| Patterns of health risk behaviors among job-seekers: a latent class analysis | Schnuerer et al. (2015) | Yes | Self-rated health | Yes | Yes | Nil | Yes |
|  |  |  |  |  |  |  |  |
| Patterns of neighborhood environment attributes related to physical activity across 11 countries: a latent class analysis | Adams et al. (2013) | Yes | Physical activity | Yes | Yes | Nil | Yes |
|  |  |  |  |  |  |  |  |
| Patterns of Physical Activity, Sedentary Behavior, and Diet in U.S. Adolescents | Iannotti and Wang (2013) | Yes | Physical and psychological health (e.g. BMI and depression) | Yes | Yes | Nil | Yes |
|  |  |  |  |  |  |  |  |
| Patterns of Substance Use in Early Through Late Adolescence | Zapert, Snow, and Tebes (2002) | Yes | Nil | Yes | Yes | Nil | Yes |
|  |  |  |  |  |  |  |  |
| Physical activity and sedentary activity patterns among children and adolescents: a latent class analysis approach | Heitzler et al. (2011) | Yes | Weight status | Yes | Yes | Nil | Yes |
|  |  |  |  |  |  |  |  |
| Physical activity and sedentary behavior typologies of 10-11 year olds | Jago et al. (2010) | Yes | Nil | Yes | Yes | Nil | Yes |
|  |  |  |  |  |  |  |  |
| Recursive partitioning–based preoperative risk stratification for atrial fibrillation after coronary artery bypass surgery | Sedrakyan et al. (2006) | Yes | Nil | Yes | Yes | Nil | Yes |
|  |  |  |  |  |  |  |  |
| Risk behavior, parental background, and wealth: A cluster analysis among Swedish boys and girls in the HBSC study | Carlerby et al. (2012) | Yes | Parental background (e.g. foreigners) and family affluence | Yes | Yes | Nil | Yes |
|  |  |  |  |  |  |  |  |
| Socioeconomic differences in dietary patterns among middle-aged men and women | Martikainen et al. (2003) | Yes | Biological risk factors (e.g. serum triglyceride levels) | Yes | Yes | Nil | Yes |
|  |  |  |  |  |  |  |  |
| Symptom clustering in advanced cancer | Walsh and Rybicki (2006) | Yes | Nil | Yes | Yes | Nil | Yes |
|  |  |  |  |  |  |  |  |
| Symptom Clusters and Relationships to Symptom Interference with Daily Life in Taiwanese Lung Cancer Patients | Wang et al. (2008) | Yes | Interferences with daily life (e.g. work) | Yes | Yes | Nil | Yes |
|  |  |  |  |  |  |  |  |
| The effect of symptom clusters on functional status and quality of life in women with breast cancer | Dodd et al. (2010) | Yes | Functional status and quality of life | Yes | Yes | Nil | Yes |
|  |  |  |  |  |  |  |  |
| The influence of health behavior clusters on dietary change | Reedy et al. (2005) | Yes | Response to a diet intervention program | Yes | Yes | Nil | Yes |
|  |  |  |  |  |  |  |  |
| The internal validity of a dietary pattern analysis. The Framingham Nutrition Studies | Quatromoni et al. (2001) | Yes | Heart disease risk factors | Yes | Yes | Nil | Yes |
|  |  |  |  |  |  |  |  |
| The Structure of Posttraumatic Stress Disorder | Breslau et al. (2005) | Yes | Consequences of disturbance (e.g. assaultive violence) and persistence of symptoms (time to remission) | Yes | Yes | Nil | Yes |
|  |  |  |  |  |  |  |  |
| Tobacco, Marijuana, and Alcohol Use in University Students: A Cluster Analysis | Primack et al. (2012) | Yes | Nil | Yes | Yes | Nil | Yes |
|  |  |  |  |  |  |  |  |
| Toward an Empirical Taxonomy of Suicide Ideation: A Cluster Analysis of the Youth Risk Behavior Survey | Flannery et al. (2003) | Yes | Nil | Yes | Yes | Nil | Yes |
|  |  |  |  |  |  |  |  |
| Tracing the Mediterranean diet through principal components and cluster analyses in the Greek population | Costacou et al. (2003) | Yes | Nil | Yes | Yes | Nil | Yes |
|  |  |  |  |  |  |  |  |
| Trajectories of posttraumatic stress symptomatology in older persons affected by a large-magnitude disaster | Pietrzak et al. (2013) | Yes | Nil | Yes | Yes | Nil | Yes |
|  |  |  |  |  |  |  |  |
| Transition to College: Α Classification and Regression Tree (CART) analysis of natural reduction of binge drinking | Vik et al. (2006) | Yes | Nil | Yes | Yes | Nil | Yes |
|  |  |  |  |  |  |  |  |
| Transitions in drug use among high-risk women: an application of latent class and latent transition analysis | Lanza and Bray et al. (2010) | Yes | Nil | Yes | Yes | Yes | Yes |
|  |  |  |  |  |  |  |  |
| Types of alcoholics, I - evidence for an Empirically Derived Typology Based on Indicators of Vulnerability and Severity | Babor et al. (1992) | Yes | Alcoholism treatment outcome (e.g. total number of drinking days posttreatment) | Yes | Yes | Nil | Yes |
|  |  |  |  |  |  |  |  |
| Typologies of posttraumatic stress disorder in the U.S. adult population | Pietrzak et al. (2014) | Yes | HRQoL and suicide attempt | Yes | Yes | Nil | Yes |
|  |  |  |  |  |  |  |  |
| Typologies of posttraumatic stress disorder in treatment-seeking older adults | Böttche et al. (2015) | Yes | Nil | Yes | Yes | Nil | Yes |
|  |  |  |  |  |  |  |  |
| A latent class analysis of illicit drug abuse/dependence: results from the National Epidemiological Survey on Alcohol and Related Conditions | Agrawal et al. (2006) | Yes | Major psychiatric disorders (e.g. alcohol abuse/dependence, nicotine dependence) | Yes | Yes | Nil | Yes |
|  |  |  |  |  |  |  |  |
| A latent class analysis of underage problem drinking: Evidence from a community sample of 16–20 year olds | Reboussin et al. (2006) | Yes | Nil | Yes | Yes | Nil | Yes |
|  |  |  |  |  |  |  |  |
| A longitudinal investigation of the impact of typology of urinary incontinence on quality of life during midlife: Results from a British prospective study | Mishra et al. (2009) | Yes | HRQoL | Yes | Yes | Nil | Yes |
|  |  |  |  |  |  |  |  |
| A Longitudinal Typology of Symptoms of Depression and Anxiety Over the Life Course | Colman et al. (2007) | Yes | Nil | Yes | Yes | Nil | Yes |
|  |  |  |  |  |  |  |  |
| An Empirical Study of the Classification of Eating Disorders | Bulik, Sullivan, and Kendler (2000) | Yes | Nil | Yes | Yes | Nil | Yes |
|  |  |  |  |  |  |  |  |
| Application of a Latent Class Analysis to Empirically Define Eating Disorder Phenotypes | Keel et al. (2004) | Yes | Nil | Yes | Yes | Nil | Yes |
|  |  |  |  |  |  |  |  |
| Bone mineral density and dietary patterns in older adults: the Framingham Osteoporosis Study | Tucker et al. (2002) | Yes | BMD | Yes | Yes | Nil | Yes |
|  |  |  |  |  |  |  |  |
| Characterization of different groups of elderly according to social engagement activity patterns | Croezen et al. (2009) | Yes | Self-perceived health, mental health, and physical health, | Yes | Yes | Nil | Yes |
|  |  |  |  |  |  |  |  |
| Classes of disruptive behavior in a sample of young elementary school children | Lier et al. (2003) | Yes | Classification of disruptive disorders in DSM-IV | Yes | Yes | Nil | Yes |
|  |  |  |  |  |  |  |  |
| Classification of suicide attempters by cluster analysis: a study of the temperamental heterogeneity in suicidal patients | Engstrom et al. (1996) | Yes | Nil | Yes | Yes | Nil | Yes |
|  |  |  |  |  |  |  |  |
| Classification of Suicide Attempters by Cluster Analysis | Paykel et al. (1978) | Yes | Nil | Yes | Yes | Nil | Yes |
|  |  |  |  |  |  |  |  |
| Cluster Analysis Methods Help to Clarify the Activity–BMI Relationship of Chinese Youth | Monda and Popkin et al. (2005) | Yes | BMI | Yes | Yes | Nil | Yes |
|  |  |  |  |  |  |  |  |
| Clustering of dietary variables and other lifestyle factors (Dutch Nutritional Surveillance System) | Huishof et al. (1992) | Yes | BMI | Yes | Yes | Nil | Yes |
|  |  |  |  |  |  |  |  |
| Creating Neighborhood Typologies of GIS-Based Data in the Absence of Neighborhood-Based Sampling: A Factor and Cluster Analytic Strategy | Gershoff, Pedersen, and Aber (2009) | Yes | Nil | Yes | Yes | Nil | Yes |
|  |  |  |  |  |  |  |  |
| Developmental Typology of Trajectories to Nighttime Bladder Control: Epidemiologic Application of Longitudinal Latent Class Analysis | Croudace et al. (2002) | Yes | Nil | Yes | Yes | Nil | Yes |
|  |  |  |  |  |  |  |  |
| Dietary patterns and adenocarcinoma of the esophagus and distal stomach | Chen et al. (2002) | Yes | Risk of esophageal adenocarcinoma and distal stomach adenocarcinoma. | Yes | Yes | Nil | Yes |
|  |  |  |  |  |  |  |  |
| Dietary Patterns and Cardiovascular Risk Factors in Elderly Men: The Zutphen Elderly Study | Huijbregts et al. (1995) | Yes | Cardiovascular risk factors (e.g. BMI and serum total cholesterol level) | Yes | Yes | Nil | Yes |
|  |  |  |  |  |  |  |  |
| Dietary patterns and lifestyle factors in the Norwegian EPIC cohort: The Norwegian Women and Cancer (NOWAC) study | Engeset et al. (2005) | Yes | BMI | Yes | Yes | Nil | Yes |
|  |  |  |  |  |  |  |  |
| Dietary Patterns and Nutrient Intakes of 7-Year-Old Children Taking Part in an Atherosclerosis Prevention Project in Finland | Rasanen et al. (2002) | Yes | Cardiovascular risk factors (e.g. serum cholesterol lever) | Yes | Yes | Nil | Yes |
|  |  |  |  |  |  |  |  |
| Dietary Patterns and Survival of Older Adults | Anderson et al. (2010) | Yes | Survival over a 10-year period, HRQoL, and nutritional status (e.g. serum Vitamin B12 level) | Yes | Yes | Nil | Yes |
|  |  |  |  |  |  |  |  |
| Dietary patterns and the adenoma-carcinoma sequence of colorectal cancer | Rouillier et al. (2005) | Yes | Risk of colon adenomas and cancers | Yes | Yes | Nil | Yes |
|  |  |  |  |  |  |  |  |
| Dietary patterns are associated with lower incidence of type 2 diabetes in middle-aged women: the Shanghai Women’s Health Study | Villegas et al. (2010) | Yes | Risk of type 2 diabetes | Yes | Yes | Nil | Yes |
|  |  |  |  |  |  |  |  |
| Dietary Patterns Associated with Risk for Metabolic Syndrome in Urban Community of Karachi Defined by Cluster Analysis | Hydrie et al. (2010) | Yes | Risk of metabolic syndrome | Yes | Yes | Nil | Yes |
|  |  |  |  |  |  |  |  |
| Dietary patterns in middle-aged Irish men and women deﬁned by cluster analysis | Villegas et al. (2004) | Yes | Cardiovascular risk factors (e.g. BMI and waist circumference) | Yes | Yes | Nil | Yes |
|  |  |  |  |  |  |  |  |
| Dietary patterns in the Southampton Women’s Survey | Crozier et al. (2006) | Yes | Nil | Yes | Yes | Nil | Yes |
|  |  |  |  |  |  |  |  |
| Dietary patterns of elderly Boston­area residents defined by cluster analysis | Tucker et al. (1992) | Yes | BMI and blood profile (e.g. blood nutrient and cholesterol levels) | Yes | Yes | Nil | Yes |
|  |  |  |  |  |  |  |  |
| Dietary Patterns of Hispanic Elders Are Associated with Acculturation and Obesity | Lin, Bermudez, and Tucker (2013) | Yes | BMI and waist circumference | Yes | Yes | Nil | Yes |
|  |  |  |  |  |  |  |  |
| Dietary patterns of men and women suggest targets for health promotion- the Framingham Nutrition Studies | Millen et al. (1996) | Yes | Nil | Yes | Yes | Nil | Yes |
|  |  |  |  |  |  |  |  |
| Dietary Patterns of Rural Older Adults Are Associated with Weight and Nutritional Status | Ledikwe et al. (2004) | Yes | BMI, waist circumference, and plasma biomarkers (e.g. plasma folate level) | Yes | Yes | Nil | Yes |
|  |  |  |  |  |  |  |  |
| Dietary patterns predict the development of overweight in women- The Framingham nutrition studies. | Quatromoni et al. (2002) | Yes | Risk of overweight | Yes | Yes | Nil | Yes |
|  |  |  |  |  |  |  |  |
| Disentangling women’s responses on complex dietary intake patterns from an Indian cross-sectional survey: a latent class analysis | Padmadas, Dias, and Willekens (2006) | Yes | Nil | Yes | Yes | Nil | Yes |
|  |  |  |  |  |  |  |  |
| Distinct clinical phenotypes of airways disease defined by cluster analysis | Weatherall et al. (2009) | Yes | Healthcare utilization (e.g. Hospitalization), treatment plans (e.g. inhaled corticosteroids) etc. | Yes | Yes | Nil | Yes |
|  |  |  |  |  |  |  |  |
| Empirically derived symptom sub-groups correspond poorly with diagnostic criteria for functional dyspepsia and irritable bowel syndrome. A factor and cluster analysis of a patient sample | Eslick et al. (2003) | Yes | Nil | Yes | Yes | Nil | Yes |
|  |  |  |  |  |  |  |  |
| Gastrointestinal Symptoms and Subjects Cluster Into Distinct Upper and Lower Groupings in the Community: A Four Nations Study | Talley et al. (2000) | Yes | International Rome classiﬁcation for separate upper and lower functional GI disorders. | Yes | Yes | Nil | Yes |
|  |  |  |  |  |  |  |  |
| Identification and Prediction of Latent Classes of Weight-loss Strategies Among Women | Lanza, Savage, and Birch (2010) | Yes | Nil | Yes | Yes | Nil | Yes |
|  |  |  |  |  |  |  |  |
| Identification of groups who report similar patterns of diet among a representative national sample of British adults aged 65 years of age or more | Pryer, Cook, and Shetty (2000) | Yes | Blood tests (e.g. serum folate level) and BMI | Yes | Yes | Nil | Yes |
|  |  |  |  |  |  |  |  |
| Identifying dietary patterns using a normal mixture model: application to the EPIC study | Fahey et al. (2011) | Yes | BMI | Yes | Yes | Nil | Yes |
|  |  |  |  |  |  |  |  |
| Identifying target segments of male drinkers for health promotion | Wyllie and Casswell (1993) | Yes | Nil | Yes | Yes | Nil | Yes |
|  |  |  |  |  |  |  |  |
| Latent Class Analysis Is Useful to Classify Pregnant Women into Dietary Patterns | Sotres-Alvarez, Herring, and Siega-Riz (2010) | Yes | Nil | Yes | Yes | Nil | Yes |
|  |  |  |  |  |  |  |  |
| Latent Class Analysis of Lifetime Depressive Symptoms in the National Comorbidity Survey | Sullivan, Kessler, and Kendler (1998) | Yes | Consequences of the disease (e.g. hospitalization) | Yes | Yes | Nil | Yes |
|  |  |  |  |  |  |  |  |
| Latent Transition Models to Study Women’s Changing of Dietary Patterns From Pregnancy to 1 Year Postpartum | Sotres-Alvarez, Herring, and Siega-Riz (2013) | Yes | Nil | Yes | Yes | Yes | Yes |
|  |  |  |  |  |  |  |  |
| Leisure-time physical activity and sedentary behavior clusters and their associations with overweight in middle-aged French adults | Charreire et al. (2010) | Yes | BMI | Yes | Yes | Nil | Yes |
|  |  |  |  |  |  |  |  |
| Patterns of health-related behavior and their cross-cultural validity - A comparative study on two populations of young people | Karvonen et al. (2000) | Yes | Nil | Yes | Yes | Nil | Yes |
|  |  |  |  |  |  |  |  |
| Relationships of dietary patterns with body composition in older adults differ by gender and PPAR-γ Pro12Ala genotype | Anderson et al. (2010) | Yes | Body composition (e.g. total body fat) | Yes | Yes | Nil | Yes |
|  |  |  |  |  |  |  |  |
| Resilience and patterns of health risk behaviors in California adolescents | Mistry et al. (2009) | Yes | Nil | Yes | Yes | Nil | Yes |
|  |  |  |  |  |  |  |  |
| Seven unique food consumption patterns identiﬁed among women in the UK Women’s Cohort Study | Greenwood et al. (2000) | Yes | BMI | Yes | Yes | Nil | Yes |
|  |  |  |  |  |  |  |  |
| Subgroups of Patients With Cancer With Different Symptom Experiences and Quality-of-Life Outcomes: A Cluster Analysis | Miaskowski et al. (2006) | Yes | Functional status and HRQoL | Yes | Yes | Nil | Yes |
|  |  |  |  |  |  |  |  |
| The Structure of Psychosis - Latent Class Analysis of Probands From the Roscommon Family Study | Kendler et al. (1998) | Yes | Nil | Yes | Yes | Nil | Yes |
|  |  |  |  |  |  |  |  |
| Using Cluster Analysis to Examine Dietary Patterns- Nutrient Intakes, Gender, and Weight Status Differ Across Food Pattern Clusters | Wirfalt and Jeffery (1997) | Yes | BMI | Yes | Yes | Nil | Yes |
|  |  |  |  |  |  |  |  |
| A Latent Class Analysis of Stigmatizing Attitudes and Knowledge of HIV Risk among Youth in South Africa | Brinkley-Rubinstein et al. (2014) | Yes | Stigmatizing attitudes toward HIV positive population | Yes | Yes | Nil | Yes |
|  |  |  |  |  |  |  |  |
| A person-centred segmentation study in elderly care: Towards efﬁcient demand-driven care | Laan et al. (2014) | Yes | Healthcare utilization | Yes | Yes | Nil | Yes |
|  |  |  |  |  |  |  |  |
| Health status transitions in community-living elderly with complex care needs: a latent class approach | Lafortune et al. (2009) | Yes | Nil | Yes | Yes | Yes | Yes |
|  |  |  |  |  |  |  |  |
| Heterogeneity of severe asthma in childhood: Conﬁrmation by cluster analysis of children in the National Institutes of Health/National Heart, Lung, and Blood Institute Severe Asthma Research Program | Fitzpatrick et al. (2011) | Yes | Current deﬁnitions of asthma severity (e.g. ATS criteria and GINA or criteria) in asthma treatment guidelines | Yes | Yes | Nil | Yes |
|  |  |  |  |  |  |  |  |
| The Clustering of Health Behaviors in Older Australians and its Association with Physical and Psychological Status, and Sociodemographic Indicators | Griffin et al. (2014) | Yes | Physical and psychological health morbidity (e.g. BMI, quality of life) | Yes | Yes | Nil | Yes |
|  |  |  |  |  |  |  |  |
| The heterogeneous health latent classes of elderly people and their socio-demographic characteristics in Taiwan | Liu et al. (2014) | Yes | Nil | Yes | Yes | Nil | Yes |
|  |  |  |  |  |  |  |  |
| Utilization of health care services by elderly people with National Health Insurance in Taiwan: The heterogeneous health proﬁle approach | Liu et al. (2012) | Yes | Healthcare services utilization and expenditures | Yes | Yes | Nil | Yes |
|  |  |  |  |  |  |  |  |
| A Cluster Analysis of Physical Activity and Sedentary Behavior Patterns in Middle School Girls | Trilk et al. (2012) | Yes | Objectively measured physical activity | Yes | Yes | Nil | Yes |
|  |  |  |  |  |  |  |  |
| A hierarchy of sociodemographic and environmental correlates of walking and obesity | Frank et al. (2008) | Yes | Nil | Yes | Yes | Nil | Yes |
|  |  |  |  |  |  |  |  |
| A Latent Class Analysis of DSM-IV and Fagerström (FTND) Criteria for Nicotine Dependence | Agrawal et al. (2011) | Yes | Smoking-related outcomes (e.g. maximum cigarettes smoked in 24 hours), other psychopathology (e.g. Major depressive disorder) | Yes | Yes | Nil | Yes |
|  |  |  |  |  |  |  |  |
| A Latent Class Analysis of Risk Factors for Acquiring HIV Among Men Who Have Sex with Men: Implications for Implementing Pre-Exposure Prophylaxis Programs | Chan et al. (2015) | Yes | HIV related outcomes (e.g. testing positive for HIV or another sexually transmitted diseases) | Yes | Yes | Nil | Yes |
|  |  |  |  |  |  |  |  |
| A latent class model to identify city/town chronic disease patterns | Jiang et al. (2015) | Yes | Nil | Yes | Yes | Nil | Yes |
|  |  |  |  |  |  |  |  |
| A Latent Transition Model of the Effects of a Teen Dating Violence Prevention Initiative | Williams et al. (2015) | Yes | Nil | Yes | Yes | Yes | Yes |
|  |  |  |  |  |  |  |  |
| Adolescent physical activity and the built environment: A latent class analysis approach | McDonald et al. (2012) | Yes | Youth physical activities (e.g. Mean minutes/day of activity, Mean minutes/day of sedentary time Mean screen time, min/day) | Yes | Yes | Nil | Yes |
|  |  |  |  |  |  |  |  |
| Comparison of Suicide Attempters and Decedents in the U.S. Army: A Latent Class Analysis | Skopp et al. (2016) | Yes | Nil | Yes | Yes | Yes | Yes |
|  |  |  |  |  |  |  |  |
| Complex Comorbidity Clusters in OEF/OIF Veterans - The Polytrauma Clinical Triad and Beyond | Pugh et al. (2014) | Yes | Medication use (e.g. opioid pain reliever), acute health care utilization (e.g. emergency/urgent care), and adverse outcomes (e.g. suicide-related behaviors) | Yes | Yes | Nil | Yes |
|  |  |  |  |  |  |  |  |
| Coping, Stress, and Social Support Associations With Internalizing and Externalizing Behavior Among Urban Adolescents and Young Adults: Revelations From a Cluster Analysis | Tandon et al. (2013) | Yes | Depressive symptoms, suicidality, and violence perpetration | Yes | Yes | Nil | Yes |
|  |  |  |  |  |  |  |  |
| Differences in environmental preferences towards cycling for transport among adults: a latent class analysis | Mertens et al. (2016) | Yes | Transport behavior (e.g. Bicycle leisure time min/wk) | Yes | Yes | Nil | Yes |
|  |  |  |  |  |  |  |  |
| Distinct symptom experiences in subgroups of patients with COPD | Christensen et al. (2016) | Yes | Clinical characteristics (e.g. airflow limitation) and disease-specific quality of life scores | Yes | Yes | Nil | Yes |
|  |  |  |  |  |  |  |  |
| Effects of clustering of multiple lifestyle-related behaviors on blood pressure in adolescents from two observational studies | Moraes et al. (2016) | Yes | Systolic BP (SBP) and diastolic BP (DBP) | Yes | Yes | Nil | Yes |
|  |  |  |  |  |  |  |  |
| Exploring the application of latent class cluster analysis for investigating pedestrian crash injury severities in Switzerland | Sasidharan et al. (2015) | Yes | Nil | Yes | Yes | Yes | Yes |
|  |  |  |  |  |  |  |  |
| Health lifestyle behaviors among U.S. adults | Onge and Krueger (2017) | Yes | Mortality | Yes | Yes | Nil | Yes |
|  |  |  |  |  |  |  |  |
| Health-Related Fitness Proﬁles in Adolescents With Complex Congenital Heart Disease | Klausen et al. (2015) | Yes | Lifestyle behaviors (e.g. Participate in Physical Education at school) | Yes | Yes | Nil | Yes |
|  |  |  |  |  |  |  |  |
| Latent class analysis of acceptability and willingness to pay for self-HIV testing in a United States urban neighborhood with high rates of HIV infection | Nunn et al. (2017) | Yes | Attitudes about and willingness to buy HIV self-tests | Yes | Yes | Nil | Yes |
|  |  |  |  |  |  |  |  |
| Latent class modelling of the association between socioeconomic background and breast cancer survival status at 5 years incorporating stage of disease | Downing et al. (2010) | Yes | Death within 5 years | Yes | Yes | Nil | Yes |
|  |  |  |  |  |  |  |  |
| Latent Classes of Young adults Based on Use of Multiple types of tobacco and nicotine Products | Erickson, Lenk, and Forster (2014) | Yes | Marijuana and alcohol use | Yes | Yes | Nil | Yes |
|  |  |  |  |  |  |  |  |
| Latent Homeless Risk Proﬁles of a National Sample of Homeless Veterans and Their Relation to Program Referral and Admission Patterns | Tsai, Kasprow, and Rosenheck (2013) | Yes | Admissions to Department of Veterans Affairs (VA) homeless service programs. | Yes | Yes | Nil | Yes |
|  |  |  |  |  |  |  |  |
| Latent variable mixture models to test for differential item functioning: a population- based analysis | Wu et al. (2017) | Yes | Nil | Yes | Yes | Nil | Yes |
|  |  |  |  |  |  |  |  |
| Pattern Analysis of Suicide Mortality Surveillance Data in Urban South Africa | Burrows and Laflamme (2008) | Yes | Nil | Yes | Yes | Nil | Yes |
|  |  |  |  |  |  |  |  |
| Patterns of Alternative Tobacco Product Use: Emergence of Hookah and E-cigarettes as Preferred Products Amongst Youth | Gilreath et al. (2016) | Yes | Nil | Yes | Yes | Nil | Yes |
|  |  |  |  |  |  |  |  |
| Patterns of Hospitalization Risk for Women Surviving Into Very Old Age | Dolja-Gore et al. (2017) | Yes | Health-related quality of life, number comorbidities, health behaviors etc. | Yes | Yes | Nil | Yes |
|  |  |  |  |  |  |  |  |
| Patterns of neighborhood environment attributes in relation to children's physical activity | Kurka et al. (2015) | Yes | Moderate-to-vigorous physical activity | Yes | Yes | Nil | Yes |
|  |  |  |  |  |  |  |  |
| Patterns of Physical Activity, Sedentary Behavior and Diet in US Adolescents | Iannotti and Wang (2013) | Yes | Weight Status, Losing Weight Behaviors, Depression and Frustration of Physical Appearance | Yes | Yes | Nil | Yes |
|  |  |  |  |  |  |  |  |
| Prevalence and Patterns of Polysubstance Use in a Nationally Representative Sample of 10th Graders in the United States | Conway et al. (2013) | Yes | Somatic and depressive symptoms (e.g. depression) | Yes | Yes | Nil | Yes |
|  |  |  |  |  |  |  |  |
| Smoking Patterns and their relationship to Drinking among First-Year College students | Hoeppner et al. (2014) | Yes | Alcohol Use | Yes | Yes | Nil | Yes |
|  |  |  |  |  |  |  |  |
| Smoking patterns during pregnancy and postnatal period and depressive symptoms | Munafo, Heron, and Araya (2007) | Yes | Psychosocial adversities (e.g. financial difficulties) and depression symptom score | Yes | Yes | Nil | Yes |
|  |  |  |  |  |  |  |  |
| Subgrouping outpatients of an environmental medicine unit using SCL-90-R and cluster analysis | Helm and Eis (2007) | Yes | Nil | Yes | Yes | Nil | Yes |
|  |  |  |  |  |  |  |  |
| The Neighbourhood Built Environment and Trajectories of Depression Symptom Episodes in Adults: A Latent Class Growth Analysis | Gariepy et al. (2015) | Yes | Nil | Yes | Yes | Nil | Yes |
|  |  |  |  |  |  |  |  |
| Time Use and Food Pattern Influences on Obesity | Kolodinsky and Goldstein (2011) | Yes | Probability of overweight and BMI | Yes | Yes | Nil | Yes |
|  |  |  |  |  |  |  |  |
| Tobacco Use and Suicidality: Latent Patterns of Co-occurrence Among Black Adolescents | Gilreath, Connell, and Leventhal (2012) | Yes | Suicidality latent class membership for suicidality segmentation and vice versa | Yes | Yes | Nil | Yes |
|  |  |  |  |  |  |  |  |
| Trajectories and predictors of return to work after traumatic limb injury – a 2-year follow-up study | Hou et al. (2012) | Yes | Biopsychosocial factors (e.g. injury severity, depression status, disturbance in daily life, self-efficacy, and quality of life) | Yes | Yes | Nil | Yes |
|  |  |  |  |  |  |  |  |
| Trajectories of Cigarette smoking From adolescence to adulthood as Predictors of Unemployment status | Brook et al. (2014) | Yes | Unemployment in the fifth decade of life | Yes | Yes | Nil | Yes |
|  |  |  |  |  |  |  |  |
| Trajectories of Depressive Symptoms Among Web-Based Health Risk Assessment Participants | Bedrosian, Hawrilenko, and Cole-Lewis (2017) | Yes | Quality of life, current treatment | Yes | Yes | Nil | Yes |
|  |  |  |  |  |  |  |  |
| Trajectories of Depressive Symptoms in Canadian Emerging Adults | Ferro, Gorter, and Boyle (2015) | Yes | Other health related characteristic (e.g. socioeconomic status, interpersonal relations, and health conditions) | Yes | Yes | Nil | Yes |
|  |  |  |  |  |  |  |  |
| Trajectories of Kinematic Risky Driving Among Novice Teenagers | Simons-Morton et al. (2013) | Yes | Risky driving related characteristics (e.g. self-reported risky driving) | Yes | Yes | Nil | Yes |
|  |  |  |  |  |  |  |  |
| Trajectories of Loneliness in Adolescents With Congenital Heart Disease: Associations With Depressive Symptoms and Perceived Health | Vanhalst et al. (2013) | Yes | Depressive symptoms (Center for Epidemiologic Studies Depression Scale) and perceived health (Pediatric Quality of Life Inventory 3.0) | Yes | Yes | Nil | Yes |
|  |  |  |  |  |  |  |  |
| Trajectories of physical functioning and their prognostic indicators: A prospective cohort study in older adults with joint pain and comorbidity | Hermsen et al. (2014) | Yes | Sociodemographic, physical and psychosocial indicators (e.g. depressive symptoms, perceived self-efﬁcacy) | Yes | Yes | Nil | Yes |
|  |  |  |  |  |  |  |  |
| Trajectories of productivity loss over a 20-year period: an analysis of the National Longitudinal Survey of Youth | Besen and Pransky (2014) | Yes | Productivity related characteristics (e.g. employment outcomes in midlife, self-esteem) | Yes | Yes | Nil | Yes |
|  |  |  |  |  |  |  |  |
| Trajectories of Social Engagement and Limitations in Late Life | Thomas (2011) | Yes | Cognitive and Physical Limitations | Yes | Yes | Nil | Yes |
|  |  |  |  |  |  |  |  |
| Trajectories of Suicidal Ideation from Sixth through Tenth Grades in Predicting Suicide Attempts in Young Adulthood in an Urban African American Cohort | Musci et al. (2016) | Yes | Suicide attempts in young adulthood | Yes | Yes | Nil | Yes |
|  |  |  |  |  |  |  |  |
| Trajectories or Parental Monitoring and Communication and Effects on Drug Use Among Urban Young Adolescents | Tobler and Komro (2010) | Yes | Drug use in 8th grade | Yes | Yes | Nil | Yes |
|  |  |  |  |  |  |  |  |
| Trajectory Classes of Decline in Health-Related Quality of Life in Parkinson’s Disease: A Pilot Study | Klotsche et al. (2011) | Yes | Other disease related characteristics (e.g. cumulative mortality) | Yes | Yes | Nil | Yes |
|  |  |  |  |  |  |  |  |
| Transitions in Smokers’ Social Networks After Quit Attempts: A Latent Transition Analysis | Bray et al. (2016) | Yes | Nil | Yes | Yes | Yes | Yes |
|  |  |  |  |  |  |  |  |
| Transitions in Suicide Risk in a Nationally Representative Sample of Adolescents | Thompson, Kuruwita, and Foster (2009) | Yes | Probability of making a suicide attempt | Yes | Yes | Yes | Yes |
|  |  |  |  |  |  |  |  |
|  |  |  |  |  |  |  |  |

**Abbreviations:**

GP: General Practitioner US: United States UK: United Kingdom GIS: Geographic information systems PTSD: Post-traumatic stress disorder BMI: Body mass index SBP: Systolic blood pressure DBP: Diastolic blood pressure IBS: Irritable bowel syndrome FEV1: Forced expiratory volume during the 1st second AF: Atrial fibrillation CABG: Coronary artery bypass grafting HIV: human immunodeficiency virus HRQoL: Health related quality of life BMD: Bone mineral density DSM: Diagnostic and Statistical Manual of Mental Disorders; COPD: chronic obstructive pulmonary disease;
